# Salidroside Improves Chronic Stress Induced Depressive Symptoms Through Microglial Activation Suppression

**DOI:** 10.3389/fphar.2021.635762

**Published:** 2021-06-08

**Authors:** Yang Fan, Yajuan Bi, Haixia Chen

**Affiliations:** School of Pharmaceutical Science and Technology, Tianjin University, Tianjin, China

**Keywords:** salidroside, neuroinflammation, microglia, depressive behavior, LPS

## Abstract

Depression is a severe neurological disorder highly associated with chronic mental stress stimulation, which involves chronic inflammation and microglial activation in the central nervous system (CNS). Salidroside (SLDS) has been reported to exhibit anti-neuroinflammatory and protective properties on neurological diseases. However, the mechanism underlying the effect of SLDS on depressive symptoms has not been well elaborated. In the present study, the effects of SLDS on depressive behaviors and microglia activation in mice CNS were investigated. Behavioral tests, including Forced swimming test (FST), Open field test (OFT) and Morris water maze (MWM) revealed that SLDS treatment attenuated the depressive behaviors in stress mice. SLDS treatment significantly reduced the microglial immunoreactivity for both Iba-1 and CD68, characteristic of deleterious M1 phenotype in hippocampus of stress mice. Additionally, SLDS inhibited microglial activation involving the suppression of ERK1/2, P38 MAPK and p65 NF-κB activation and thus reduced the expression and release of neuroinflammatory cytokines in stress mice as well as in lipopolysaccharide (LPS)-induced primary microglia. Also, SLDS changed microglial morphology, attachment and reduced the phagocytic ability in LPS-induced primary microglia. The results demonstrated that SLDS treatment could improve the depressive symptoms caused by unpredictable chronic stress, indicating a potential therapeutic application of SLDS in depression treatment by interfering microglia-mediated neuroinflammation.

## Introduction

Based on the statistical analysis from the World Health Organization, over 450 million people suffer from depression and it will rise to the top financial burden among all the diseases in 2030 (Ustun et al., 2004; Smith 2014; Kato and Kanba, 2015). Typical clinical symptoms of depression include significant and long-term depressive mood, insensitivity, anhedonia and vital exhaustion (Ellis et al., 2004; Szatmari et al., 2018). Further progression may even lead to suicidal tendencies (Mitchell and Malhi 2004). The pathogenesis of depression is still unclear, however multiple studies suggested that chronic mental stress might be one of the critical pathogenic factors during depression formation and progression (Patriquin and Mathew 2017; da Estrela et al., 2020). Recent studies have shown that long-term stress impacts multiple aspects of biological systems, such as neuroendocrine, autonomic regulation, and behaviors (Sterlemann et al., 2008). This long-term stimulation eventually leads to a failure in normal stress responses and brain inflammation, and finally causes mental disorders (Goshen et al., 2008; Patriquin and Mathew 2017).

Many studies have revealed that microglia cells are involved in the formation and development of depression (Singhal and Baune 2017; Deng et al., 2020). Neuroinflammation is a key driver of the pathological process in depression, and is primarily regulated by resident macrophages, which are microglia in the central nervous system (CNS). Microglia normally maintains ramified morphology in the resting state. Upon brain injury, microglia can be activated and transit into the amoeboid morphology, followed by release of multiple pro-inflammatory cytokines which further damage to nearby neurons (Fan et al., 2017). Microglia activation involves two functionally different states, deleterious state (M1 phenotype) and beneficial state (M2 phenotype) depending on the activation conditions and methods. In the presence of lipopolysaccharide (LPS), microglia cells are activated to M1 phenotype and lead to produce and secrete pro-inflammatory cytokines such as TNF-α and IL-6, IL-18, NO, and ROS (He et al., 2019; Laffer et al., 2019; Ahmed et al., 2021; Zong et al., 2020). In contrast, IL-4 or IL-10 induce microglia cells into M2 phenotype which tunes down the M1 phenotype and secretes brain-derived neurotrophic factor (BDNF), transforming growth factor-β (TGF-β) and nerve growth factor (NGF) to repair tissue and extracellular matrix components in CNS(Wei and Jonakait 1999; Szczepanik et al., 2001; Laffer et al., 2019; Yi et al., 2020). LPS is a classic inflammatory trigger that activates M1 phenotype, induces inflammatory response, further leading to inflammatory cytokines expression alterations and cerebral injuries (Nakagawa and Chiba 2014). Iba1 is the protein which widely and specifically expressed in the microglia all over the CNS and used as a microglia marker, while CD68 is a transmembrane protein widely expressed in activated microglia (M1 phenotype) upon LPS induction (Leaw et al., 2017). The morphological changes of microglia cells are closely related to their activation state and are the first sign to be observed in the multi-step microglia activation. These changes include thickening and retraction of processes and the increase in cell body size (Tynan et al., 2010; Fan et al., 2018). Vimentin filaments are parts of the intermediate filament system whose dynamic properties are important for cellular flexibility and vimentin has been revealed as a key component involved in microglia activation (Jiang et al., 2013). Focal adhesions refer to the physical connection between the extracellular matrix and the cell actin cytoskeleton, which are mediated by integrins. They are of fundamental importance in regulating cellular adhesion, mechanical sensing, and signals controlling for cell growth and differentiation (Ilic et al., 1995; Ruggiero et al., 2018). Paxillin has been reported to be phosphorylated by p38 MAPK and ERK1/2 at the residue Ser83 and its phosphorylation is involved in microglial activation and phagocytosis (Huang et al., 2004; Fan et al., 2018). Hippocampus is one of the important functional area of the brain which is responsible for the storage transformation of long-term memory and orientation (Colombo et al., 2001; Shi et al., 2018). Recent research has also highlighted its strong functional linkage to anxiety and depressive behaviors (Lee et al., 2002; Feng et al., 2020). The activation of microglia is pathological characteristics of patients with depression, which results in the release of extensive levels of proinflammatory cytokines in brain and causes neuronal apoptosis (Jiang et al., 2020; Wang T., et al., 2020). Therefore, inhibition of the deleterious microglial activation and migration might be a promising therapeutic strategy to ameliorate depressive symptoms. However, it is still in shortage of clinical drug alternatives.

Salidroside (SLDS), a phenolic glycoside compound, is extracted from a traditional Chinese medicinal plant, *Rhodiola rosea*. For centuries, this herb has been widely used by Chinese to treat multiple inflammatory diseases. Modern investigations have shown that SLDS has potent protective effects against hepatitis, colitis, skeletal muscle atrophy, and myocardial injury by alleviating excessive inflammation (Hu B et al., 2014; Huang et al., 2019; Liu et al., 2019; Wang N. et al., 2020). Especially, SLDS plays a neuroprotective role in both preclinical models of Alzheimer disease and cerebral ischemia by regulating microglia activation and distribution (Zhang et al., 2016; Liu et al., 2018; Wang et al., 2018; Zuo et al., 2018; Wang H. et al., 2020). Studies showed that SLDS could reduce the blood-brain barrier injury by activating the PI3K/Akt signaling pathway, decrease microvascular endothelial cells apoptosis, increase neuron cells viability and promote M2 macrophage/microglial polarization, thus improving functional recovery after cerebral ischemia (Liuet al., 2018; Wang et al., 2018; Zuo et al., 2018). In addition, SLDS could improve learning and memory impairment by suppressing SIRT1/NF-κB pathway and inhibiting the release of TNF-α, IL-1β and IL-6 (Gao et al., 2016). All these studies indicated that SLDS might become a promising new implement in attenuating depression by modulating microglial activation. Therefore, the goal of this study was to open up new horizons for the medicinal value of SLDS against depression by examining behavioral effects of SLDS and its effects in microglial cell cultures.

## Materials and Methods

### Chemicals

SLDS (98% purity; Solarbio) was dissolved in phosphate buffered saline (PBS) at 50 μm. LPS (≥99% purity; Solarbio) was suspended in PBS at 1 mg/ml.

### Animals

Male C57BL/6 mice (20 ± 2 g, 6 weeks old) were purchased from HuaFuKang (Beijing, China) and raised at Institute of Radiation Medicine (Tianjin, China). All the mice were raised individually at 22 ± 1°C, at humidity of 40–50%, with 12 h light/dark cycle. Free access to water and food. Body weight was measured every week. The animal experiments were all proved by the Institutional Animal Care and Use Committee of Institute of Radiation Medicine, Chinese Academy of Medical Sciences.

### Chronic Stress Procedure and Drug Treatment

The mice were divided into three groups (10 mice each). Both stress groups were exposed to unpredictable stressors for 28 days using a strategy shown in Table 1 (Nollet et al., 2013; Willner 2017). Random stress strategies include: 24 h Food fasting; 24 h Water fasting; 12 h 50 ml centrifuge tube confinement; 24 h wet mattress; 24 h dirt cage; 30 min hot temperature; 15 min/2 times home cage shake. After 14 days, stressed groups began the intraperitoneal injection with PBS (100 μL/mice) or SLDS (10 mg/kg) every day.

**TABLE 1:** Chronic stress strategies.

Days	Chronic stress strategies	Injection
1	Change to new cage and Food fasting	no
2	Shaking home cage and Water fasting	no
3	50 ml centrifuge constraint and Wet mattress	no
4	Switch to dirty cage and Hot temperature	no
5	Shaking home cage and Food fasting	no
6	50 ml centrifuge constraint and Water fasting	no
7	Rest and Change to new cage	no
8	Hot temperature and Food fasting	no
9	Switch to dirty cage and Water fasting	no
10	50 ml centrifuge constraint and Hot temperature	no
11	Switch to dirty cage and Food fasting	no
12	Shaking home cage and Water fasting	no
13	50 ml centrifuge constraint and Food fasting	no
14	Rest and Change to new cage	no
15	Shaking home cage and Food fasting	yes
16	Switch to dirty cage and Water fasting	yes
17	Hot temperature and Food fasting	yes
18	50 ml centrifuge constraint and Water fasting	yes
19	Shaking home cage and Hot temperature	yes
20	Hot temperature and Water fasting	yes
21	Rest and Change to new cage	yes
22	50 ml centrifuge constraint and Hot temperature	yes
23	Switch to dirty cage and Food fasting	yes
24	Shaking home cage and Water fasting	yes
25	50 ml centrifuge constraint and Food fasting	yes
26	Hot temperature and Water fasting	yes
27	Shaking home cage and Switch to dirty cage	yes
28	Rest and Change to new cage	yes

### Sucrose Preference Test

Socrose preference test (SPT) was conducted according to the method described previously (Feng et al., 2020). They were tested on the initial days (the 1^st^ day and the day before it) and final days (the 28^th^ day and the day after it). Mice were adjusted to habituate to the presence of two identical drinking bottles before it started. On the day of the tests, mice were exposed to the identical bottles randomly and individually, with one-containing 1% sucrose and another tap water for 2 days. After chronic stress procedure, test was repeat again. The position of the two bottles (right/left) was varied randomly from trial to trial to prevent place-preference by the animals. The percentage of sucrose intake over total intake was calculated as the relative sucrose intake preference.

### Forced Swimming Test

FST was conducted as described by Feng et al. (Feng et al., 2020). It has been recognized as one of the most common animal models for the evaluation of antidepressant-like activity in rodents, due to its sensitivity to a broad range of antidepressant drugs (Cryan et al., 2002; Taliaz et al., 2010). The most important advantage of FST is that it is easy to operate and the data is collected and analyzed quickly. The mice were tested on the initial day (the 1^st^ day) and final day (the 28^th^ day). Briefly, mice were put into the transparent plastic buckets gently and separately. The transparent plastic buckets (30 cm in height × 20 cm in diameter) were filled with water of 12 cm high and maintained at 25 ± 2°C. Mice were kept in the water for 6 min and their behaviors were recorded in the last 4 min. When a mouse floated upright and hold its head above the water with a small amount of movements, it can be considered to be immobile.

### Open Field Test

OFT was conducted the day after FST as described (Sevastre-Berghian et al., 2020). OFT has been reported as a scientifically valid method to evaluate the general locomotivity and anxiety levels in animal experiments (Sevastre-Berghian et al., 2020; Zhang et al., 2020). Briefly, the open field (Techman software, China) 36 cm × 36 cm was surrounded by walls. The open field area was divided into 25 same small squares with lines. Each mouse was placed in the middle of the area and started to record. Mice were allowed to freely explore the new environment for 5 min. Data were obtained and analyzed using the Techman software Behavior analyzing system.

### Morris Water Maze Test

MWM was conducted the day after OFT as described (Bassett et al., 2021). Briefly, in a round water tank (30 cm in height × 100 cm in diameter) filled with water (25 ± 1°C) that was dyed white with milk, a platform was hidden in the water at a specific position. The tank was divided into four quadrants and marked 1, 2, 3 and 4. Mice were given free swimming until they reach the platform. Mice were guided manually to the platform where they were allowed to stay 60 s if the mice couldn’t reach the platform in 90 s. After 6 days of training, the mice were tested on the 7^th^ day with the platform being removed. The mice were allowed to swim freely for 60 s to test their spatial memory for the location of removed platform. Data were obtained and analyzed using the Top Scan Lite-Top View Behavior analyzing system (Noldus Information Technology, United States).

### Spontaneous Locomotor Activity Test

Spontaneous locomotor activity test (SLAT) was conducted at the days before and after treatment (Nicolas et al., 2006). Mice were treated with either vehicle (Con group) or 50 μm SLDS (SLDS group) for 7 consecutive days. Data were obtained and analyzed using a Techman software Behavior analyzing system for 10 min in an empty box (36 cm × 36 cm with walls surrounded).

### Brain Tissue Collection

After behavioral experiments, mice were anesthetized and killed. The brain was dissected and split along the longitudinal fissure into the left and right hemispheres. The hippocampus from the right hemispheres was evenly segmented and homogenized for ELISA, western blotting or qRT-PCR, while the left hemispheres were fixed in 4% paraformaldehyde at 4°C to further process for immunofluorescence staining.

### Cell Culture

Brain tissues of newborn mice C57BL/6 were cut in pieces with scissors and further dissociated by pipetting. Cells pelleted by centrifuge were suspended in DMEM with 10% fetal bovine serum premium (AusGeneX PTY LTD, Australia) and plated in culture flask. After incubation at 37°C with 5% CO_2_ for 12 days, primary microglia cells were taken off by shaking at 280–310 rpm for 1 h and seeded on the new plates. When cells reached 80% confluence, 1 μg/ml LPS alone or with 50 μM SLDS was given for 24 h.

PC12 cells (generous gift from Gao Wenyuan at Tianjin University, China) were cultured in DMEM containing 10% fetal bovine serum premium and 10% Horse Serum (Gibco, China) at 37°C with 5% CO_2_.

### Cell Viability Assay

CCK-8 kit (Biosharp, China) was utilized to estimate the cell viability according to the manufacturer's instructions. Briefly, primary microglia or PC12 cells were seeded at the density of 5 × 10^3^ cells per well. After 24 h incubation, cells were treated SLDS in different concentration (0, 50, 100, or 200 μM) with or without 1 μg/ml LPS (Wang et al., 2018) for 24 h. For apoptosis assay, primary microglia were pretreated with 50 μM SLDS with or without 1 μg/ml LPS for 12 h. Then the supernatant, defined as conditioned medium (CM) were collected to cultivate PC12 cells for 48 h (CM group) or with supplement of LPS (CM-LPS) or LPS and SLDS (CM-LPS+SLDS). After cultivation, PC12 cells were incubated with CCK-8 solution for 2 h at 37°C. The absorbance was measured at 450 nm with a microplate reader (TECAN, Switzerland).

### Western Blotting

The lysates of primary microglia or brain tissue obtained from animal experiment mentioned above (25 μg/lane) were separated by 10% SDS-PAGE and then transferred at 12V to PVDF membrane (Millipore, United States) for 70 min using a semi-dry transfer apparatus (Bio-Rad). The membranes were blocked at room temperature (RT) for 1 h with blocking buffer (Solarbio) and then incubated with different primary antibodies overnight at 4°C. Rabbit polyclonal antibodies against phospho-p42/44 MAPK, p42/44 MAPK, phospho-p38 MAPK, p38 MAPK, phospho-NF-κB p65, NF-κB p65, iNOS (1:5000, CST), phospho-paxillin Ser83 and mouse polyclonal antibodies against paxillin (1:2500, ECM biosciences), α-Tubulin (1:10000, CST) were used. After incubation with a HRP-conjugated secondary antibody, the immunoreactive signals were visualized using Amersham Imager 600 (GE Life Sciences).

### ELISA

Inflammation cytokines were measured using ELISA assay (Lanpai Bio, China). Briefly, protein concentration from brain samples or cell cultures were determined by using a BCA kit and equal amount of proteins from different samples were quantitatively analyzed with IL-1β, IL-6, IL-18 and TNF-α according to the manufacturer’s instruction.

### Real-Time PCR

Total RNA from brain samples or cell cultures was isolated by Eastep Super RNA isolation kit (Promega, China). Total RNA concentration was determined by a Nanodrop Spectrophotometer (Thermo Fisher Scientific, United States). cDNA synthesis was performed in PCR system (Eppendorf, Germany) using HiScript Q RT SuperMix kit (Vazyme, China) with 800 ng RNA in a total 10 μl reaction system. Subsequently, cDNA was diluted for 15 times and 5 μl of the dilute was mixed with Ultra SYBR mixture low ROX (CW biotech, China) for real-time PCR in a Quant Studio 6 Flex real-time PCR instrument (Thermo Fisher Scientific, China). The primers used for qRT-PCR are listed in Table 2.

**TABLE 2:** Primers used in the real-time PCR assay.

Gene		Oligonucleotide Sequence	Length (bp)
TNF-α	Forward	5′ CTC​AAG​CCC​TGG​TAT​GAG​CC 3′	20
	Reverse	5′ GGC​TGG​GTA​GAG​AAC​GGA​TG 3′	20
IL-1β	Forward	5′ CCT​GCA​GCT​GGA​GAG​TGT​GGA​T 3′	22
	Reverse	5′ TGT​GCT​CTG​CTT​GTG​AGG​TGC​T 3′	22
IL-6	Forward	5′ GGA​GCC​CAC​CAA​GAA​CGA​TA 3′	20
	Reverse	5′ CAG​GTC​TGT​TGG​GAG​TGG​TA 3′	20
IL-18	Forward	5′ ACG​TGT​TCC​AGG​ACA​CAA​CA 3′	20
	Reverse	5′ GGC​GCA​TGT​GTG​CTA​ATC​AT 3′	20
GAPDH	Forward	5′ AGC​CTC​GTC​CCG​TAG​ACA​AAA 3′	21
	Reverse	5′ TGG​CAA​CAA​TCT​CCA​CTT​TGC 3′	21

### Immunofluorescence Microscopy

Brain samples from left hemispheres were fixed in the 4% paraformaldehyde and embedded in OCT-freeze medium after sucrose cryoprotection. Brain tissues were sectioned in 25 μm thick using a cryostat (CM 1950; Germany). For the immunofluorescence assay, sections were blocked using 5% goat serum in TBS at RT for 30 min followed by the incubation with rabbit Iba-1 (Proteitech, United States) and mouse CD68 (BioLegend, United States) antibodies at 4°C overnight. After 3 washes with TBST, the sections were further incubated with corresponding secondary antibodies (Immunoway Biotech, China). After 3 times rinsing, sections were mounted with Dapi+ Fluorsave mounting medium (Calbiochem, United States).

Primary microglia cells were with 4% paraformaldehyde and permeabilized with 0.2% Triton X-100 in PBS. After washing, cells were blocked followed by incubation with rabbit anti-vimentin or mouse anti-paxillin at 4°C overnight. After incubation with corresponding secondary antibody, cells were washed and mounted.

For phagocytosis assay, 1 μl Alexa594-labeled latex beads (Thermo Fisher) were added to the new cell culture medium to replace the previous medium. The primary microglia cells were fixed and F-actin was visualized with Alexa 488 labeled phalloidin. The images were documented using a Nikon A1^+^ microscope system.

### Statistical Analysis

All the data were analyzed using Prism 8 Software. SPT, FST, SLAT and training day’s data in MWM were analyzed using two-way ANOVA. The rest experiments were analyzed using one-way ANOVA and Tukey correction for multiple testing between categories. *P* < 0.05 was considered as statistically significant.

## Results

### SLDS Attenuated the Depressive Symptoms in Chronic Stress Exposed Mice.

**FIGURE 1:**
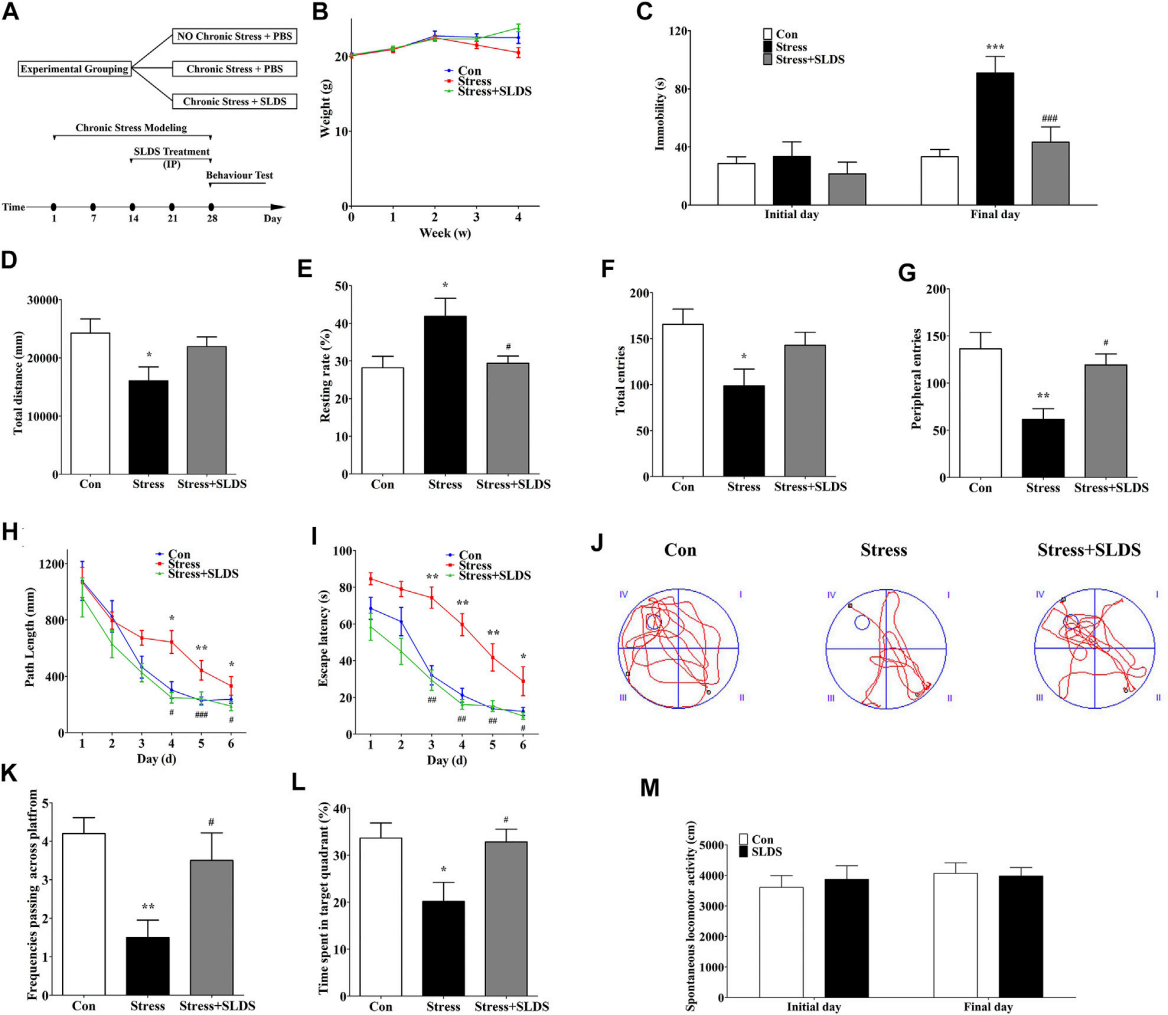
SLDS attenuated the depressive symptoms in chronic stress exposed mice. **(A)** The timeline for chronic stress induction, SLDS treatment, and behavior evaluation (FST, MWM and OFT); **(B)** The body weight measurements during four weeks chronic stress exposure; **(C)** The duration of immobility in FST (Source of variation: interaction F(2, 54) = 4.861, *P* = 0.0114, treatment factor F(2, 54) = 8.296, *P* = 0.0007, initial/final days comparison F(1, 54) = 15.67, *P* = 0.0002 ; **(D)** The total path length distance in OFT [F(2,27) = 3.752, *P* = 0.0365]; **(E)** The rate of mice motionless in OFT [F(2,27) = 4.825, *P* = 0.0158]; **(F)** The total number of entries in OFT [F(2,27) = 4.332, *P* = 0.0233]; **(G)** The number of peripheral entries in OFT [F(2,27) = 8.076, *P* = 0.0018]; **(H)** The path length of mice during the navigation test of MWM (Source of variation: interaction F(10, 208) = 1.407, *P* = 0.179, Treatment factor F(2, 208) = 13.68, *P* < 0.0001, Days factor F(5, 208) = 21.15, *P* < 0.0001); **(I)** The escape latency of mice during the navigation test of MWM (Source of variation: interaction F(10, 208) = 1.554, *P* = 0.1226, Treatment factor F(2, 208) = 56.55, *P* < 0.0001, Days factor F(5, 208) = 39.05, *P* < 0.0001); **(J)** Representative diagram in the probe trial in MWM; **(K)** The frequency of passing across the virtual platform in MWM [F(2,27) = 6.577, *P* = 0.0047]; **(L)** The time spent in target quadrant at 7^th^ day trial in MWM [F(2,27) = 5.062, *P* = 0.0136]; **(M)** The locomotor activity in SLAT (Source of variation: interaction F(1, 36) = 0.2360, *P* = 0.6301, treatment factor F(1, 36) = 0.0526, *P* = 0.8198, initial/final day comparison F(1, 36) = 0.5920, *P* = 0.4467. All data are presented as mean ± SD (*n* = 10/group). * *P* < 0.05, ** *P* <0.01, compare to control group; # *P* < 0.05 ## *P* <0.01 ###*P* < 0.005, compare to stress group.

To determine whether SLDS treatment has the effects on the depressive symptoms of chronic stress induced mice, randomly grouped mice were left untreated (control group), subjected to unpredictable stress (stress + vehicle group) or injected with SLDS during stress challenge (stress + SLDS group). After 28 days, there was no significant difference in mice body weight among three groups (Figure 1B). And then three behavioral tests, FST, MWM and OFT were performed to evaluate the therapeutic effects (Figure 1A).

In FST, the results showed that there was no statistically significant difference in immobility time among three groups before the chronic stress procedure. However, the immobility time was significantly increased in mice of the stress group after four weeks of chronic stress procedure. In contrast, the duration of immobility in stress+ SLDS group was half less than that of the stress group (Figure 1C). These results suggested that SLDS might exhibit antidepressant-like activity.

Subsequent OFT results showed that the total distance of motion was reduced in mice exposed to chronic stress for four weeks, and tendency of motionlessness was increased compared to that of the control group (*p* <0.05). While in SLDS treatment group, the movement distance and location preference of mice were comparable to that of the control group (Figures 1D–G). These results suggested that chronic stress could induce locomotor activity decrease and SLDS might ameliorate the symptoms significantly.

After OFT, the spatial learning and memory abilities of mice had been monitored with MWM. Results showed that the stress group had a significantly longer path length and increased escape latency compared to the control group during the consecutive 6 training days. However, in SLDS treated group, the path length and escape latency were significantly decreased (Figures 1H,I). On the 7th day, when probe trials were conducted to evaluate the spatial memory abilities, the stress group had a lower frequency of swimming around the original platform position. On the contrary, the stress + SLDS group showed a significantly increased swimming time in the target quadrant and increased frequency in swimming across the original platform position (Figures. 1K,L). These results indicated that SLDS treatment significantly improved the impaired spatial learning and memory caused by chronic stress.

However, for SPT there were no significant difference among all three groups (data were not shown). It might be due to the long-term water deprivation and high temperature environment which resulted in the drinking behavioral alteration. Meanwhile, to examine whether the increased activity of SLDS treated group in FST and MWM was due to a locomotor activity stimulating effect from SLDS, we conducted spontaneous locomotor activity test. The results showed no statistically difference between control group and SLDS alone group, excluding the possibility that the improved behavior in SLDS treated mice was caused by an increase in locomotor activity (Figure 1M).

### SLDS Suppressed Microglial Activation and Pro-Inflammatory Cytokines Release in Hippocampus of Chronic Stress Exposed Mice.

**FIGURE 2:**
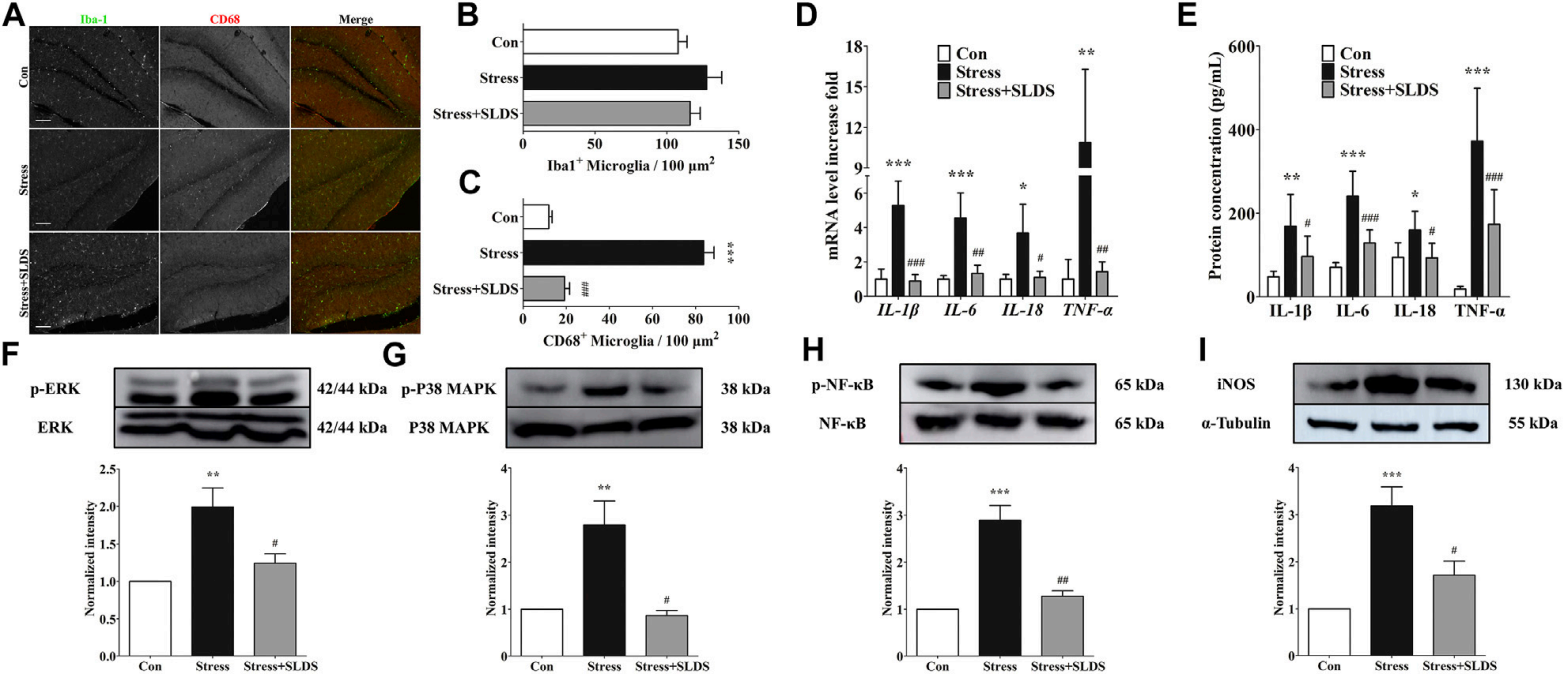
SLDS suppressed microglia activation and pro-inflammatory cytokines release in hippocampus of chronic stress exposed mice. **(A)** Representative images illustrating microglia stained with Iba-1 and CD68 antibodies in hippocampal sections. Scale bar, 100 µm; **(B)** Number of Iba-1 positive microglia in hippocampal sections [F(2,27) = 1.515, *P* = 0.2568]; **(C)** Number of CD68 positive microglia in hippocampal sections [F(2,27) = 151.9, *P* < 0.0001]; **(D)** mRNA level of IL-1β [F(2,9) = 35.23, *P* < 0.0001]*,* IL-6 [F(2,9) = 19.28, P = 0.0006]*,* IL-18 [F(2,9) = 9.481, *P* = 0.0061] and TNF-α [F(2,9) = 12.61, *P* = 0.0025] in hippocampus determined by qRT-PCR (*n* = 4); **(E)** The secretion level of IL-1β [F(2,15) = 8.063, *P* = 0.0042], IL-6 [F(2,15) = 29.19, *P* < 0.0001], IL-18 [F(2,15) = 5.890, *P* = 0.0129] and TNF-α [F(2,15) = 24.66, *P* < 0.0001] in hippocampus determined using ELISA (*n* = 4); **(F)** P-ERK1/2 [F(2,9) = 10.15, P = 0.0049] in hippocampal area tissue; **(G)** P-p38 MAPK [F(2,12) = 13.05, *P* = 0.0010] in hippocampal area tissue; **(H)** P-p65 NF-κB [F(2,15) = 28.59, P < 0.0001] in hippocampal area tissue; **(I)** iNOS expression [F(2,15) = 14.42, P = 0.0003] in hippocampal area tissue. All data are presented as mean ± SD. * *P* < 0.05, ** *P* <0.01, ***P <0.005, compare to control group; # *P* < 0.05, ## *P* <0.01, ###*P* < 0.005, compare to stress group.

In order to visualize and quantify the activated microglia in hippocampus, cryosections of hippocampus from different groups were labeled with Iba1 and CD68. There were no significant differences in the amount of Iba-1 positive microglia cells among all three groups (Figures 2A,B). However, after SLDS treatment, the amount of CD68 positive activated microglia cells was significantly reduced compared with the stress group, indicating that the SLDS suppressed microglial activation (Figures 2A,C).

Next, we investigated whether SLDS attenuated pro-inflammatory cytokines release in the hippocampus of chronic stress induced mice. Quantitative RT-PCR results revealed that the mRNA levels of IL-1β, IL-6, IL-18 and TNF-α were significantly enhanced in stress group compared with those of control (Figure 2D, *p* <0.05). On the contrary, the expression levels of these pro-inflammatory cytokines were significantly reduced after SLDS treatment. Then the expression of IL-1β, IL-6, IL-18 and TNF-α in a protein level was evaluated by using ELISA assay. Consistent with the results of qRT-PCR, the protein levels of IL-1β, IL-6, IL-18 and TNF-α were significantly enhanced in stress group compared to those of control, while SLDS treatment significantly attenuated their expression (Figure 2E, *p* <0.05).

### SLDS Inhibited Inflammatory Pathway Signaling in Hippocampus of Chronic Stress Exposed Mice.

To further determine whether SLDS attenuates inflammatory response in the chronic stress induced mice and reveal the underlying mechanism, we examined the activation of p42/p44 MAPK (ERK1/2), p38 MAPK, NF-κB and iNOS. Activation of ERK1/2, p38 MAPK and NF-κB have been highly cited in activated microglia. It is also an important pathway to activate neuroinflammation. The activation state of ERK1/2, p38 MAPK, NF-κB and the expression level of iNOS were analyzed by Western blotting. Our results showed that chronic stress increased the phosphorylation of ERK1/2, p38 MAPK, NF-κB p65 in the mice hippocampus area, which was prohibited in the SLDS treated group (Figures 2F–H). iNOS is an inducible nitric oxide synthase that is induced after brain injury and neuroinflammation. It also has been reported as the downstream product of the p38 MAPK signaling pathway (Yoshida et al., 2020). In this study, the upregulated iNOS expression induced by stress challenge was also attenuated by SLDS (Figure 2I). These data indicated that inflammation-related signaling pathways were most likely inhibited by SLDS treatment after exposure to chronic stress.

### SLDS Reduced the Levels of Proinflammatory Cytokines in LPS Induced Primary Microglia.

**FIGURE 3:**
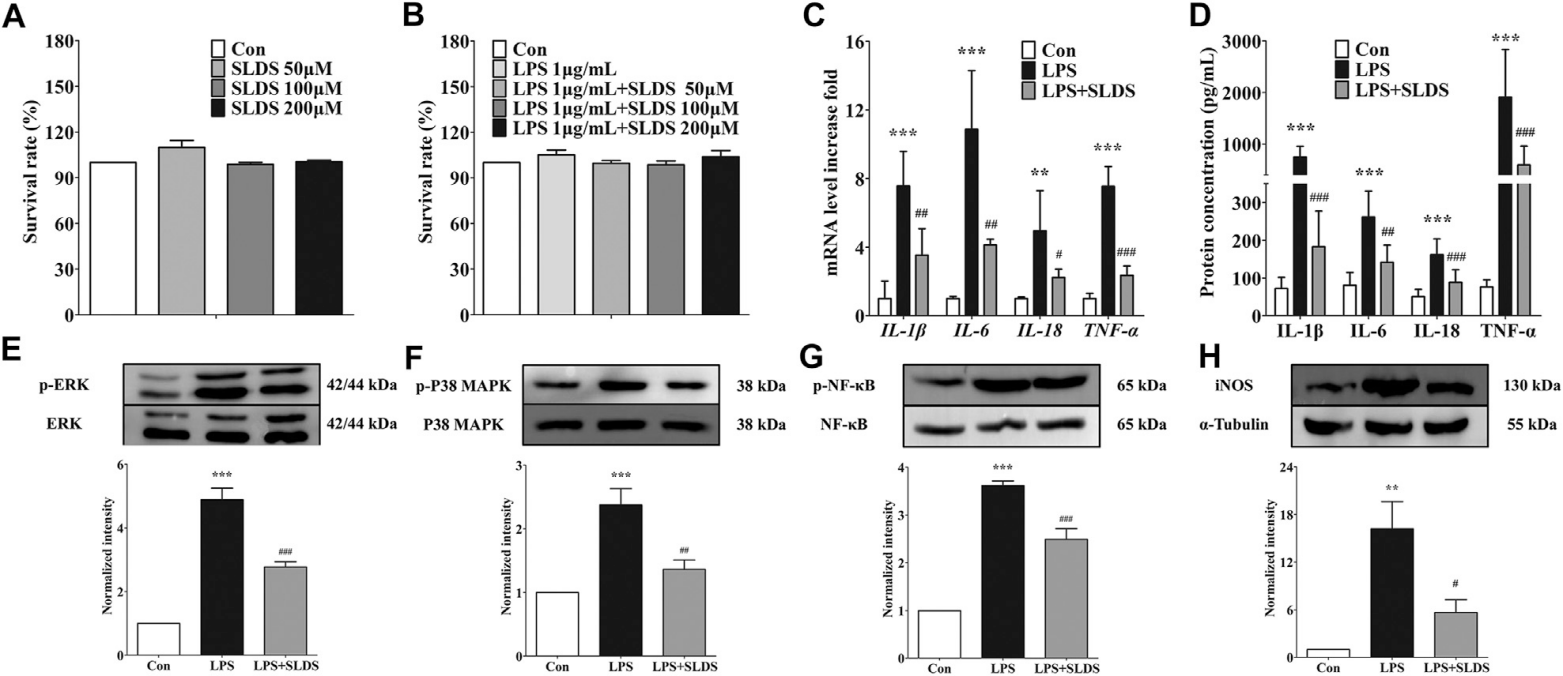
SLDS reduced the levels of proinflammatory cytokines and suppressed microglial activation in LPS induced primary microglia. **(A)** The cell viability with SLDS (0, 50, 100 and 200 μM) alone [F(3,12) = 1.524, *P* = 0.2588] in primary microglia; **(B)** The cell viability with SLDS (0, 50, 100 and 200 μM) and LPS 1 μg/ml [F(4,31) = 0.6963, *P* = 0.6003] in primary microglia; **(C)** mRNA levels of IL-1β [F(2,9) = 17.54, *P*= 0.0008]*, I*L-6 [F(2,9) = 25.80, *P* = 0.0002]*,* IL-18 [F(2,9) = 8.516, *P* = 0.0084] and TNF-α [F(2,9) = 81.80, *P* < 0.0001] in primary microglia (*n* = 4); **(D)** The secretion levels of IL-1β [F(2,21) = 57.51, *P* = < 0.0001], IL-6 [F(2,21) = 19.17, *P* < 0.0001], IL-18 [F(2,21) = 21.64, *P* < 0.0001] and TNF-α [F(2,21) = 23.71, *P* < 0.0001] in primary microglia (*n* = 4); *(E)* P-ERK1/2 [F(2,15) = 72.91, *P* < 0.0001] in primary microglia; **(F)** P-p38 MAPK [F(2,12) = 17.38, *P* = 0.0003] in primary microglia; **(G)** P-p65 NF-κB [F(2,9) = 86.64, *P* < 0.0001] in primary microglia; **(H)** iNOS expression [F(2,11) = 12.46, *P* = 0.0015] in primary microglia. All data are presented as mean ± SD. ** *P* <0.01, ***P <0.005, compare to control group; # *P* < 0.05 ## *P* <0.01 ###*P* < 0.005, compare to LPS treated group.

In order to further examine whether SLDS has the same beneficial effects towards LPS insulting *in vitro*, primary microglia was cultured and stimulated by LPS. The cytotoxicity of SLDS on primary microglia were firstly evaluated. Different concentrations (0, 50, 100 and 200 μM) of SLDS with or without LPS 1 μg/ml were used to treat primary microglia and the survival rate of cells was determined. It showed that SLDS had no cytotoxicity in primary microglia at settled concentrations (Figures 3A,B). Then the effects of a relatively low concentration of SLDS on the expression and release of proinflammatory cytokines in LPS induced inflammatory response were investigated. qRT-PCR results showed that SLDS significantly reduced mRNA expression of IL-1β, IL-6, IL-18 and TNF-α (Figure 3C, *p* <0.05). ELISA results also revealed a reduction in secreted IL-1β, IL-6, IL-18 and TNF-α upon SLDS treatment (Figure 3D). It could be concluded that SLDS exhibited anti-proinflammatory effects on LPS induces inflammatory responses in primary microglia.

### SLDS Decreased LPS Induced Inflammation Through Prohibiting the Same Pathway in Primary Microglia.

Previous studies revealed p38 MAPK, ERK1/2 and NF-κB signaling is an important pathway in the transcriptional regulation of proinflammatory cytokines (Xiao et al., 2002; Dong et al., 2014), and iNOS involves in nitric oxide secretion. Given the beneficial effects of SLDS on LPS induced inflammatory response of primary microglia, it was wondered whether SLDS could prohibit the inflammatory signaling pathway in primary microglia as same as the brain tissue sample. In this study, Western blotting assay for phosphorylation of ERK1/2, p38 MAPK, NF-κB and expression level of iNOS had been performed. The results revealed that SLDS significantly attenuated activation of ERK1/2, p38 MAPK, NF-κB and expression level of iNOS in LPS-stimulated primary microglia (Figures 3E–G), which were consistent with the results that were found in chronic stress exposed mice.

### SLDS Inhibits Microglial Morphological Alterations and Phagocytic Ability in LPS Induced Primary Microglia.

**FIGURE 4:**
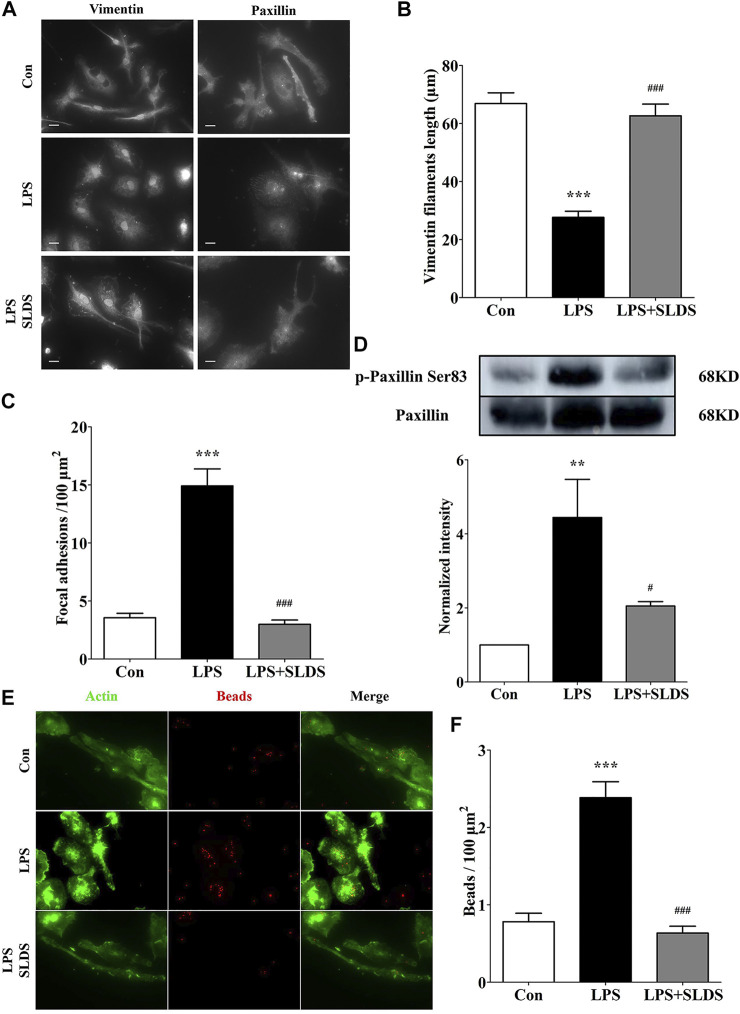
SLDS inhibited microglial morphological alterations and phagocytic ability in LPS induced primary microglia. **(A)** Representative images illustrating primary microglia stained with vimentin and paxillin antibodies, scale bar: 10 μm; **(B)** The length of vimentin filaments in primary microglia [F(2,60) = 39.11, *P* < 0.0001]; **(C)** The number of focal adhesions in primary microglia [F(2,36) = 55.93, *P* < 0.0001]; **(D)** P-paxillin at Ser83 [F(2,9) = 8.705, *P* = 0.0079] in primary microglia; **(E)** Representative images illustrating microglial phagocytosis; **(F)** The number of beads engulfed by primary microglia [F(2,59) = 35.24, *P* < 0.0001]. All data are presented as mean ± SD. ** *P* <0.01, ***P <0.005, compare to control group; # *P* < 0.05, ###*P* < 0.005, compare to LPS treated group.

In order to determine morphological alterations in microglial cells upon LPS challenging, the length of vimentin filaments was determined, which were in parallel to the longitudinal axis of primary microglia cells. The results showed that the length of vimentin filaments was significantly shortened in LPS induced primary microglia, which resulted in the changes in cell shape and size (Figures 4A,B). However, SLDS significantly reduced the loss of vimentin filaments (Figures 4A,B, *p*<0.05).

We further wondered whether alterations in cell shape and size of microglia affected assembly of focal adhesions. The focal adhesion was examined using the paxillin, which was a component of focal adhesions in primary microglia. The amount of focal adhesions was significantly enhanced in LPS induced primary microglia, and in contrast, SLDS restored focal adhesion numbers comparable to a condition of the resting primary microglia, which was consistent with its inhibition of cell spreading (Figures 4A,C). Western blotting analysis showed SLDS suppressed the enhanced phosphorylation at Ser83 in paxillin induced by LPS in primary microglia (Figure 4D).

The morphological changes and cell adhesion of microglia cells might eventually affect their phagocytic activity. Therefore, we further examined whether the phagocytic ability of primary microglia was affected by SLDS after exposed to LPS by using fluorescent beads. Phagocytosis assay showed primary microglia exhibited stronger phagocytic ability after LPS stimulation, while SLDS could significantly diminish LPS induced phagocytic activity, which was consistent with morphology and adhesion analysis results (Figures 4E,F).

### SLDS Prevented Neuronal Apoptosis in Chronic Stress Exposed Mice and Decreased Microglial Neurotoxicity in PC12 Cells.

**FIGURE 5:**
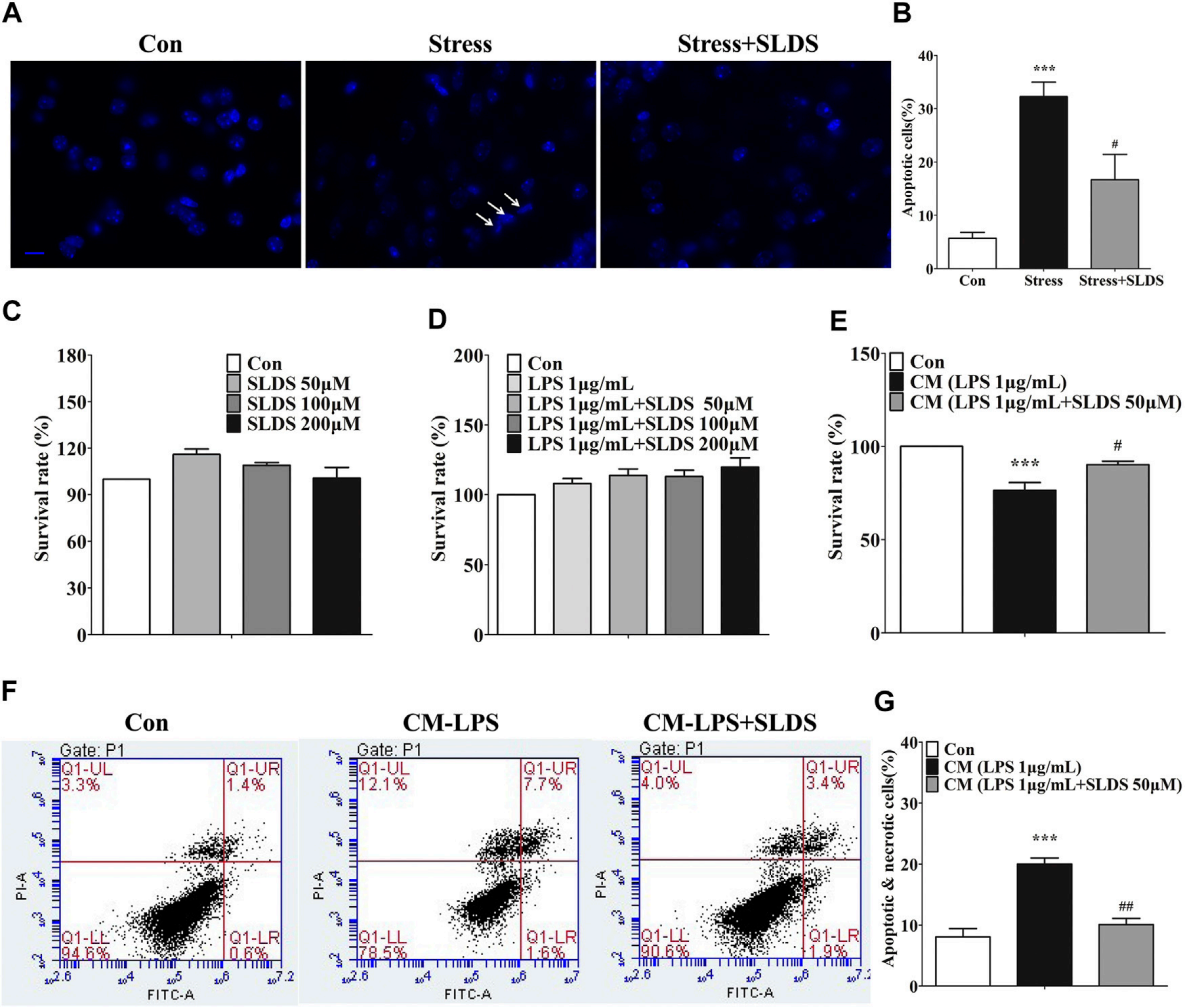
SLDS prevented neuronal apoptosis in chronic stress exposed mice and decreased microglial neurotoxicity in PC12 cells. **(A)** Representative images illustrating apoptotic cells in hippocampal sections, scale bar: 10 μm; **(B)** Quantification of apoptotic cells in the hippocampus of mice [F(2,13) = 18.20, *P* = 0.0002]. All data are presented as mean ± SD. ***P <0.005, compare to control group; # *P* <0.05, compare to stress group. **(C)** The cell viability for PC12 cells treated with SLDS (0, 50, 100 and 200 μM) alone [F(3,8) = 3.611, *P* = 0.0650]; **(D)** The cell viability for PC12 cells treated with SLDS (0, 50, 100 and 200 μM) and LPS 1 μg/ml [F(4,10) = 2.766, *P* = 0.0874]; **(E)** The cell viability for PC12 cells treated with CM [F(2,13) = 18.20, *P* = 0.0002]; **(F)** Representative images illustrating the flow cytometry analysis for PC12 cells; **(G)** The percentage of apoptotic and necrotic PC12 cells assessed using flow cytometry analysis [F(2,9) = 19.79, *P* = 0.0005]. All data are presented as mean ± SD. ***P <0.005, compare to control group; ## *P* <0.01, ###*P* < 0.005, compare to CM-LPS group.

Microglial activation induced by chronic stress causes neuronal apoptosis. In apoptotic cells, compaction, condensation and segregation of the nuclear chromatin appear. We used Dapi staining to determine whether SLDS could reduce neuronal apoptosis. The immunofluorescence microscopy assay revealed that SLDS could distinctly reduce the amount of apoptotic nucleus in hippocampus of chronic stress exposed mice (Figures 5A,B). To further conform this effect of SLDS, we cultured PC12 cells with CM derived from primary microglia cultures to detect the microglial neurotoxicity. A CCK-8 kit was used to detect whether SLDS exhibited cytotoxicity to PC12 cells. The results showed that SLDS exhibited no cytotoxicity in PC12 cells at the detected concentrations (Figures 5C,D). It showed that the viability of PC12 cells was significantly increased in CM from primary microglia cultures with SLDS pretreatment (Figure 5E). Furthermore, flow cytometry assay with Annexin V/PI staining was used to evaluate neuroprotective properties of SLDS. Results showed that CM from primary microglia cultures pretreated with SLDS significantly reduced apoptosis in PC12 cells (Figures 5F,G). In conclusion, SLDS might achieve neuroprotective functions by inhibiting microglial activation.

## Discussion

**FIGURE 6:**
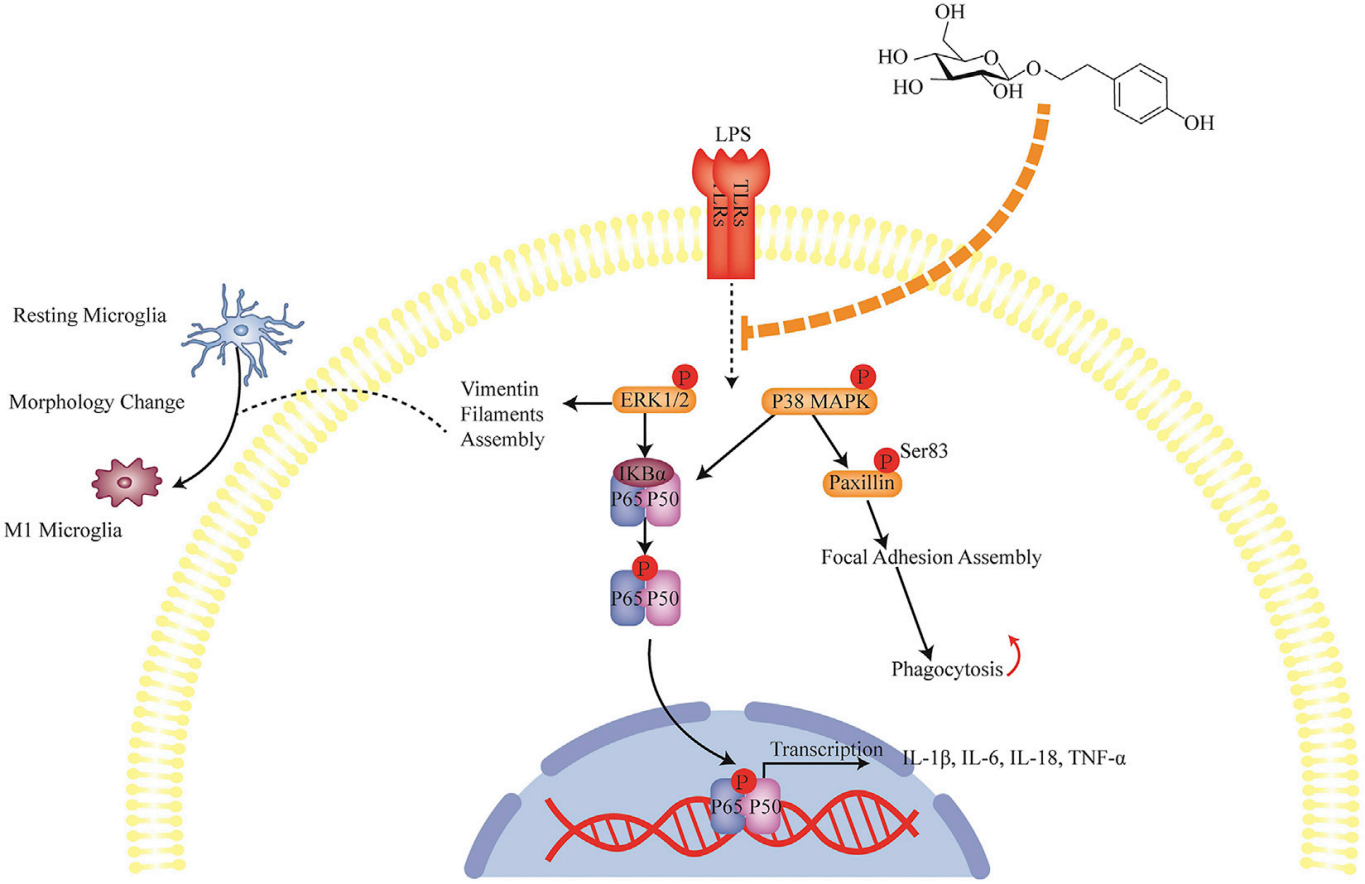
Anti-inflammation mechanism of SLDS in LPS induced primary microglia. LPS signal from infections activates microglia through TLRs and further activates ERK1/2, p38 MAPK, NF-κB signaling. Signals translocate to nucleus and induce the transcription of inflammatory cytokines IL-1β, IL-6, IL-18 and TNF-α. On the other hand, cytoskeleton changes generate the mechanical forces that drive cell motility and phagocytosis which are closely associated with microglial function. LPS signals induce the cytoskeleton protein vimentin filaments assembly which leads to the morphological changes from ramified state to ameboid state. Meanwhile, they regulate focal adhesion assembly which results in the cell attachment state alteration. SLDS can inhibit the LPS signals transduction thus blocking the activation of microglia and prevent cytoskeleton changes.

In this study, we illustrated that SLDS treatment relieved the depressive symptoms induced via unpredictable chronic stress in mice and reduced the amount of deleterious activated microglia (CD68 positive) in hippocampus. It was found that SLDS treatment inactivated ERK1/2, p38 MAPK, NF-κB signaling, suppressed iNOS expression, and caused impaired phagocytosis and reduced the expression and release of pro-inflammatory cytokines (Figure 6). These findings underpinned a possible molecular mechanism that underlies the beneficial effects of SLDS in relieving the behavioral and cognitional disorders in a depressive mouse model, and therefore lying a foundation for the therapeutic opportunity of SLDS in treating depression.

Unpredictable chronic stresses have been reported as a promising method to cause a series of depressive behaviors in rodents (Farooq et al., 2012; Nollet et al., 2013; Willner 2017). In the present study, the depressive mouse model was generated using this method and depressive symptoms were assessed by using FST, MWM and OFT. For studies using all kinds of depressive animal models, FST is still one of the most commonly used assay for screening antidepressants in preclinical studies (Petit-Demouliere et al., 2005). MWM has been well defined to examine spatial memory and cognition, while OFT has been widely used to evaluate locomotivity and anxiety levels (Jin et al., 2019; Zhang N., et al., 2020). Consistent with previous researches, our results confirmed that the exposure to unpredictable chronic stress leads to a significant memory loss and volitional activity decline (Gouirand and Matuszewich 2005; Hu et al., 2015; Trofimiuk and Braszko 2015; Li et al., 2017). In recent years, studies have revealed that during pathological process, microglia plays an important role in the destruction of neural plasticity and has deleterious effects on neuroprotection, leading to neuroinflammation and aggravation of depression (Singhal and Baune 2017). Moreover, many drugs fulfill their antidepressant function through regulating the activation of microglia and anti-neuroinflammation in preclinical studies (Han et al., 2019; Feng et al., 2020; Vega-Rivera et al., 2020). These findings emphasized the importance of modulating microglia activity for interfering nervous system disorders.

Salidroside, a phenolic glycosides compound extracted from *Rhodiola rosea*, has been reported in possession of neuroprotective properties (Liu et al., 2018; Wang C., et al., 2018). Researchers also showed that SLDS could suppress inflammatory cytokines release and improved depressive symptoms (Vasileva et al., 2018). However, the underlying molecular mechanism remained unclear. In the pathological process, microglia participates in the immune response by migrating to the pathological area and activating itself to fulfill its functions (Domercq et al., 2013). In this study, it was found that SLDS treatment shared a molecular mechanism similar to many other drugs that regulates microglia activation and anti-neuroinflammation. In addition, we also proposed another molecular mechanism in which SLDS might promote anti-depression by altering the morphology and attachment state of microglia and therefore avoid their enrichment in pathological areas.

Microglia, a type of glial cell that is equivalent to macrophages in the brain and spinal cord, is the first and most important immune defense of the CNS. They normally exist in a resting ramified state and become activated in response to different stimuli. Microglia is of great importance in immune defender in the brain by eliminating apoptotic debris and pathogen (Beyer et al., 2000; Gogoleva et al., 2019). Stimulation of microglia by LPS or colony-stimulating factor (CSF) results in an increased expression of CD68, a lysosomal protein, representing a hallmark for M1 phenotype microglia (Lei et al., 2020). Up-regulated CD68 expression in microglia has also been observed in various kinds of neurological diseases, such as Parkinson’s disease, Alzheimer’s disease, Pelizaeus-Merzbacher disease and brain tumor, which involves in neurodegeneration, neuronal death and neuroinflammation (Aronica et al., 2005; Bachstetter et al., 2013; Bachstetter et al., 2015; Aono et al., 2017). Indeed, the elevated amount of CD68 positive microglia in hippocampus has been observed after chronic stress exposure, indicative of a possible proinflammatory response. We observed that SLDS exhibited strong anti-inflammatory effects by decreasing the CD68 positive microglia cells in hippocampus after chronic stress exposure in mice.

Activated microglia (M1 phenotype) can produce a wide scope of proinflammatory cytokines, such as IL-1β, IL-6, IL-18, TNF-α and other mediators, which eventually induce neuronal damage (Jin et al., 2019; Lajqi et al., 2020). Assessed using ELISA assays and qRT-PCR, the expression levels of cytokines IL-1β, IL-6, IL-18, and TNF-α in hippocampus tissue after chronic stress are increased in a manner similar to that in LPS induced primary microglia, suggesting a robust immune response induced in both model systems. SLDS suppressed the levels of IL-1β, IL-6, IL-18 and TNF-α both *in vitro* and *in vivo*. Interestingly, toxicological tests showed that LPS did not inhibit the proliferation of primary microglia as it does in BV_2_ microglia (Lee et al., 2012), suggesting possible physiological differences between BV_2_ microglia cells and primary microglia (Fukushima et al., 2015). In addition, the aged microglia cells also showed differences in response to the chemicals, indicating effects of differentiation status of the cells (Perry et al., 1993). In this study, the results newly uncovered that SLDS might be a potential natural product to inhibit microglial activation and neuroinflammation.

SLDS exhibits its anti-neuroinflammation properties by altering many aspects of microglial activation, such as changes in microglial morphology and the secretion of inflammatory cytokines. In our efforts to illustrate the molecular mechanism of SLDS anti-neuroinflammation effects, it was found that SLDS inhibited the phosphorylation of ERK1/2, p38 MAPK, NF-κB p65 and expression level of iNOS in both chronic stress exposed mice and LPS induced primary microglia. Modulation of NF-κB p65 activity by SLDS has been widely reported (Hu H et al., 2014; Wang et al., 2017). Our findings were consistent with previous results that inactivation of ERK1/2, NF-κB p65 and PI3K/Akt by SLDS, led to the reduction of iNOS and Cyclooxygenase-2 (COX-2) expression (Hu H et al., 2014; Wang et al., 2017; Wang, J et al., 2018). Activation of p38 MAPK has been revealed to regulate microglia morphological changes and phagocytic ability (Fan et al., 2018; Yao and Fu 2020). Therefore, we investigated the effect of SLDS on microglial morphological alterations and phagocytic ability. It was observed that the length of vimentin filaments was significantly shortened in LPS induced primary microglia, consistent with many other studies on activated microglial morphology. In activated microglia, cells become more flattened and loss branches (Kloss et al., 2001; Tynan et al., 2010). Focal adhesions assembling involves in cell attachment and phagocytic ability. With paxillin, a component of focal adhesion as the marker, SLDS was found to disassemble the focal adhesion which is assembled after LPS stimulation, indicating the blockage of microglia spreading. Phagocytic activity has been reported to be regulated by paxillin and cofilin (Gitik et al., 2014). SLDS can significantly attenuated LPS induce phagocytic activity. In this study, chronic stress model for depressive symptoms shares similar properties as LPS induced primary microglia. And a certain number of studies also revealed both chronic stress and LPS challenges induced distinct molecular and behavioral changes in mice (Espinosa-Oliva et al., 2011; Couch et al., 2016). For instance, Toll-like receptors (TLRs) play an important role in this depressive behavioral changes (Habib et al., 2015; Zhang.W., et al., 2020). Further studies are needed to address whether antidepressant effects of SLDS also affects TLRs pathway. In conclusion, our data demonstrated that SLDS could prevent the deleterious M1 microglial activation, and improve the depressive symptoms caused by unpredictable chronic stress. This study suggested a potential application of SLDS in therapeutic treatment of depression.

## Data Availability

The raw data supporting the conclusions of this article will be made available by the authors, without undue reservation.
