# Supplementary figures and images for: Salidroside Improves Chronic Stress Induced Depressive Symptoms Through Microglial Activation Suppression
Source: Front Pharmacol. 2021 Jun 8;12:635762. doi: 10.3389/fphar.2021.635762 (PMC8217647; doi:10.3389/fphar.2021.635762)

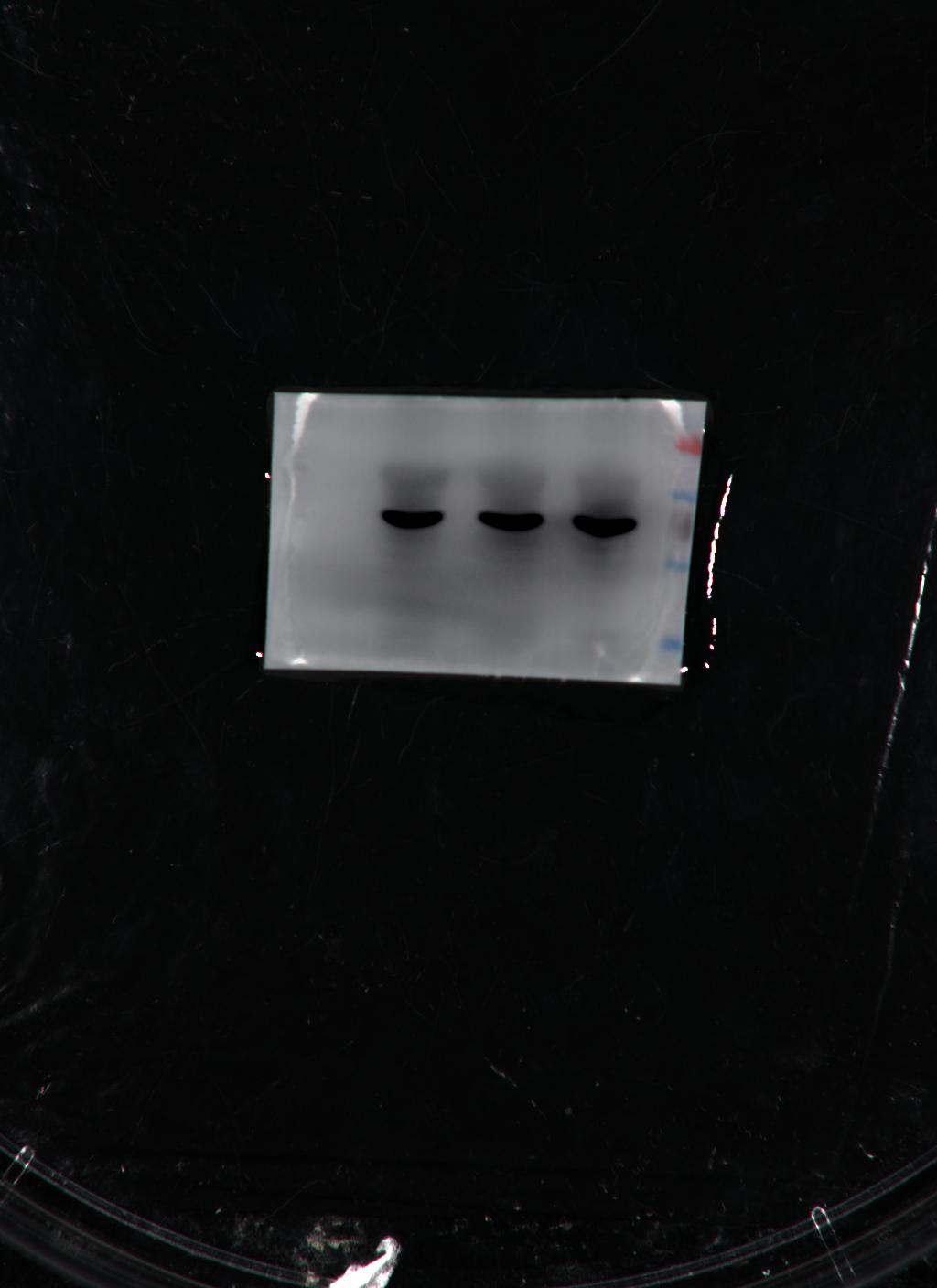

Supplement: Supplementary file 1 [file DataSheet1.zip › animal samples-WB supplementary materials/hippo-iNOS/hippo-b-tubulin 1/hippo-b-tubulin+Marker.jpg]

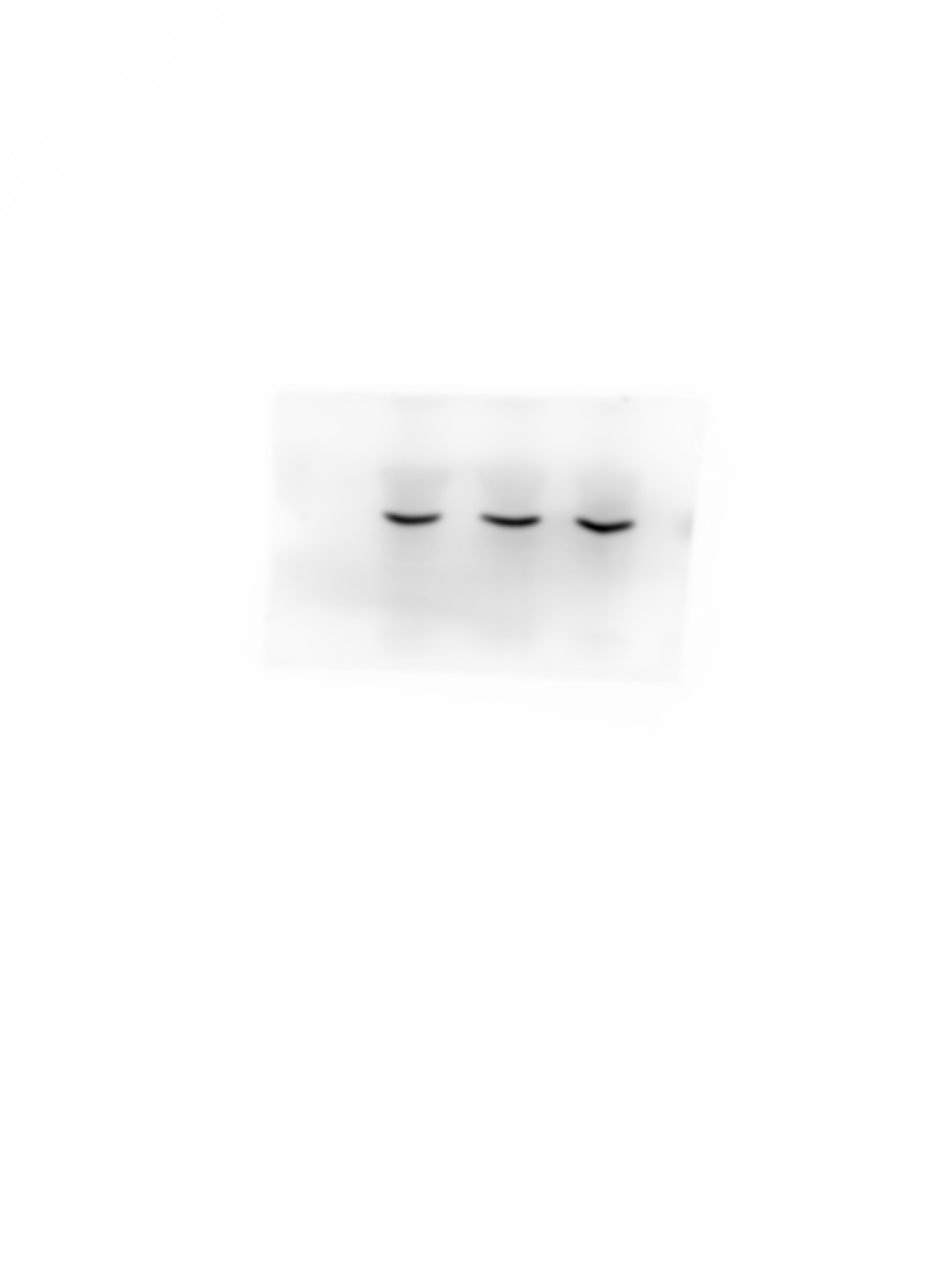

Supplement: Supplementary file 1 [file DataSheet1.zip › animal samples-WB supplementary materials/hippo-iNOS/hippo-b-tubulin 1/hippo-b-tubulin.tif]

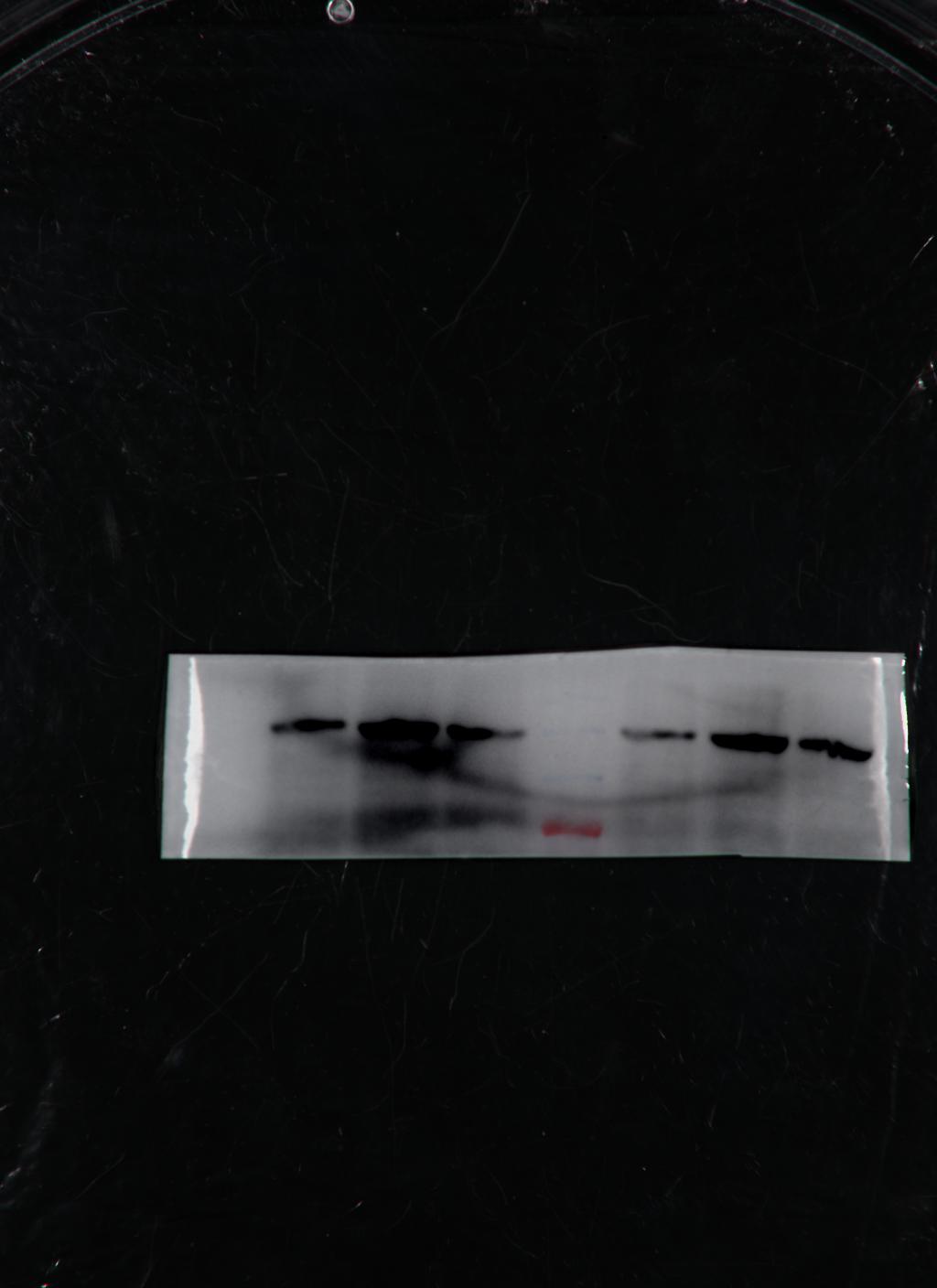

Supplement: Supplementary file 1 [file DataSheet1.zip › animal samples-WB supplementary materials/hippo-iNOS/hippo-inos 1/hippo-inos+Marker.jpg]

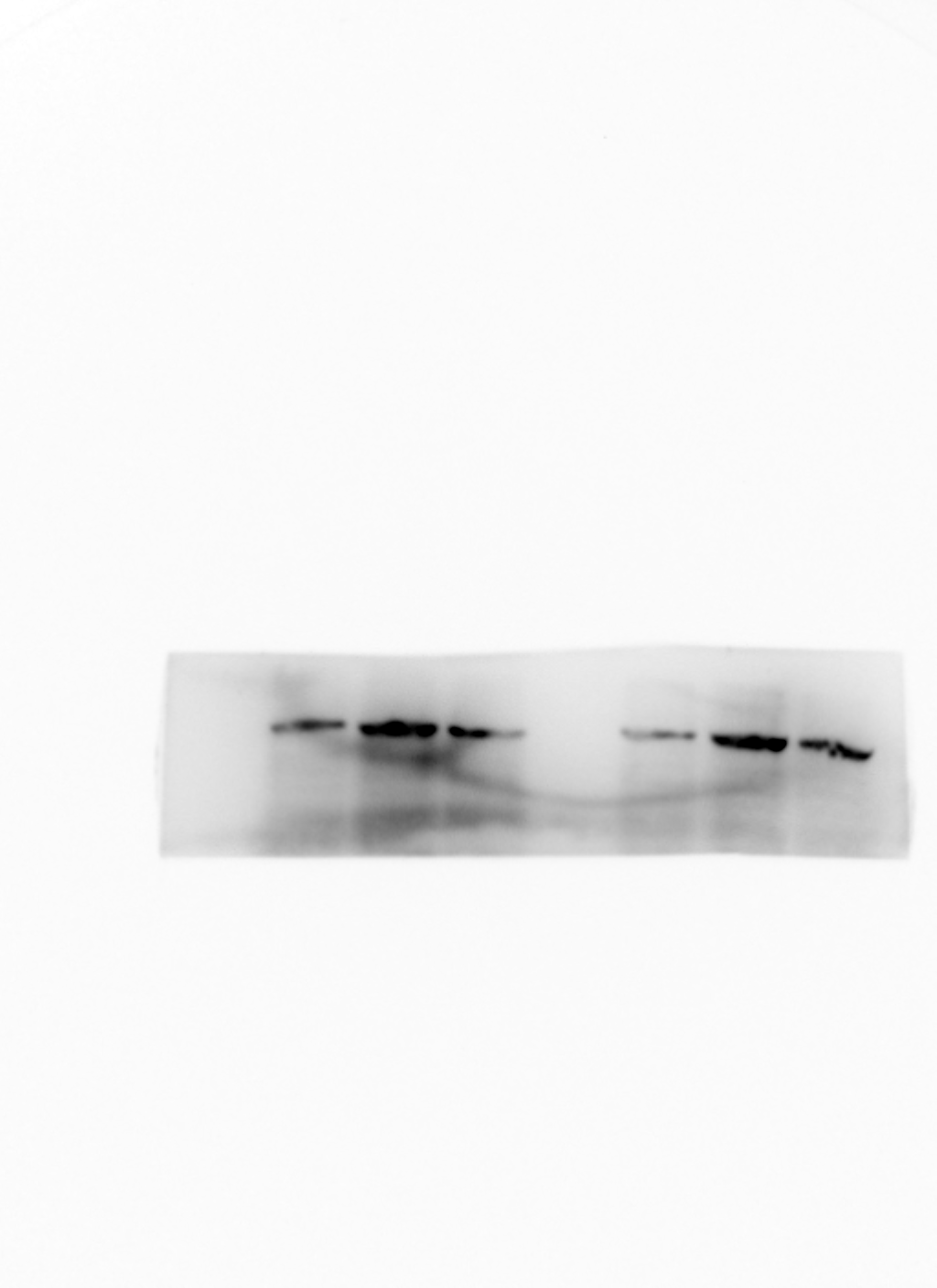

Supplement: Supplementary file 1 [file DataSheet1.zip › animal samples-WB supplementary materials/hippo-iNOS/hippo-inos 1/hippo-inos.tif]

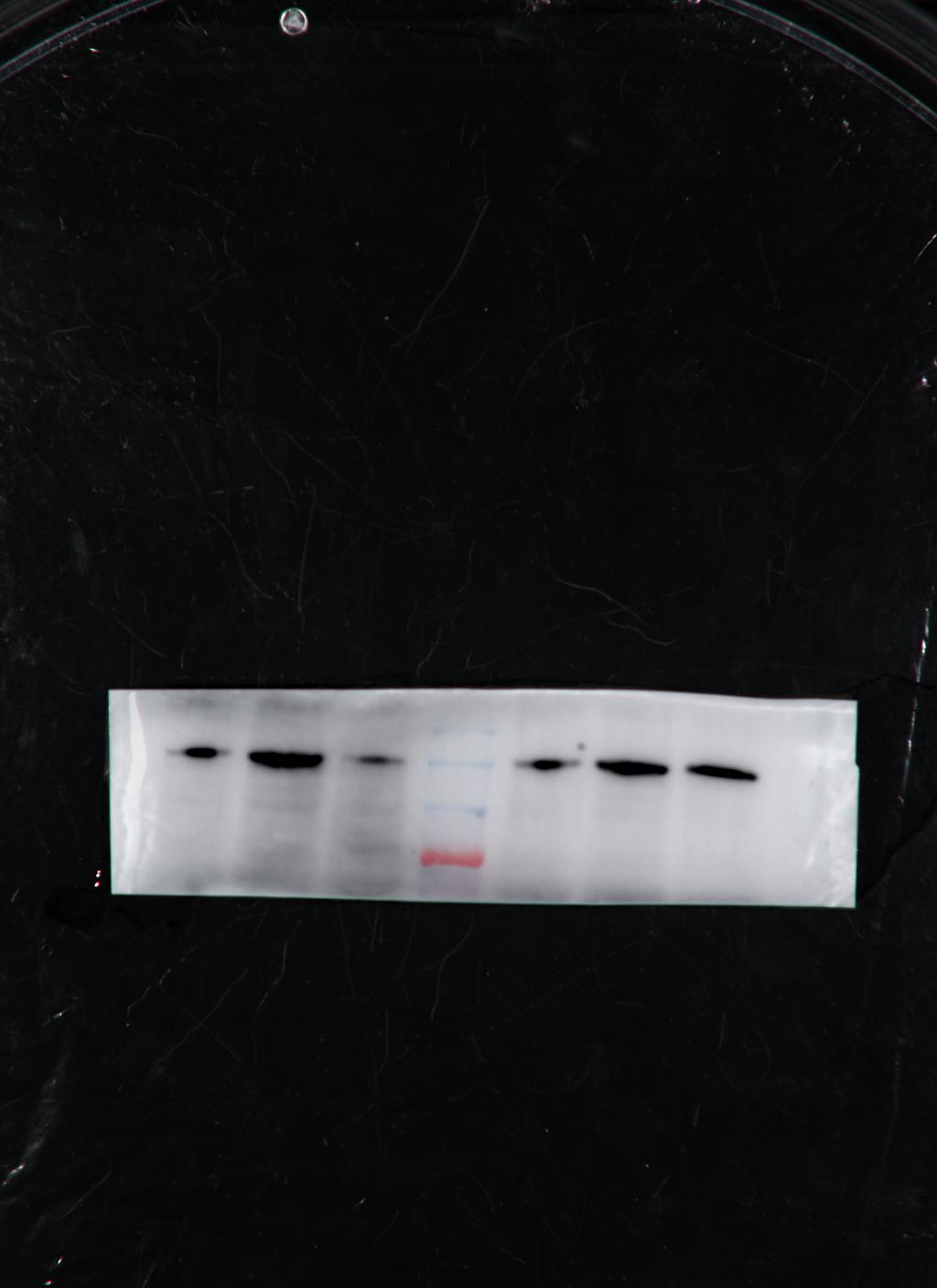

Supplement: Supplementary file 1 [file DataSheet1.zip › animal samples-WB supplementary materials/hippo-iNOS/hippo-inos 2/hippo-inos+Marker.jpg]

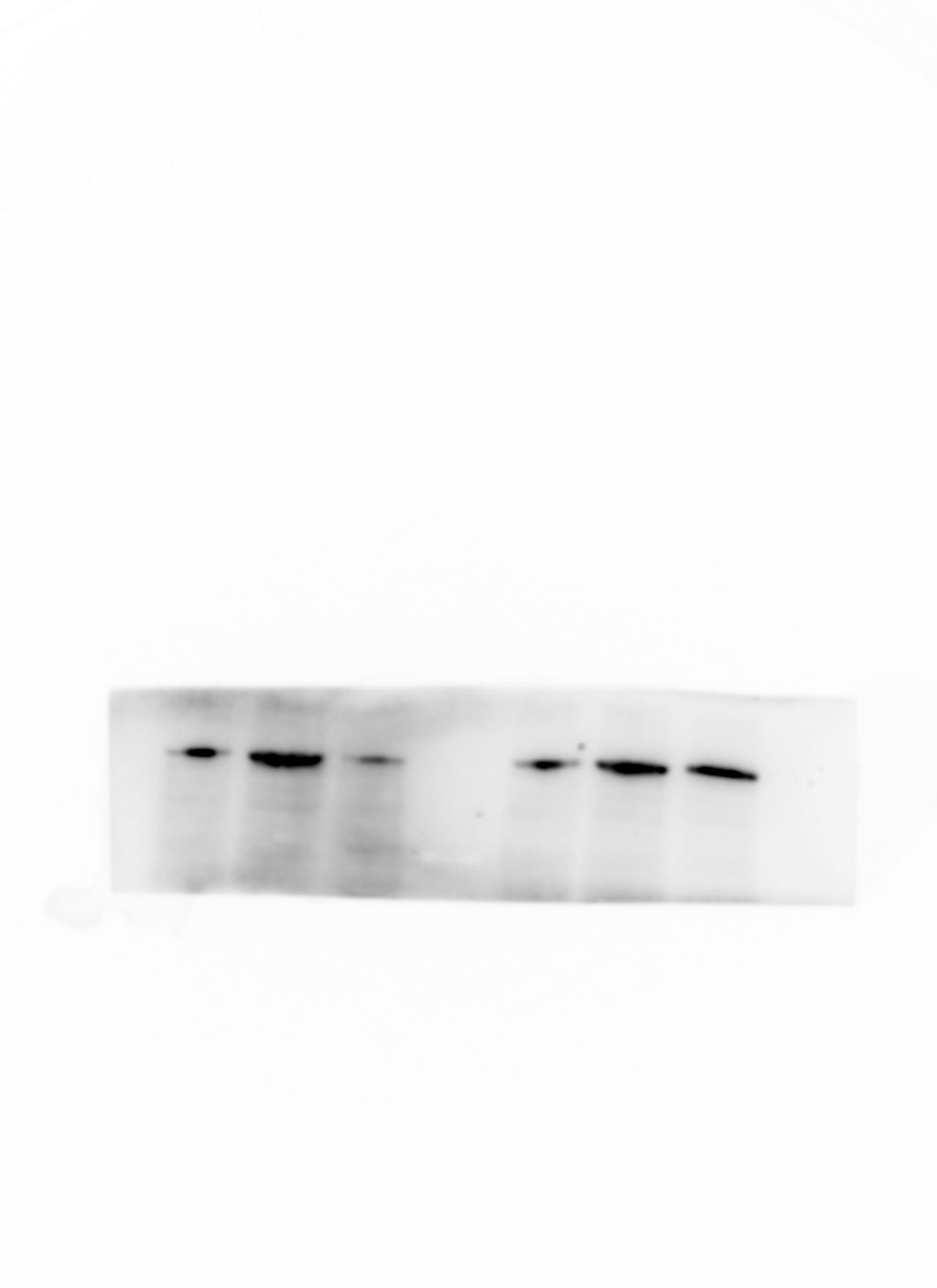

Supplement: Supplementary file 1 [file DataSheet1.zip › animal samples-WB supplementary materials/hippo-iNOS/hippo-inos 2/hippo-inos.tif]

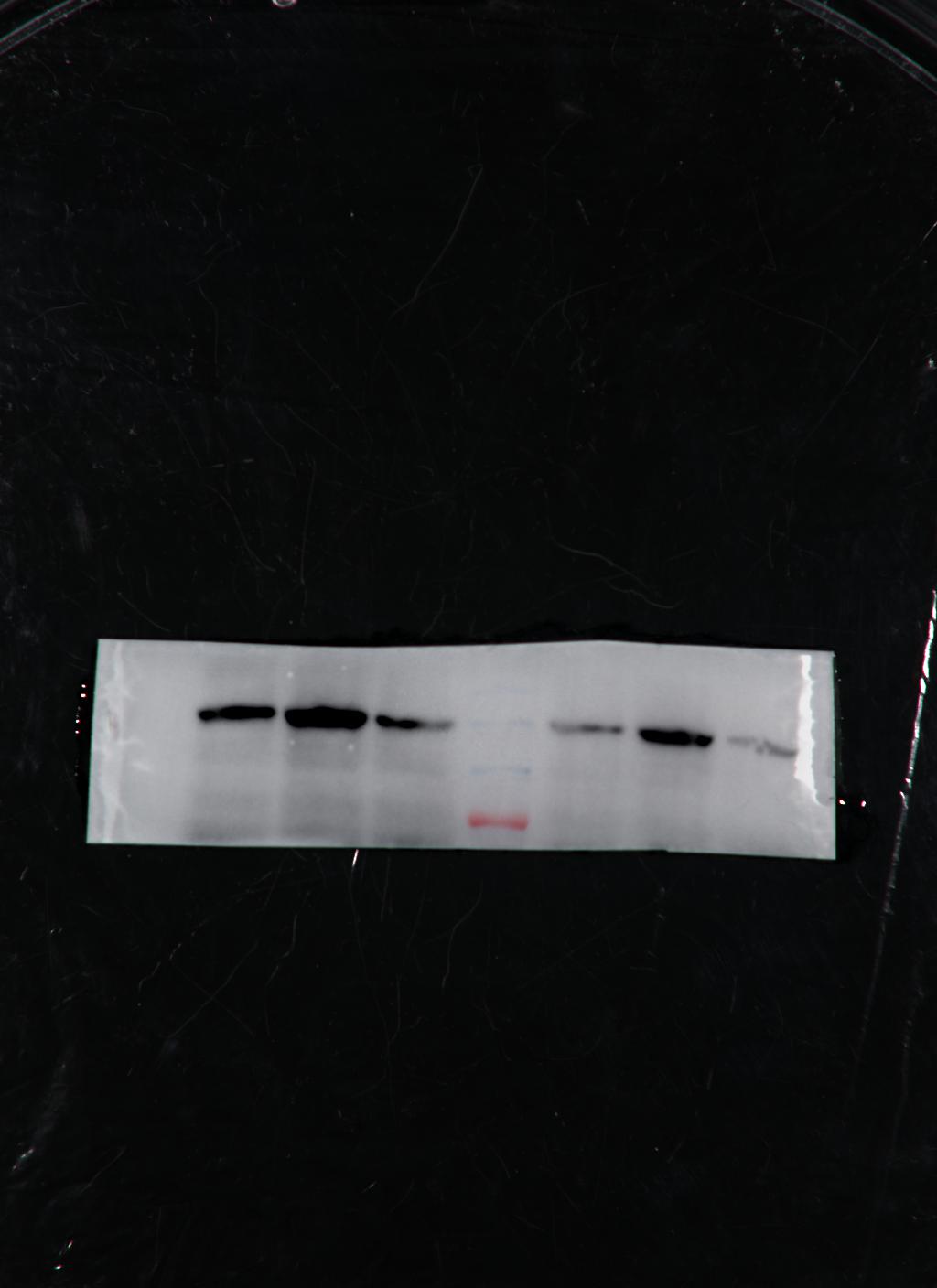

Supplement: Supplementary file 1 [file DataSheet1.zip › animal samples-WB supplementary materials/hippo-iNOS/hippo-inos 3/hippo-inos +Marker.jpg]

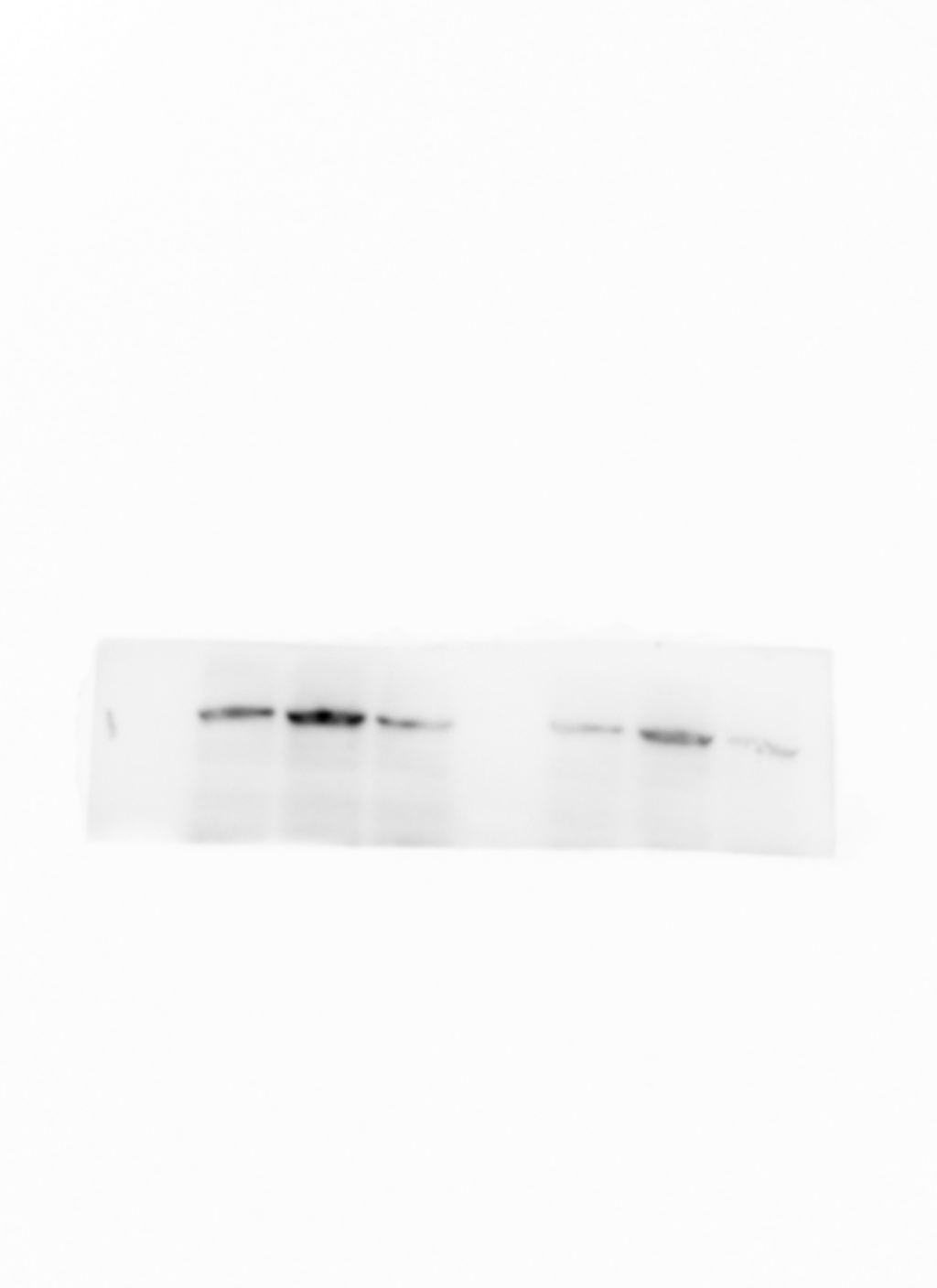

Supplement: Supplementary file 1 [file DataSheet1.zip › animal samples-WB supplementary materials/hippo-iNOS/hippo-inos 3/hippo-inos .tif]

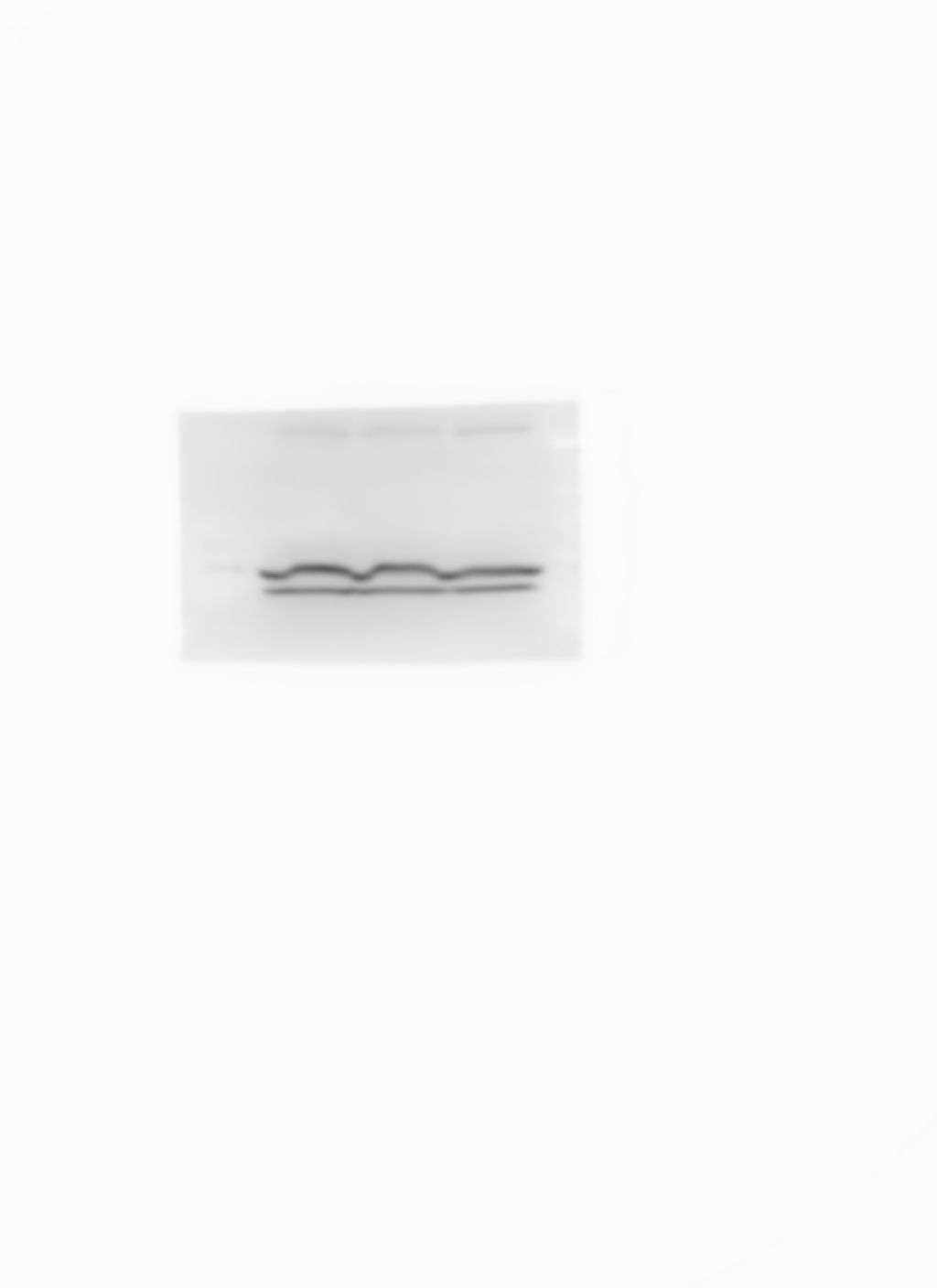

Supplement: Supplementary file 1 [file DataSheet1.zip › animal samples-WB supplementary materials/hippo-p-erk/hippo erk 1/hippo erk .tif]

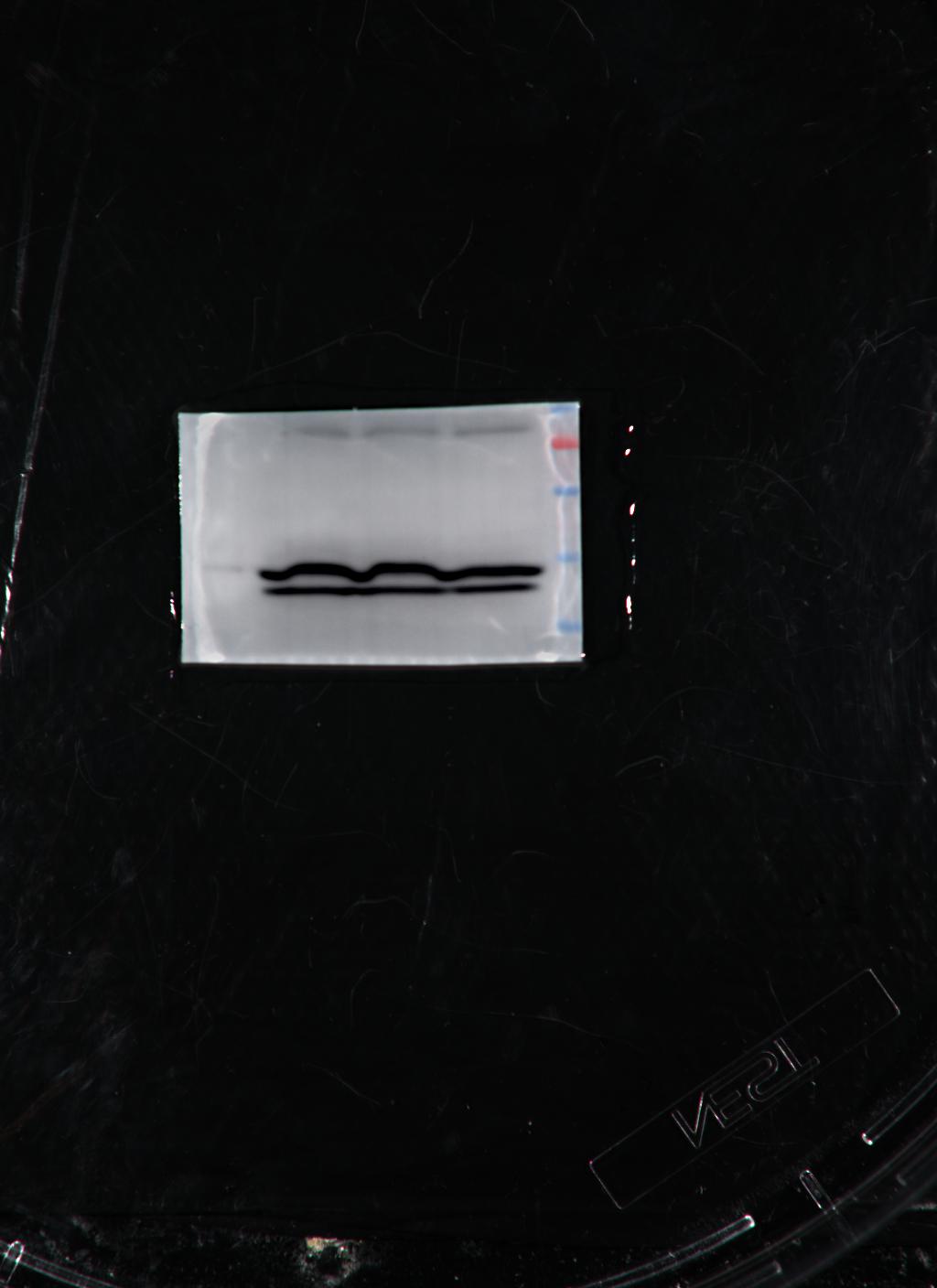

Supplement: Supplementary file 1 [file DataSheet1.zip › animal samples-WB supplementary materials/hippo-p-erk/hippo erk 1/hippo erk+Marker.jpg]

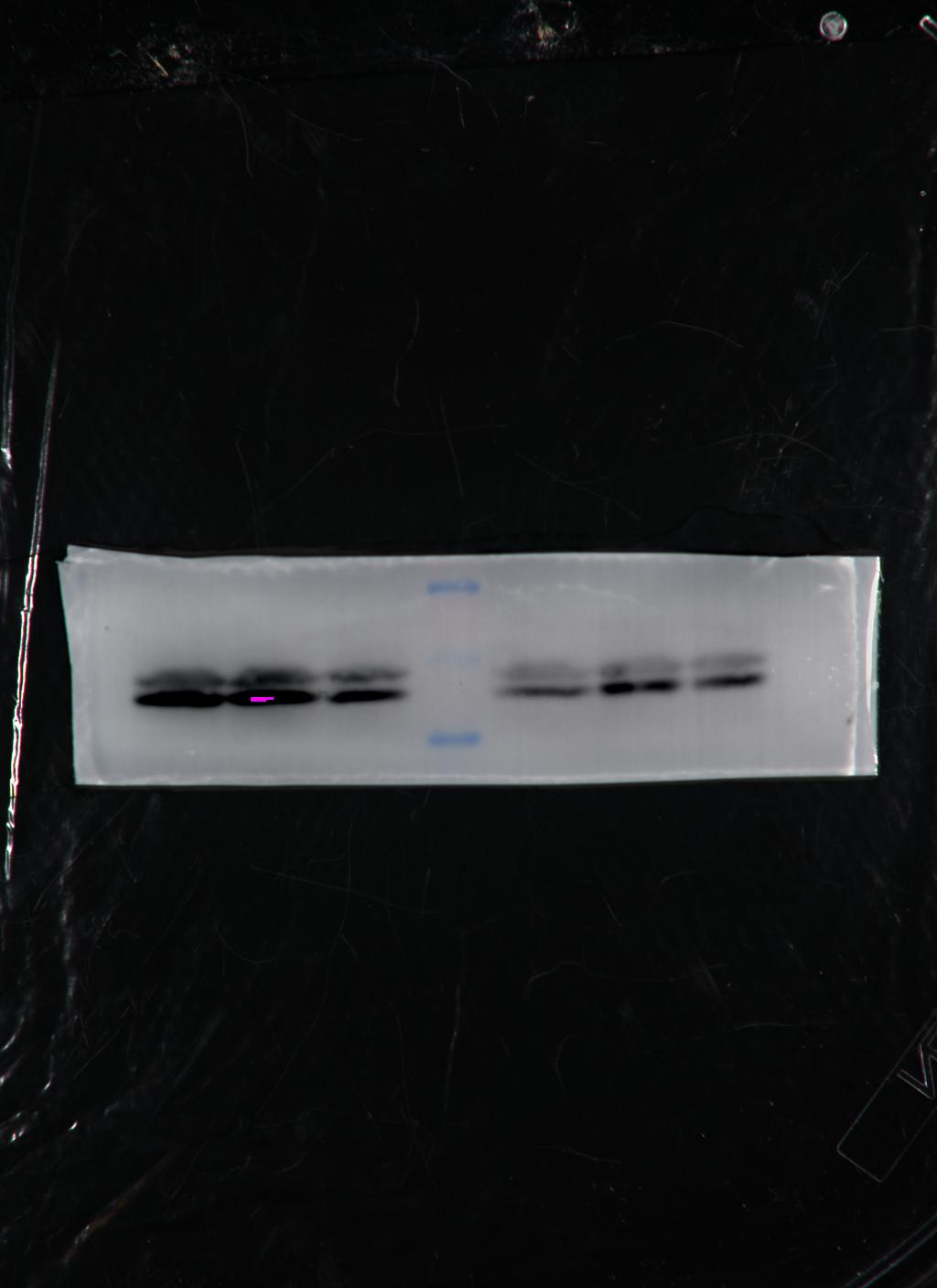

Supplement: Supplementary file 1 [file DataSheet1.zip › animal samples-WB supplementary materials/hippo-p-erk/hippo p-ERK 1/hippo p-ERK+Marker.jpg]

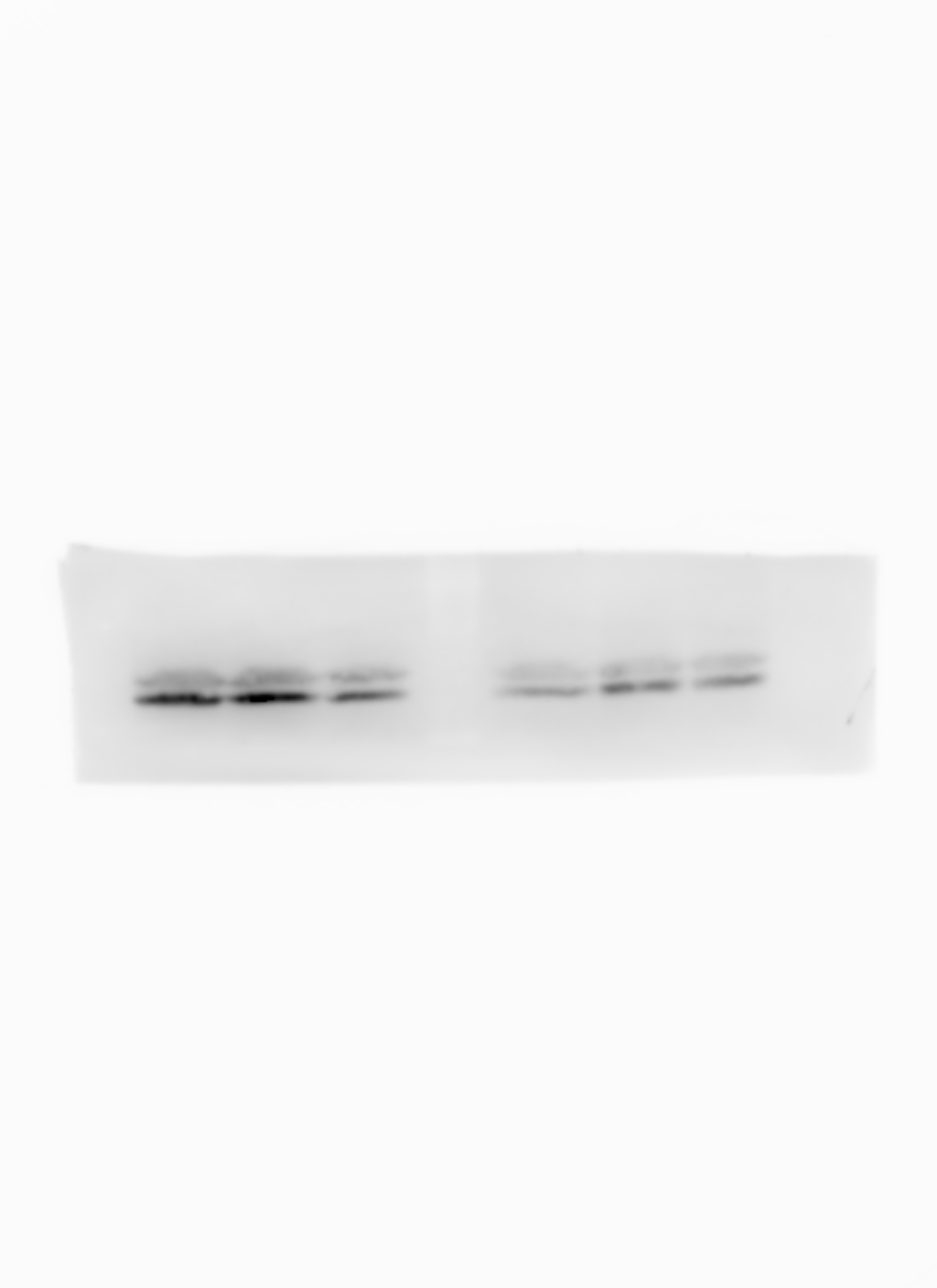

Supplement: Supplementary file 1 [file DataSheet1.zip › animal samples-WB supplementary materials/hippo-p-erk/hippo p-ERK 1/hippo p-ERK.tif]

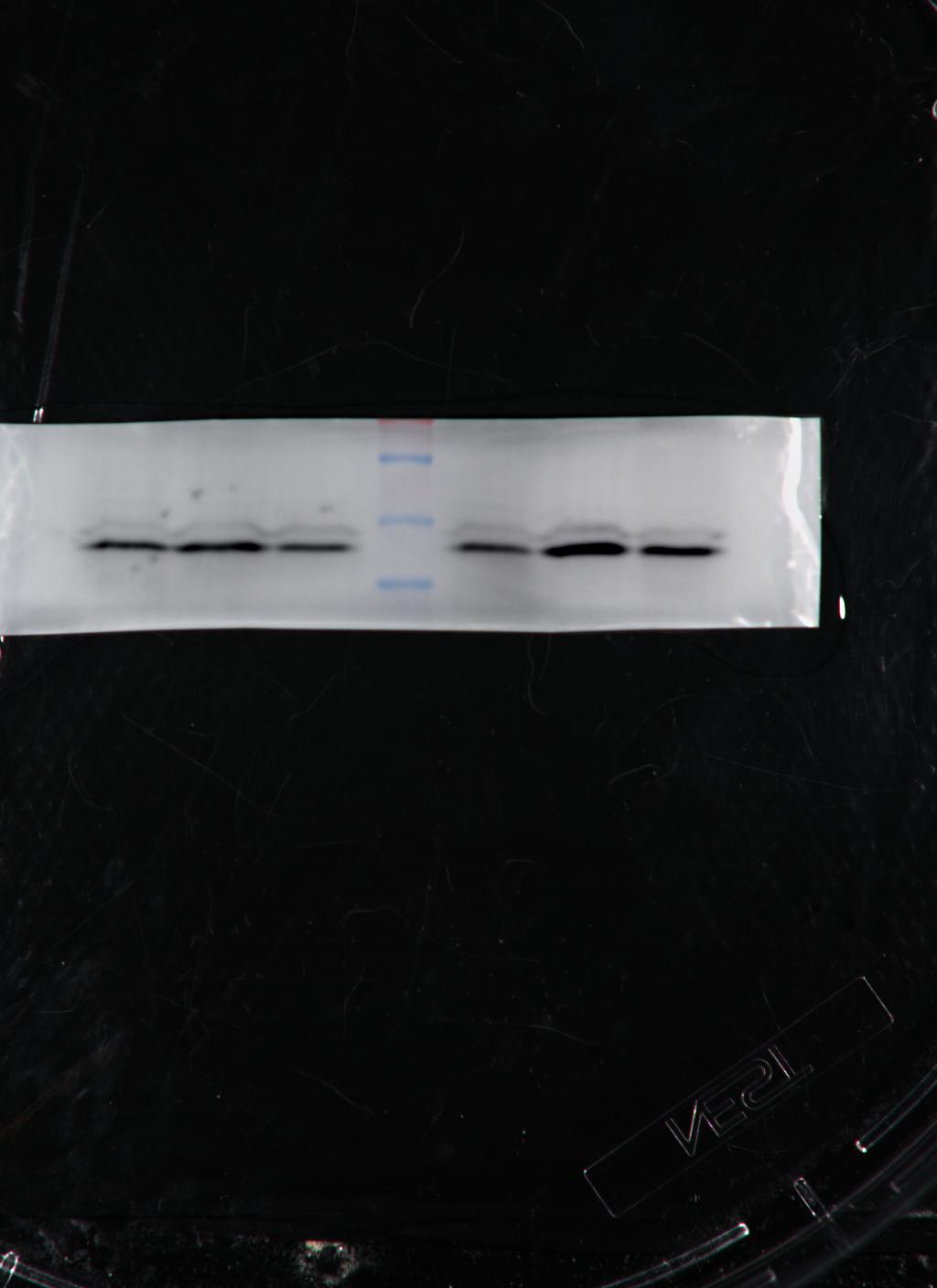

Supplement: Supplementary file 1 [file DataSheet1.zip › animal samples-WB supplementary materials/hippo-p-erk/hippo p-erk 2/hippo p-erk +Marker.jpg]

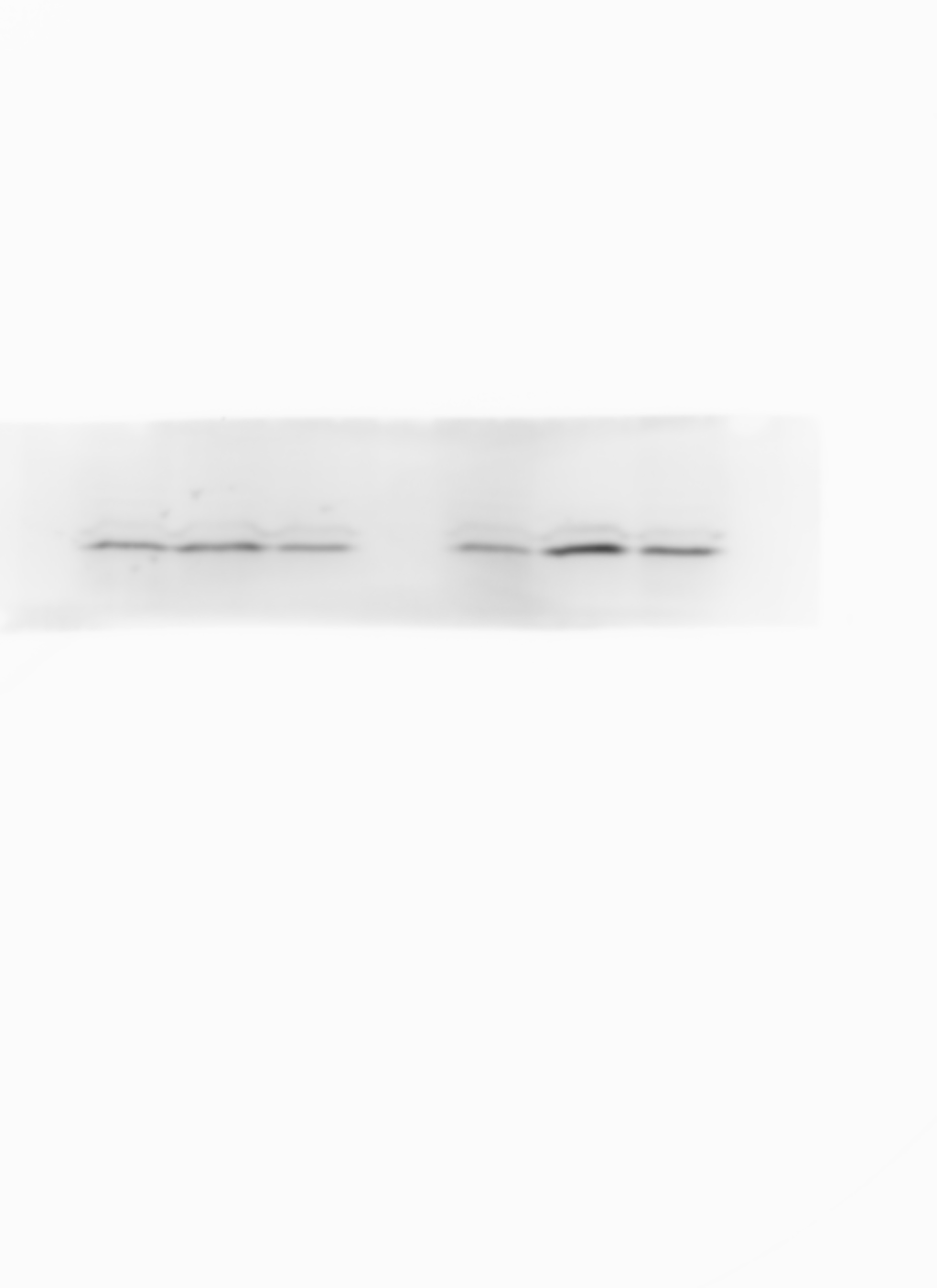

Supplement: Supplementary file 1 [file DataSheet1.zip › animal samples-WB supplementary materials/hippo-p-erk/hippo p-erk 2/hippo p-erk .tif]

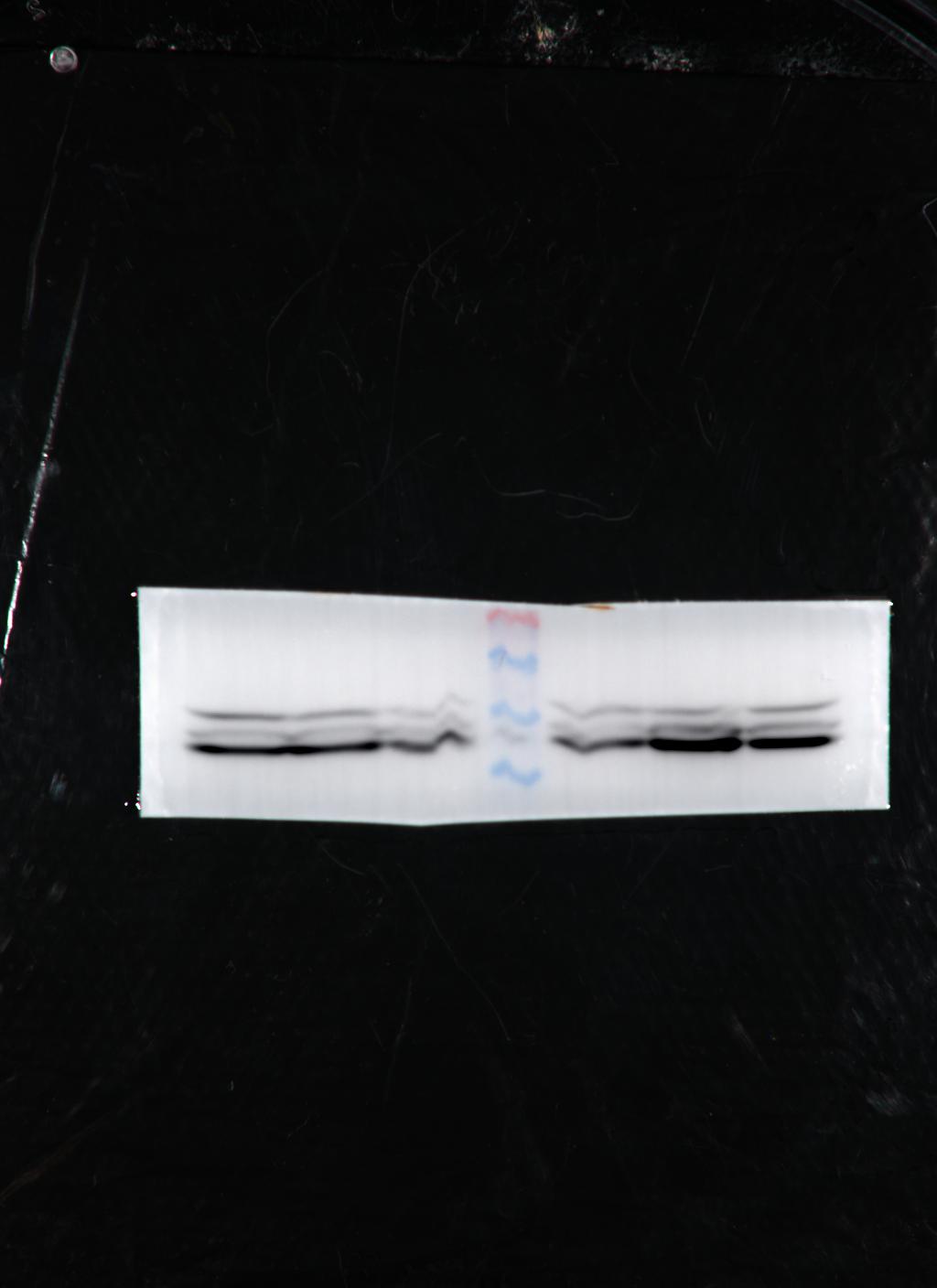

Supplement: Supplementary file 1 [file DataSheet1.zip › animal samples-WB supplementary materials/hippo-p-erk/hippo p-erk 3/hippo p-erk +Marker.jpg]

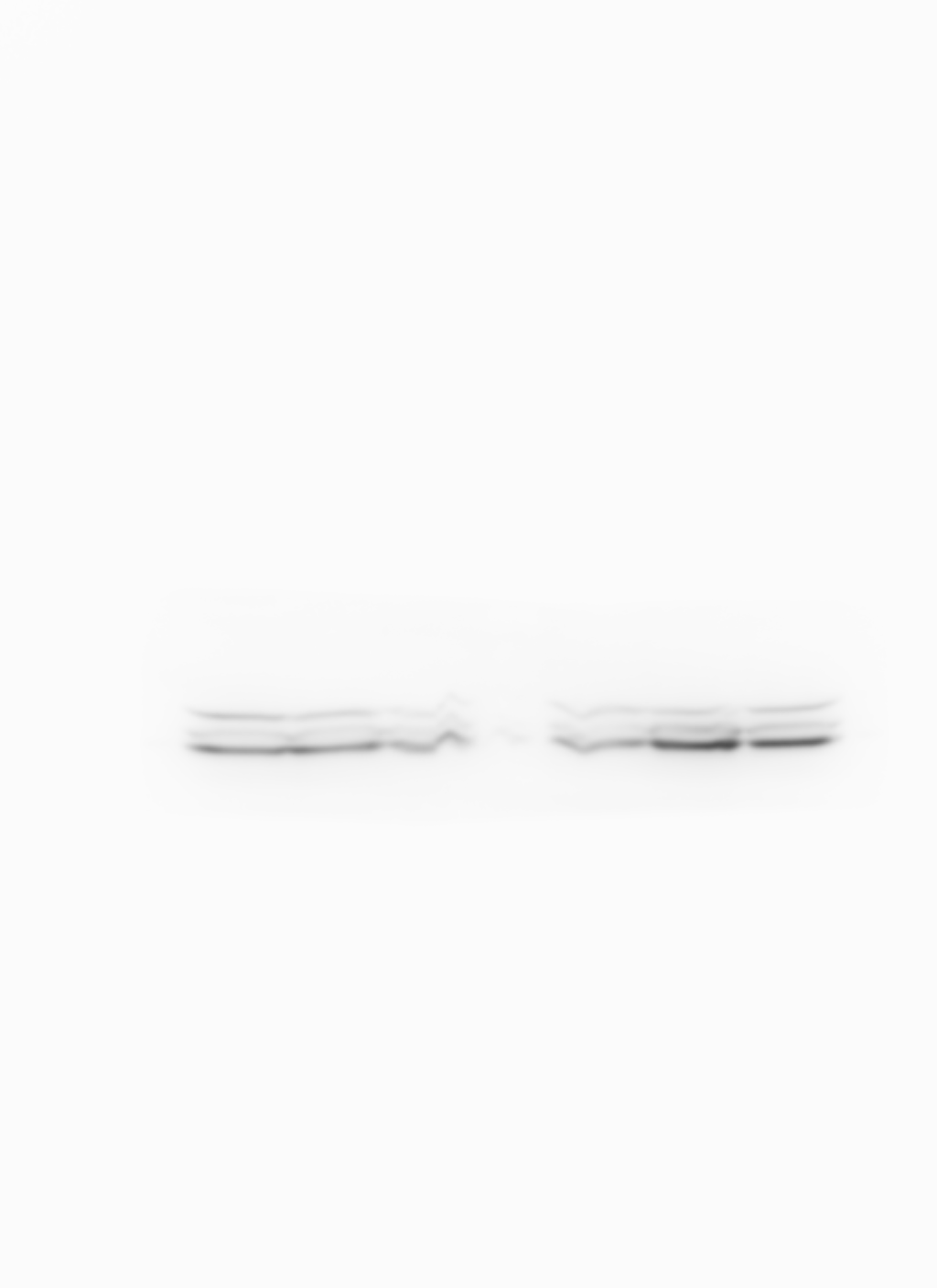

Supplement: Supplementary file 1 [file DataSheet1.zip › animal samples-WB supplementary materials/hippo-p-erk/hippo p-erk 3/hippo p-erk .tif]

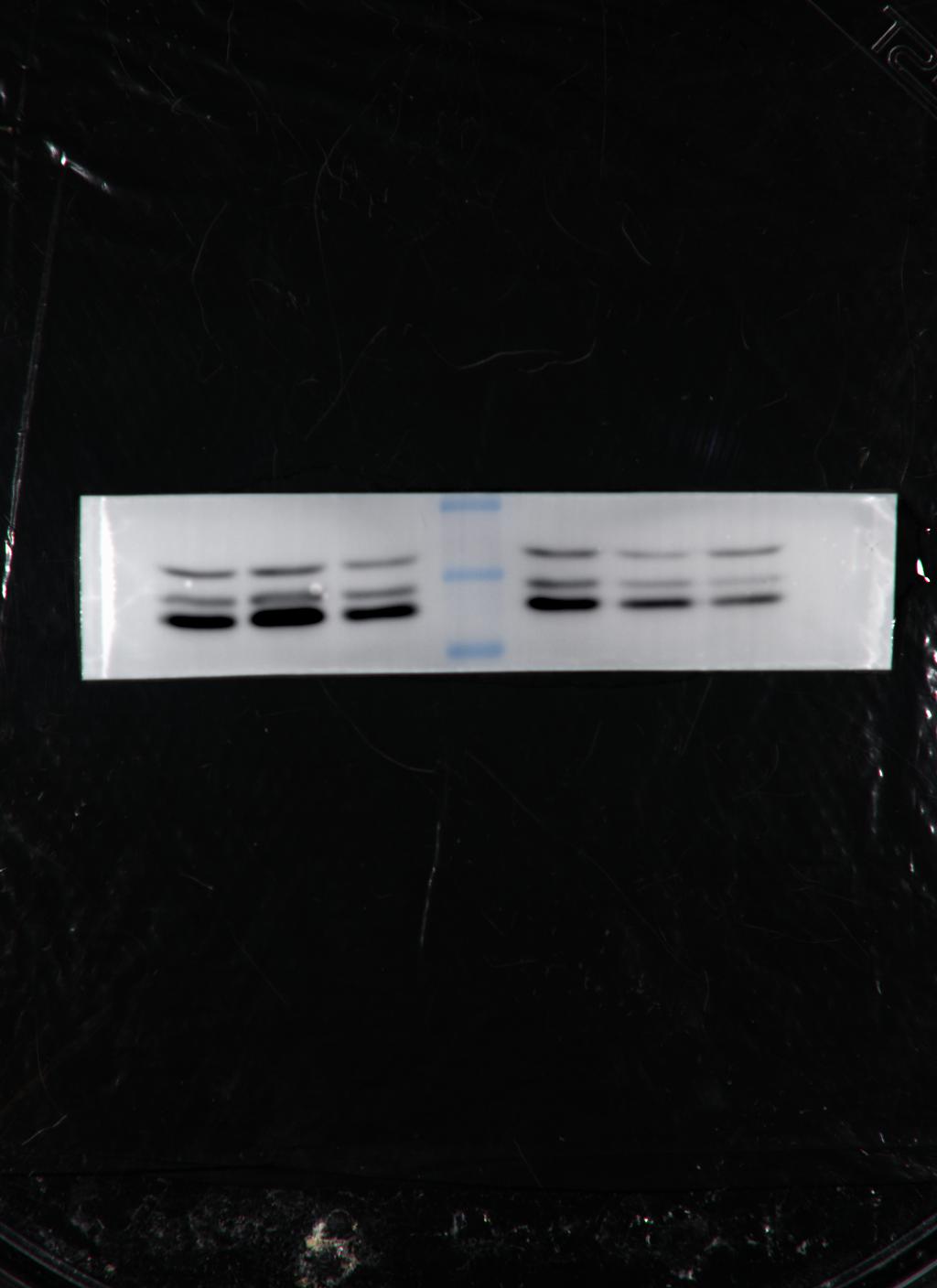

Supplement: Supplementary file 1 [file DataSheet1.zip › animal samples-WB supplementary materials/hippo-p-erk/hippo p-erk 4/hippo p-erk +Marker.jpg]

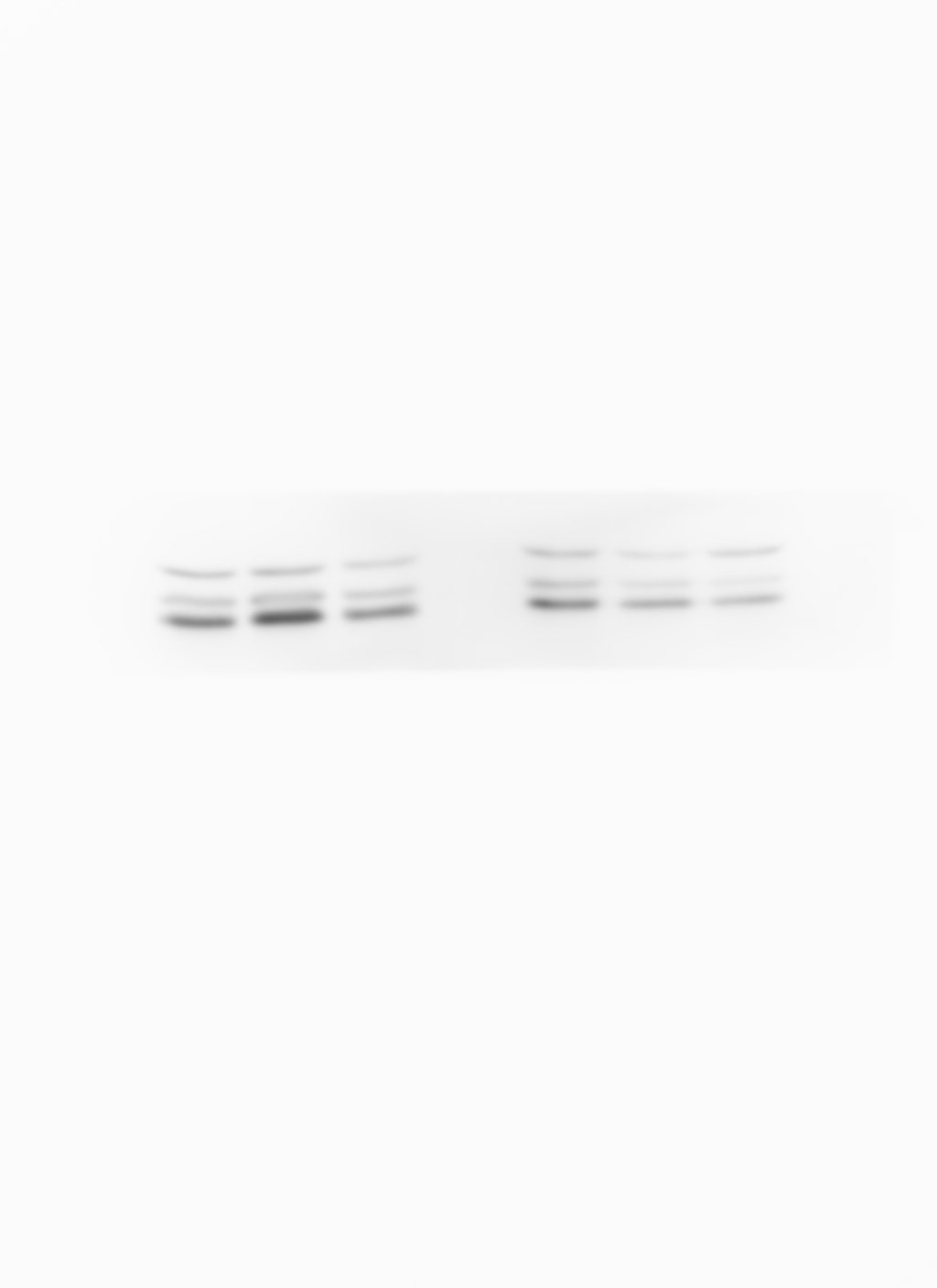

Supplement: Supplementary file 1 [file DataSheet1.zip › animal samples-WB supplementary materials/hippo-p-erk/hippo p-erk 4/hippo p-erk .tif]

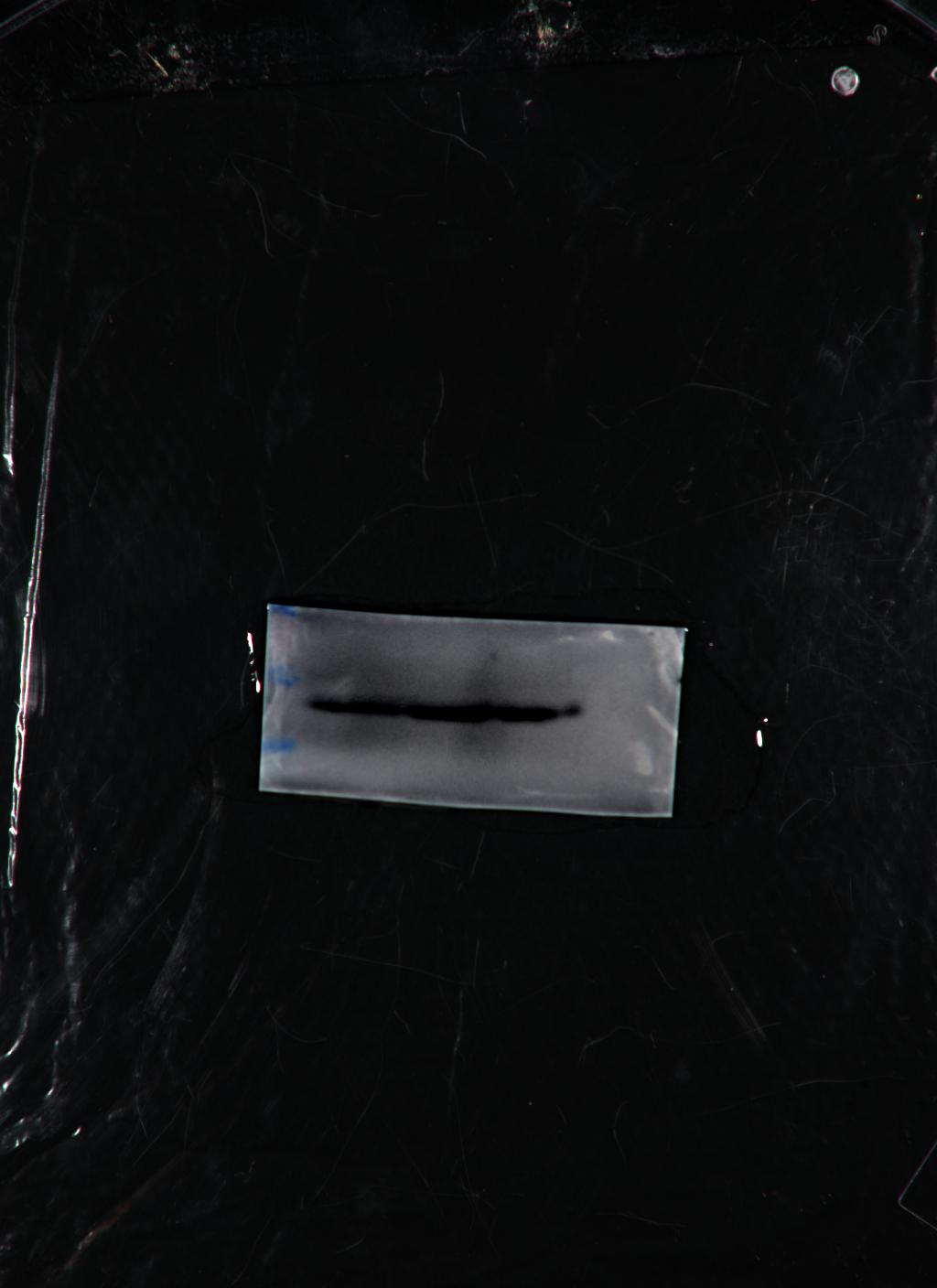

Supplement: Supplementary file 1 [file DataSheet1.zip › animal samples-WB supplementary materials/hippo-p-p38/hippo p38 1/hippo p38 +Marker.jpg]

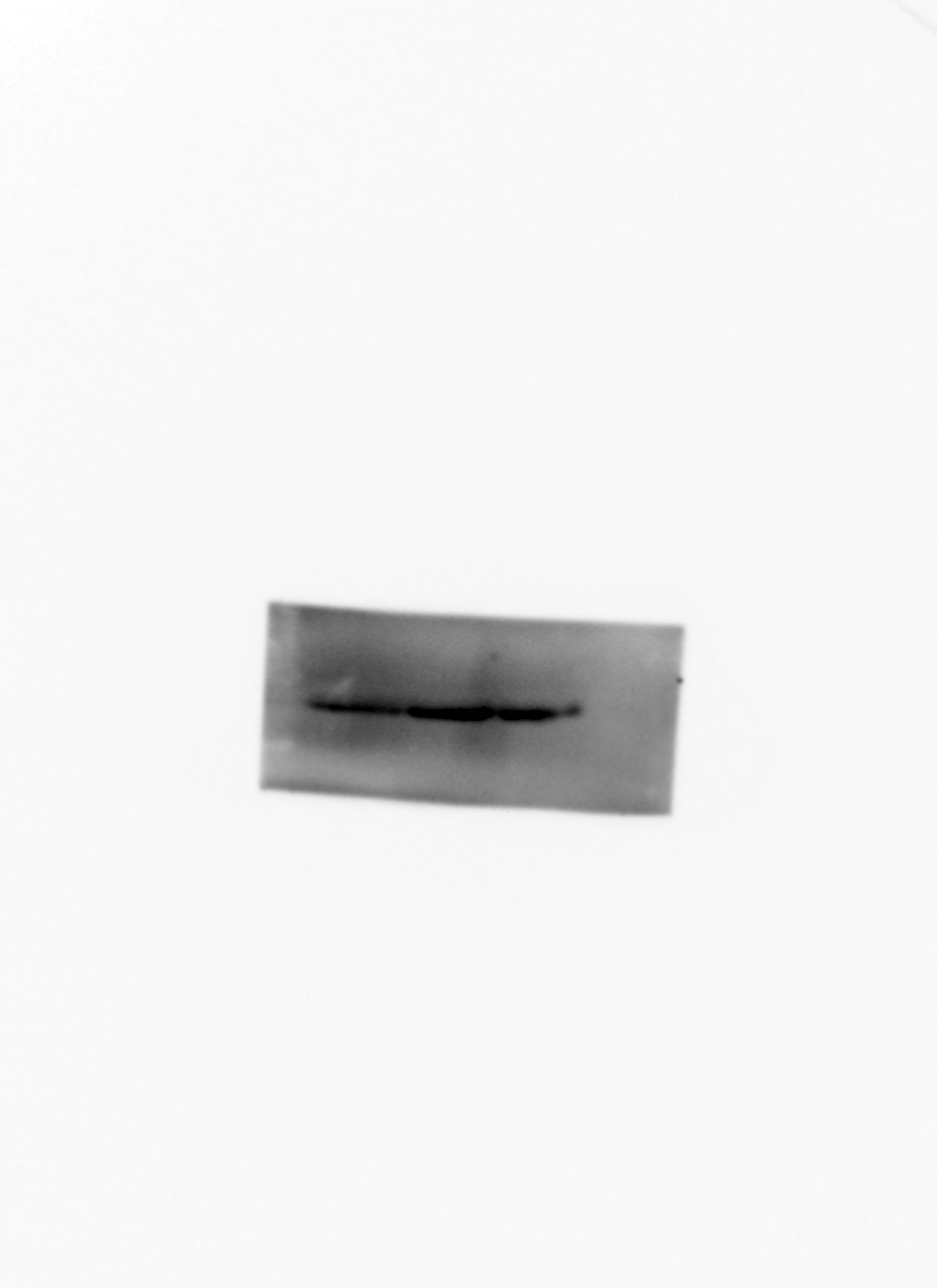

Supplement: Supplementary file 1 [file DataSheet1.zip › animal samples-WB supplementary materials/hippo-p-p38/hippo p38 1/hippo p38 .tif]

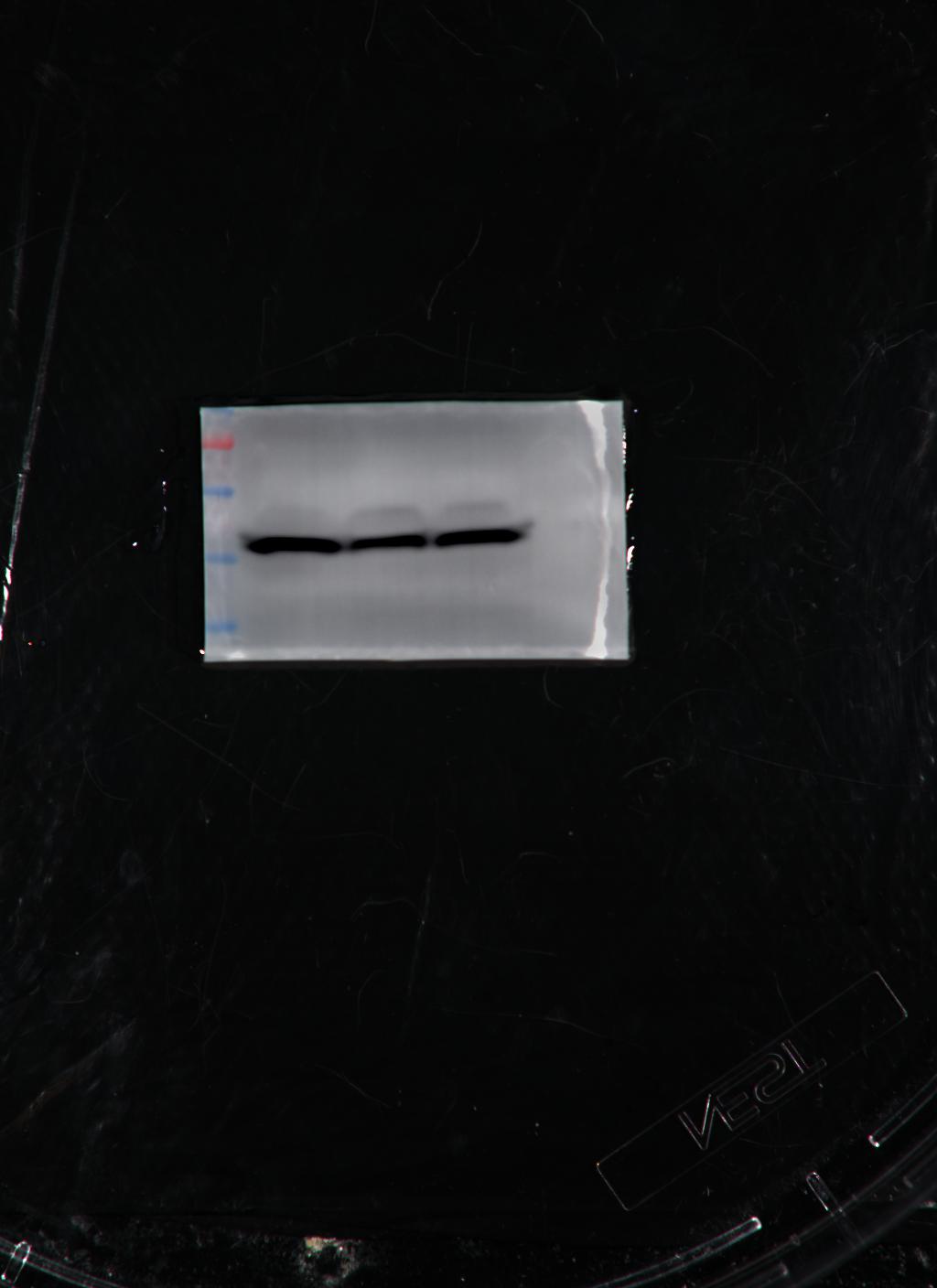

Supplement: Supplementary file 1 [file DataSheet1.zip › animal samples-WB supplementary materials/hippo-p-p38/hippo p38 2/hippo p38 +Marker.jpg]

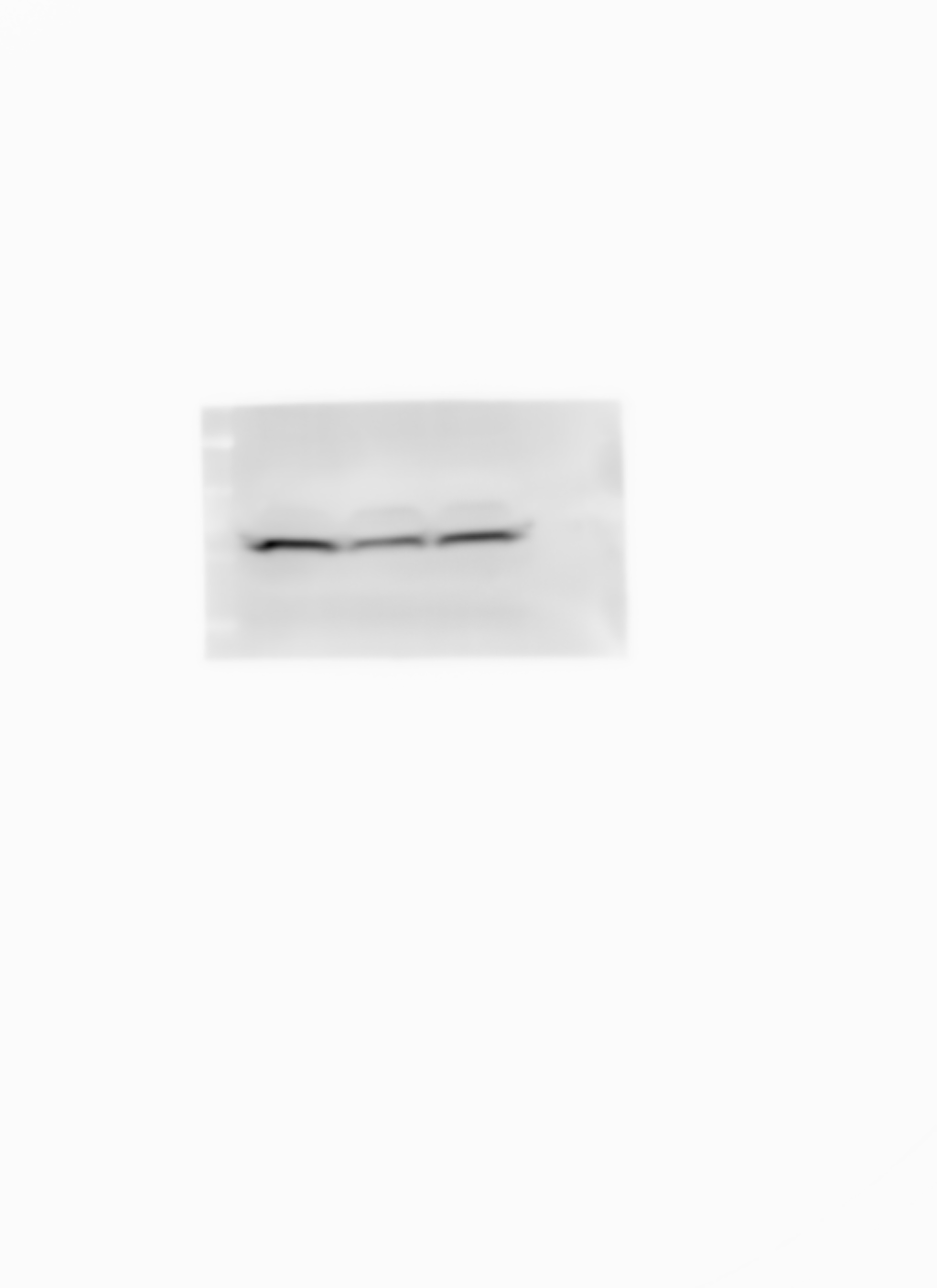

Supplement: Supplementary file 1 [file DataSheet1.zip › animal samples-WB supplementary materials/hippo-p-p38/hippo p38 2/hippo p38.tif]

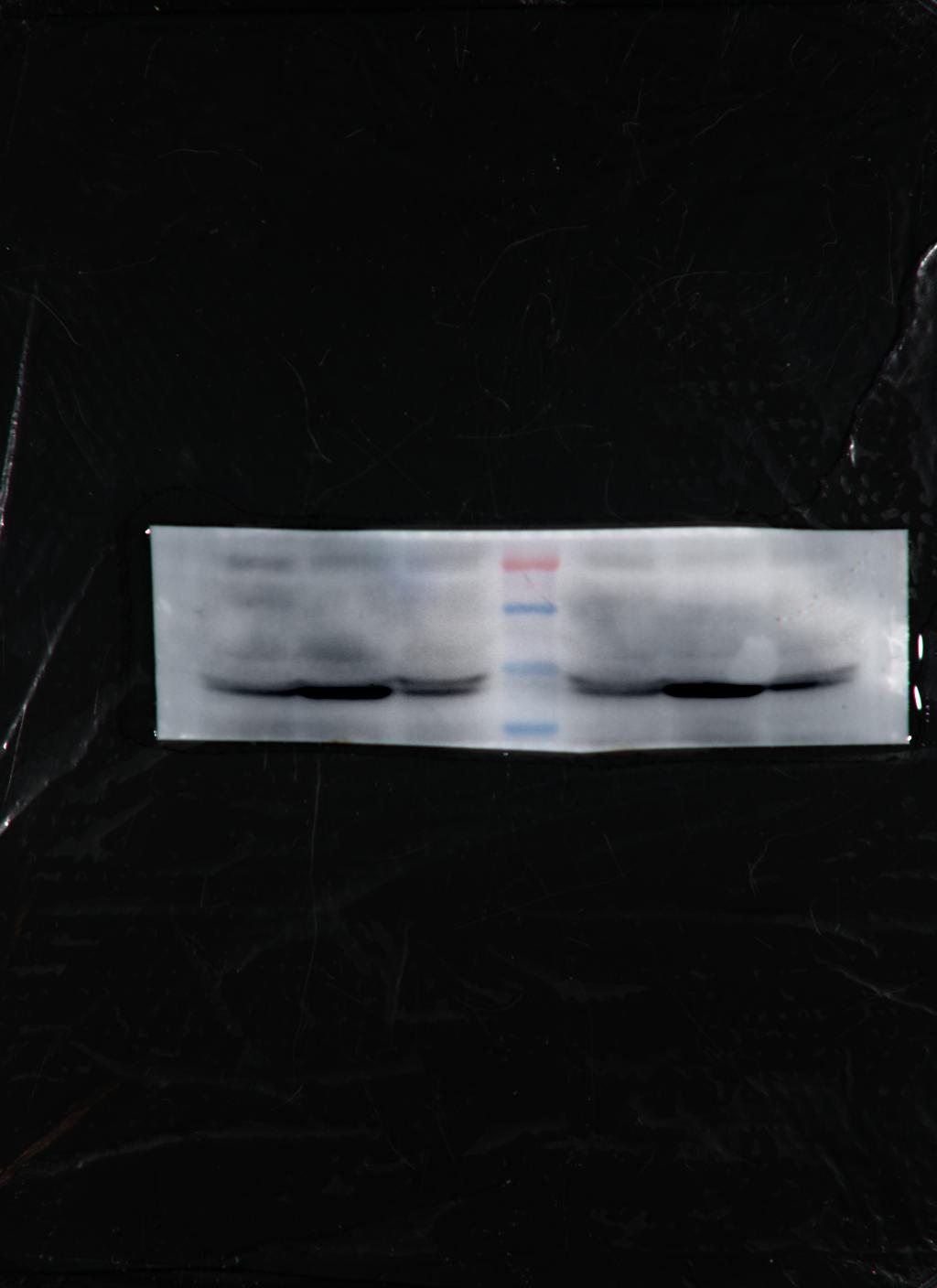

Supplement: Supplementary file 1 [file DataSheet1.zip › animal samples-WB supplementary materials/hippo-p-p38/hippo pp38 1/hippo pp38 +Marker.jpg]

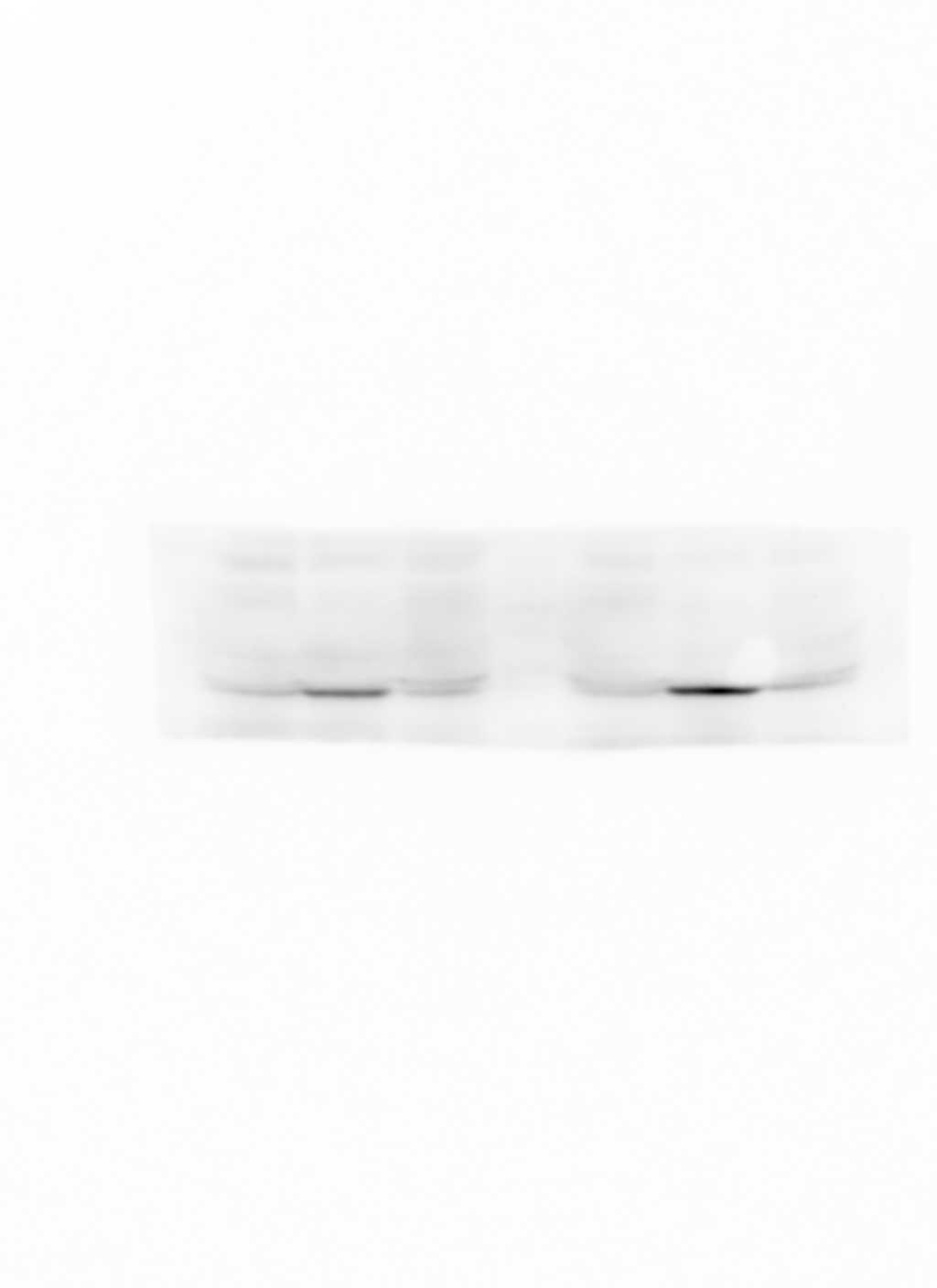

Supplement: Supplementary file 1 [file DataSheet1.zip › animal samples-WB supplementary materials/hippo-p-p38/hippo pp38 1/hippo pp38.tif]

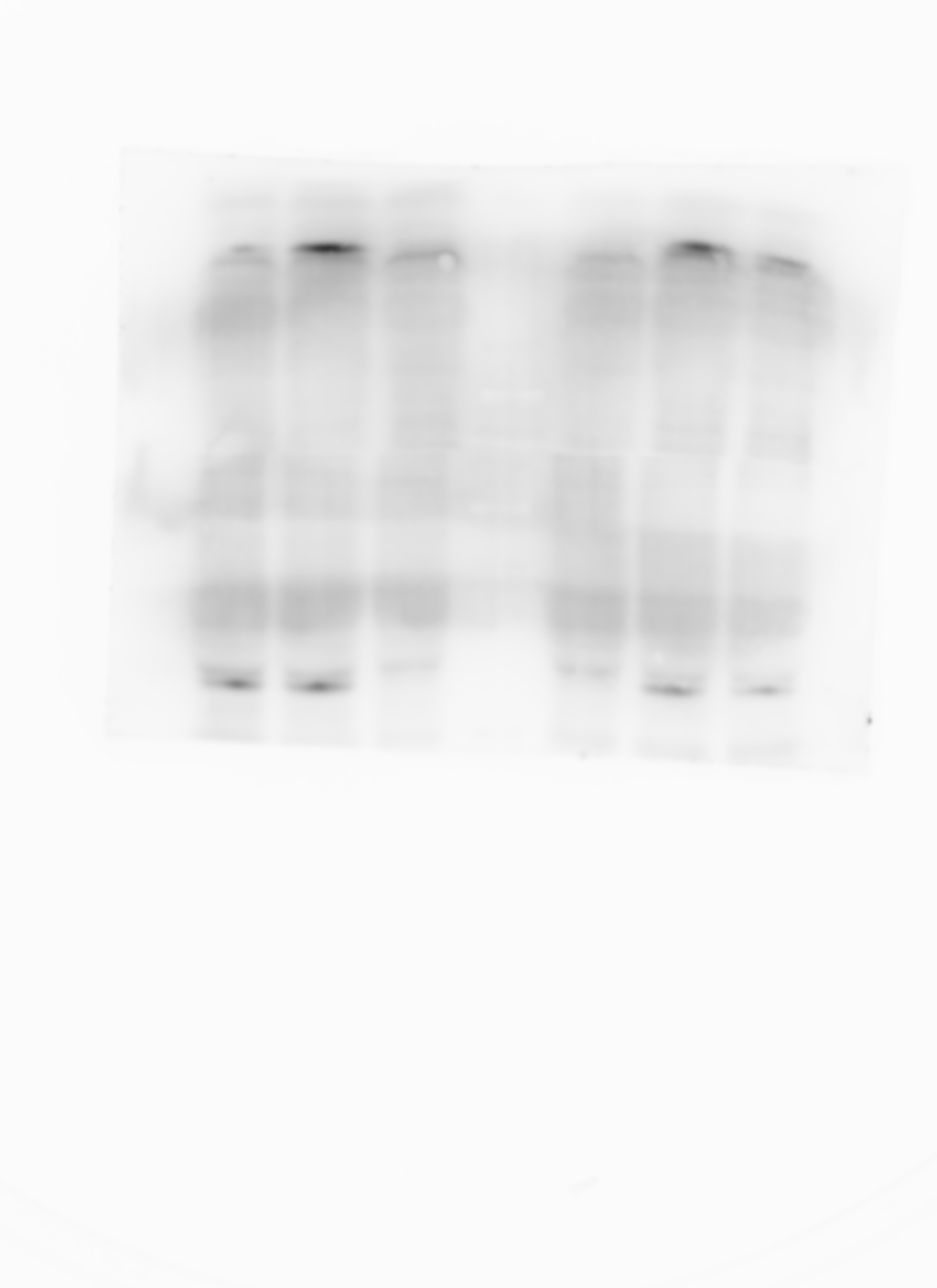

Supplement: Supplementary file 1 [file DataSheet1.zip › animal samples-WB supplementary materials/hippo-p-p38/hippo pp38 2/hippo pp38 .tif]

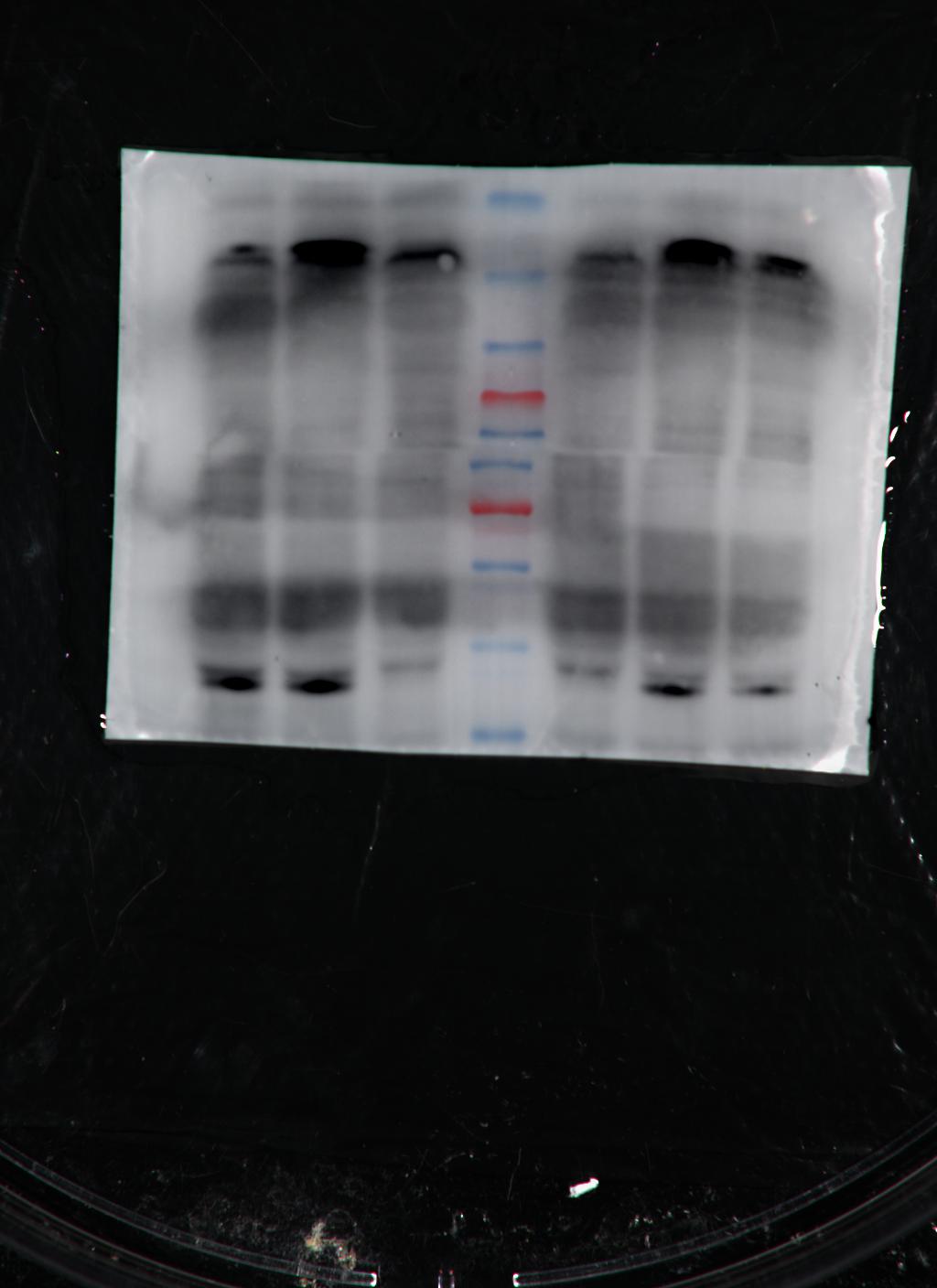

Supplement: Supplementary file 1 [file DataSheet1.zip › animal samples-WB supplementary materials/hippo-p-p38/hippo pp38 2/hippo pp38+Marker.jpg]

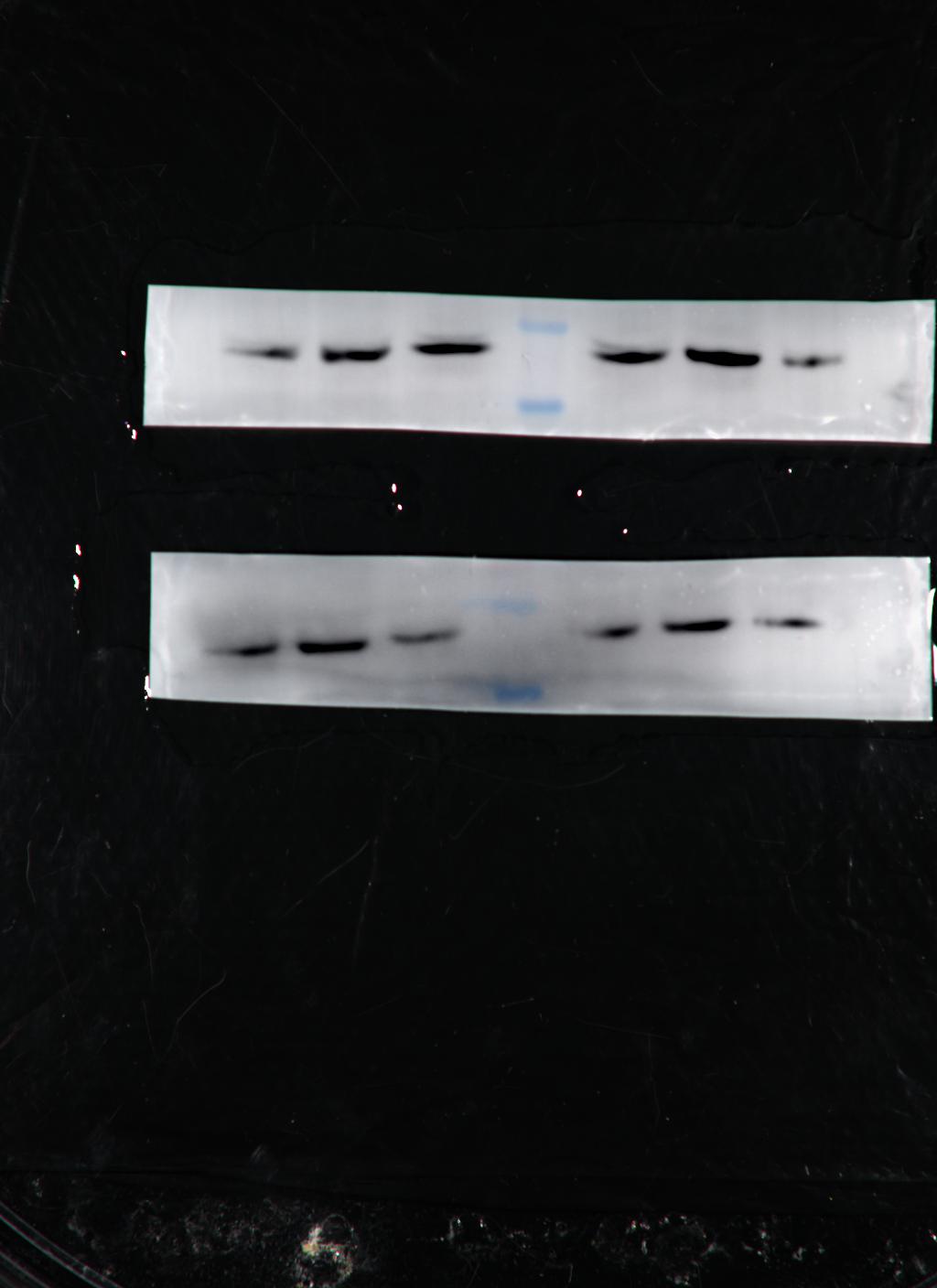

Supplement: Supplementary file 1 [file DataSheet1.zip › animal samples-WB supplementary materials/hippo-p-p38/hippo pp38 3/hippo pp38 +Marker.jpg]

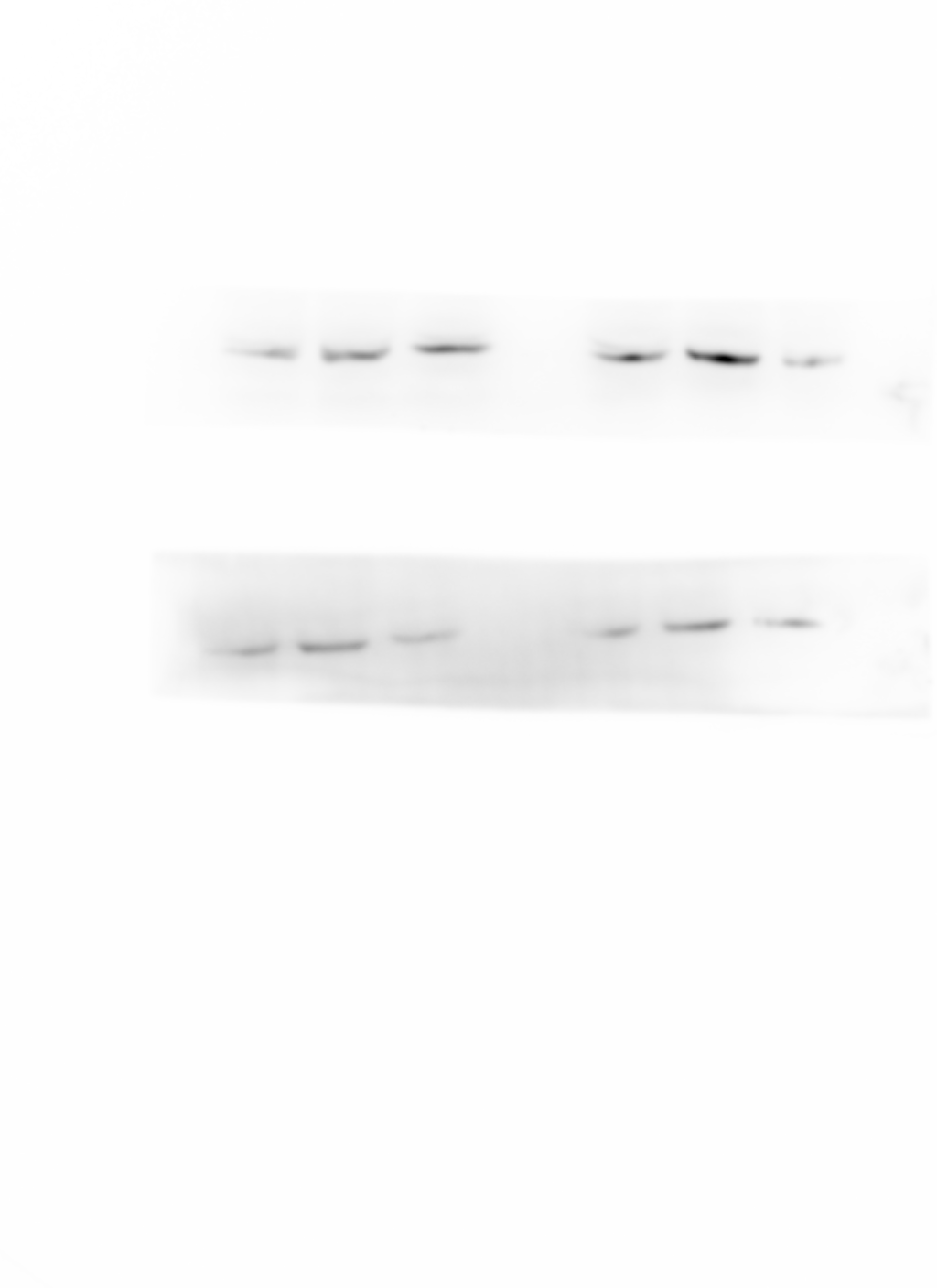

Supplement: Supplementary file 1 [file DataSheet1.zip › animal samples-WB supplementary materials/hippo-p-p38/hippo pp38 3/hippo pp38.tif]

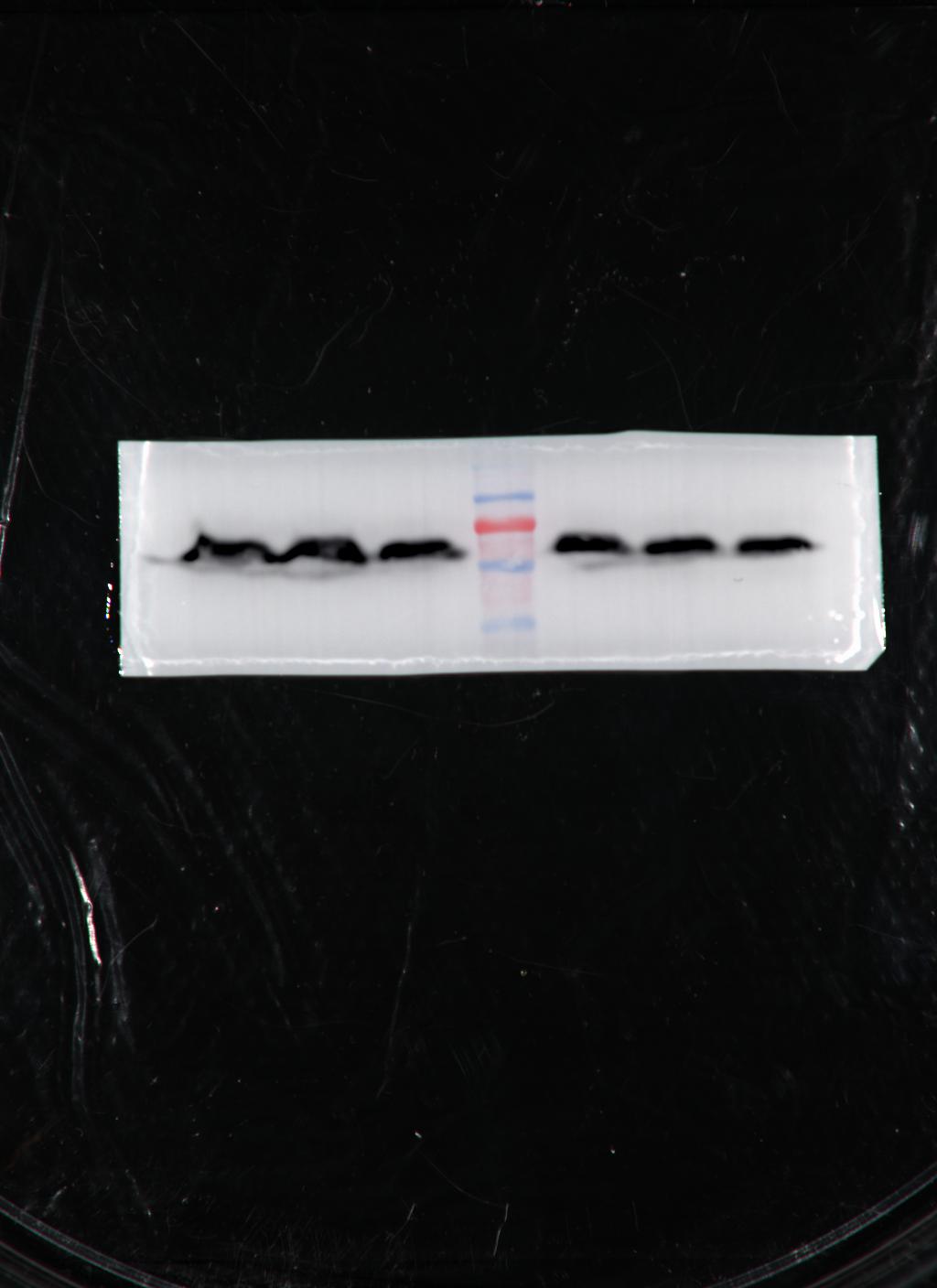

Supplement: Supplementary file 1 [file DataSheet1.zip › animal samples-WB supplementary materials/hippo-p-p65/hippo p65 1/hippo p65 +Marker.jpg]

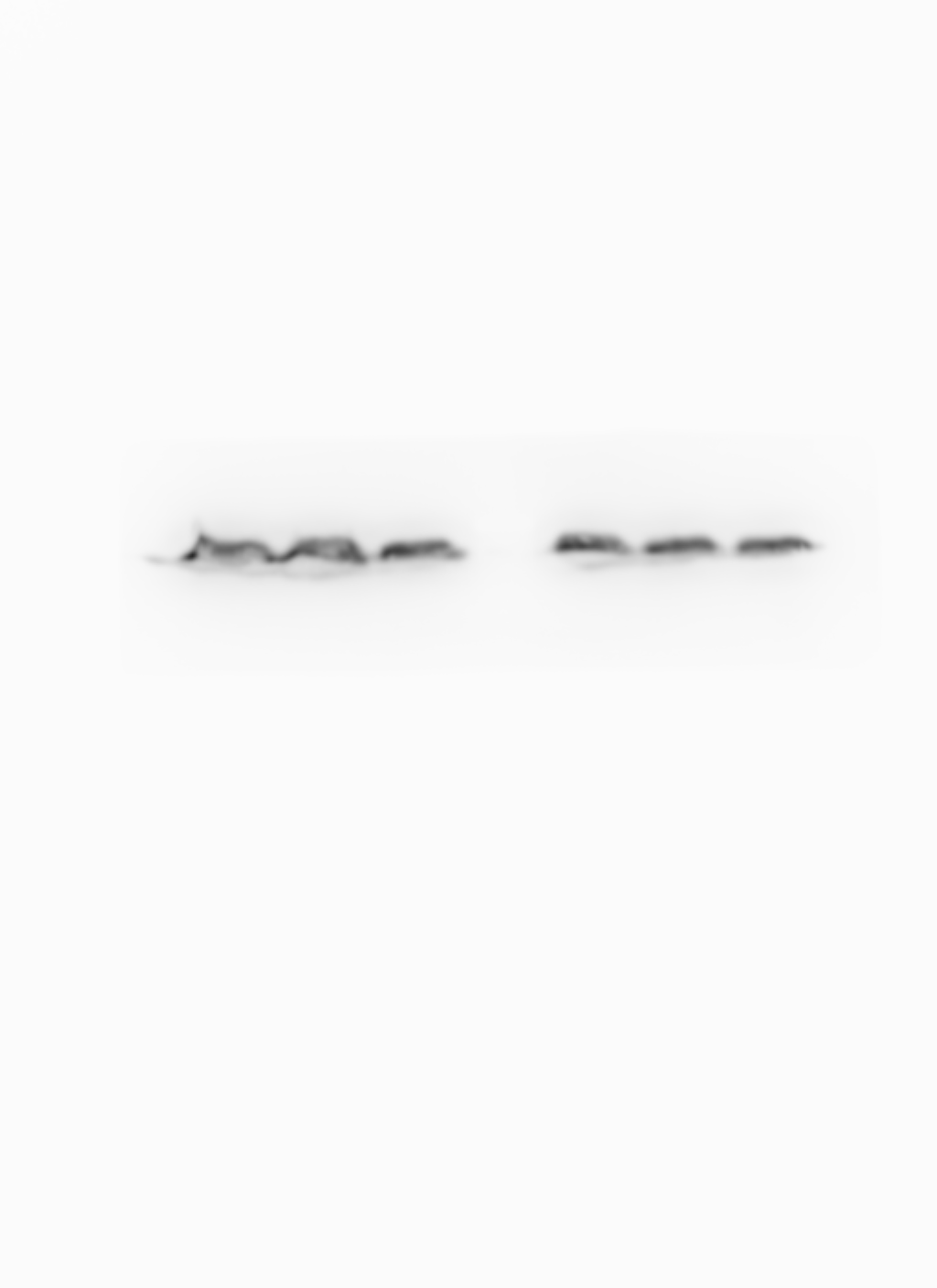

Supplement: Supplementary file 1 [file DataSheet1.zip › animal samples-WB supplementary materials/hippo-p-p65/hippo p65 1/hippo p65 .tif]

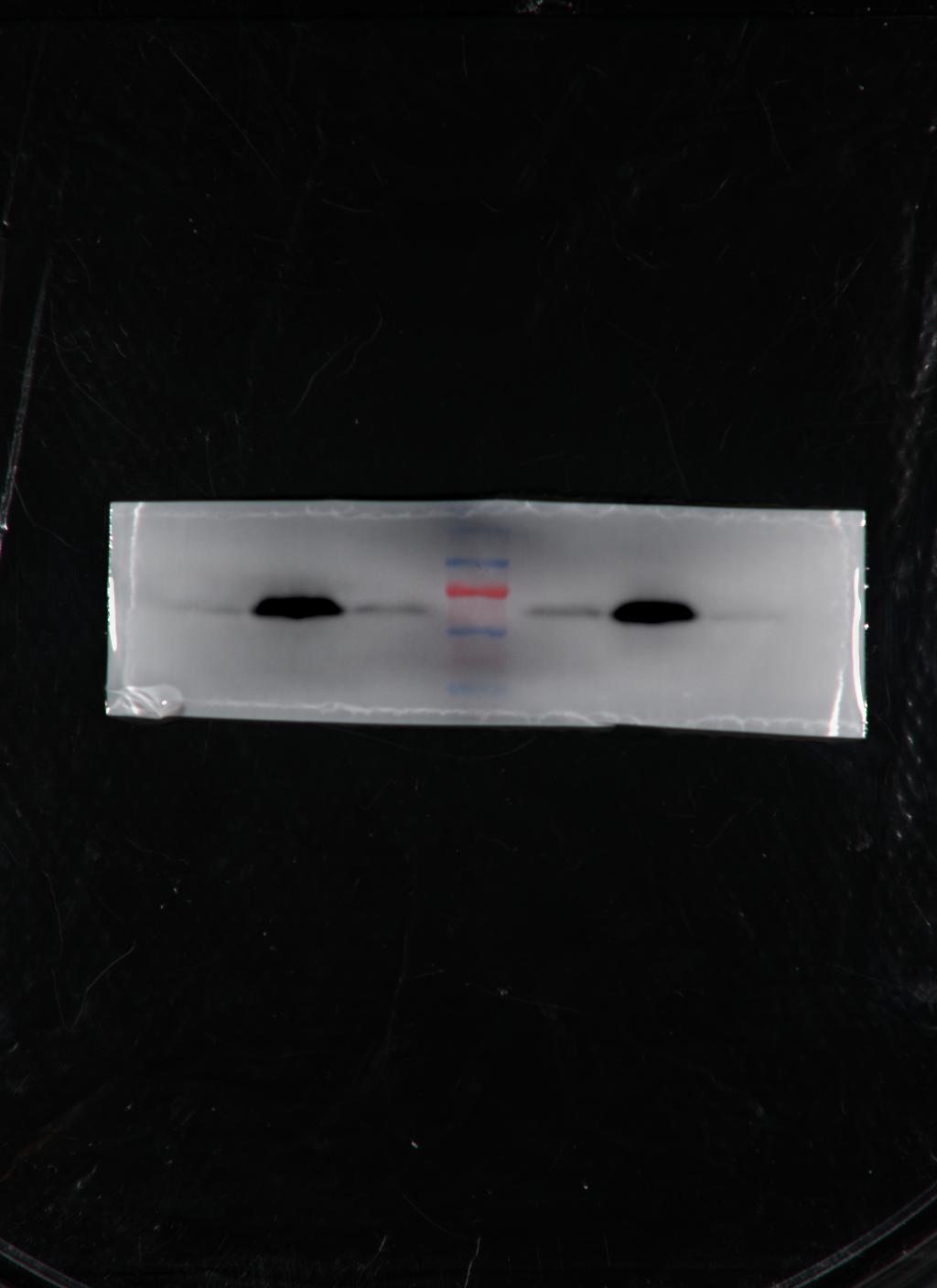

Supplement: Supplementary file 1 [file DataSheet1.zip › animal samples-WB supplementary materials/hippo-p-p65/hippo pp65 1/hippo pp65 +Marker.jpg]

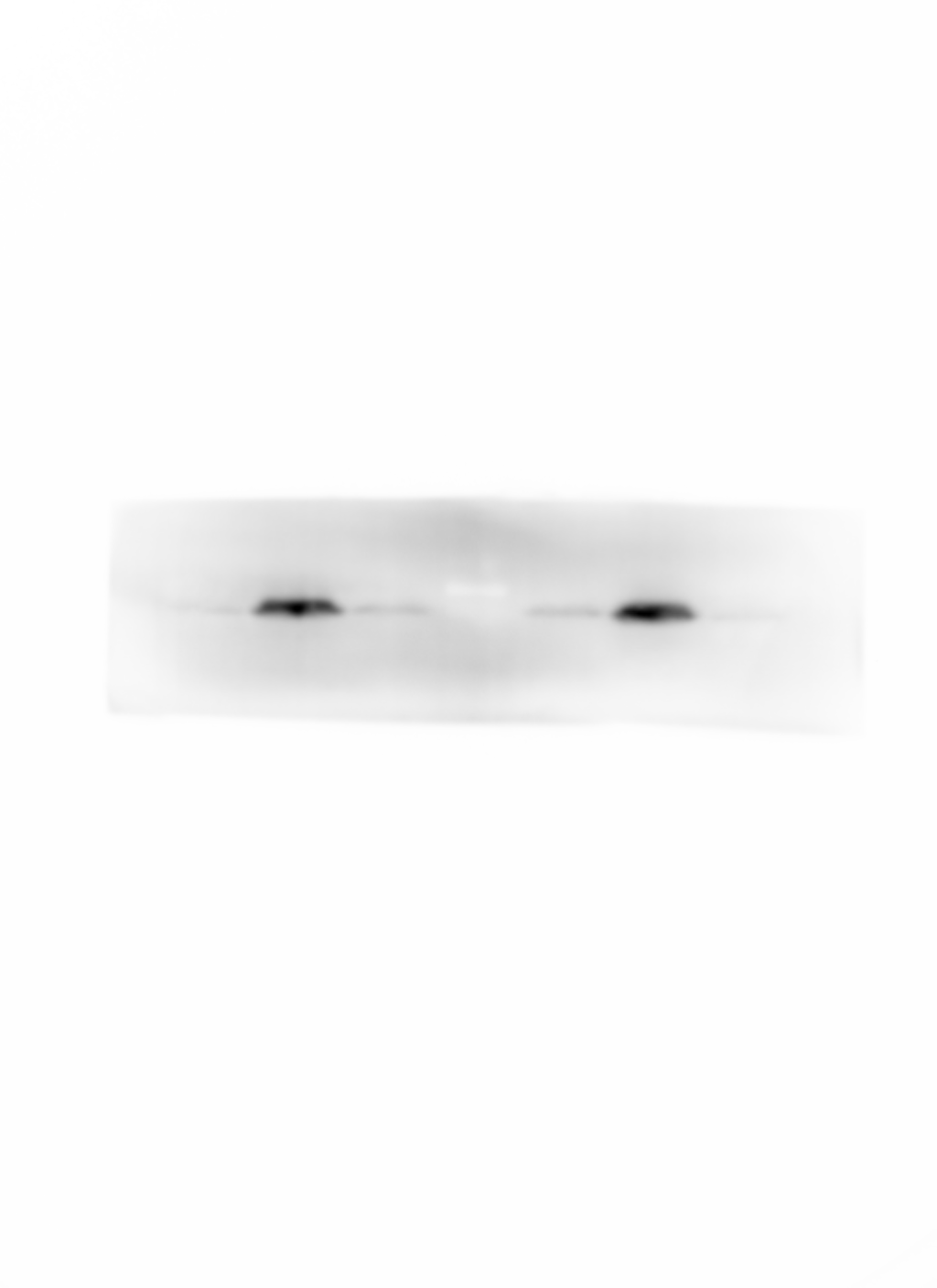

Supplement: Supplementary file 1 [file DataSheet1.zip › animal samples-WB supplementary materials/hippo-p-p65/hippo pp65 1/hippo pp65 .tif]

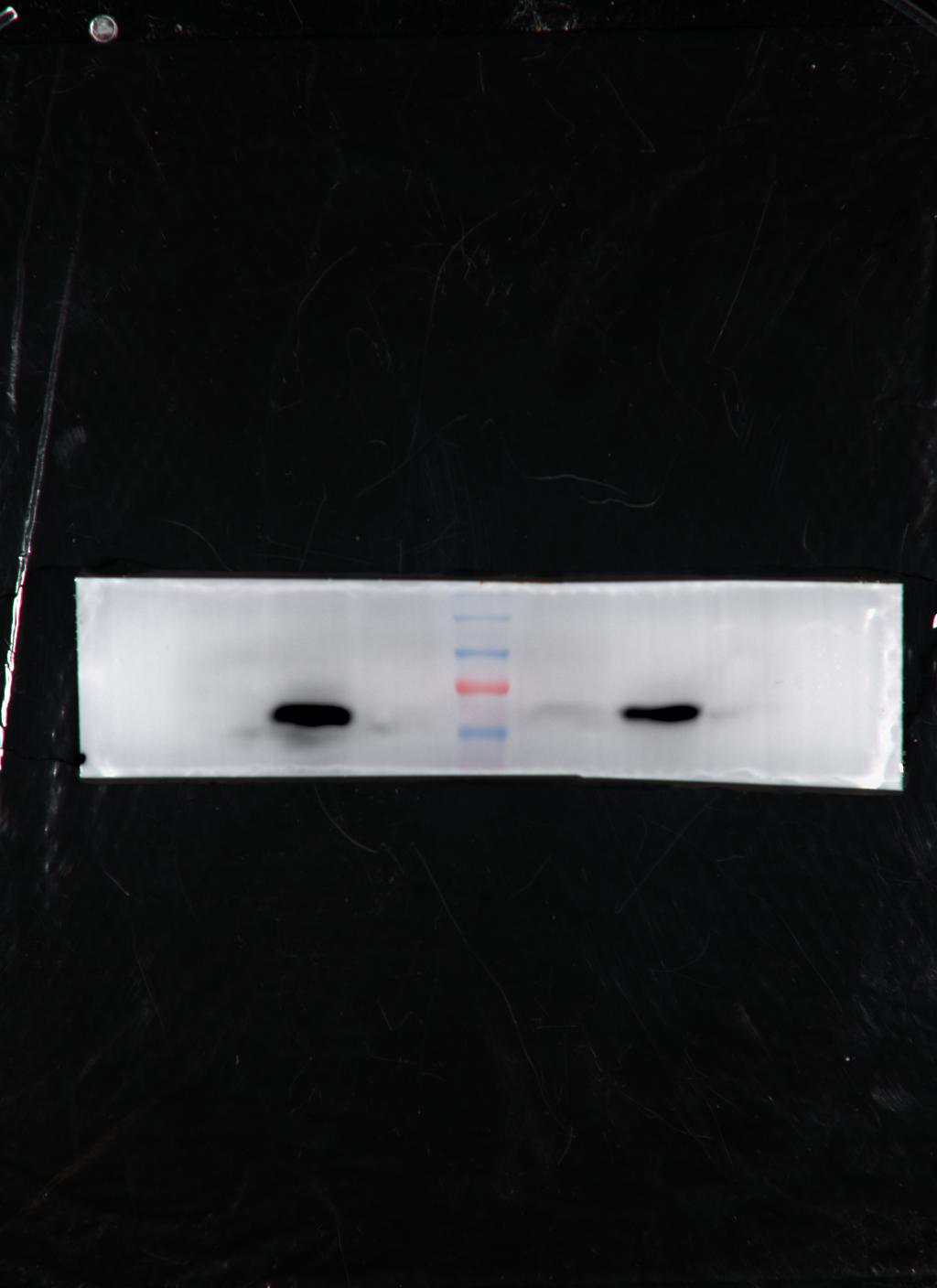

Supplement: Supplementary file 1 [file DataSheet1.zip › animal samples-WB supplementary materials/hippo-p-p65/hippo pp65 2/hippo pp65 +Marker.jpg]

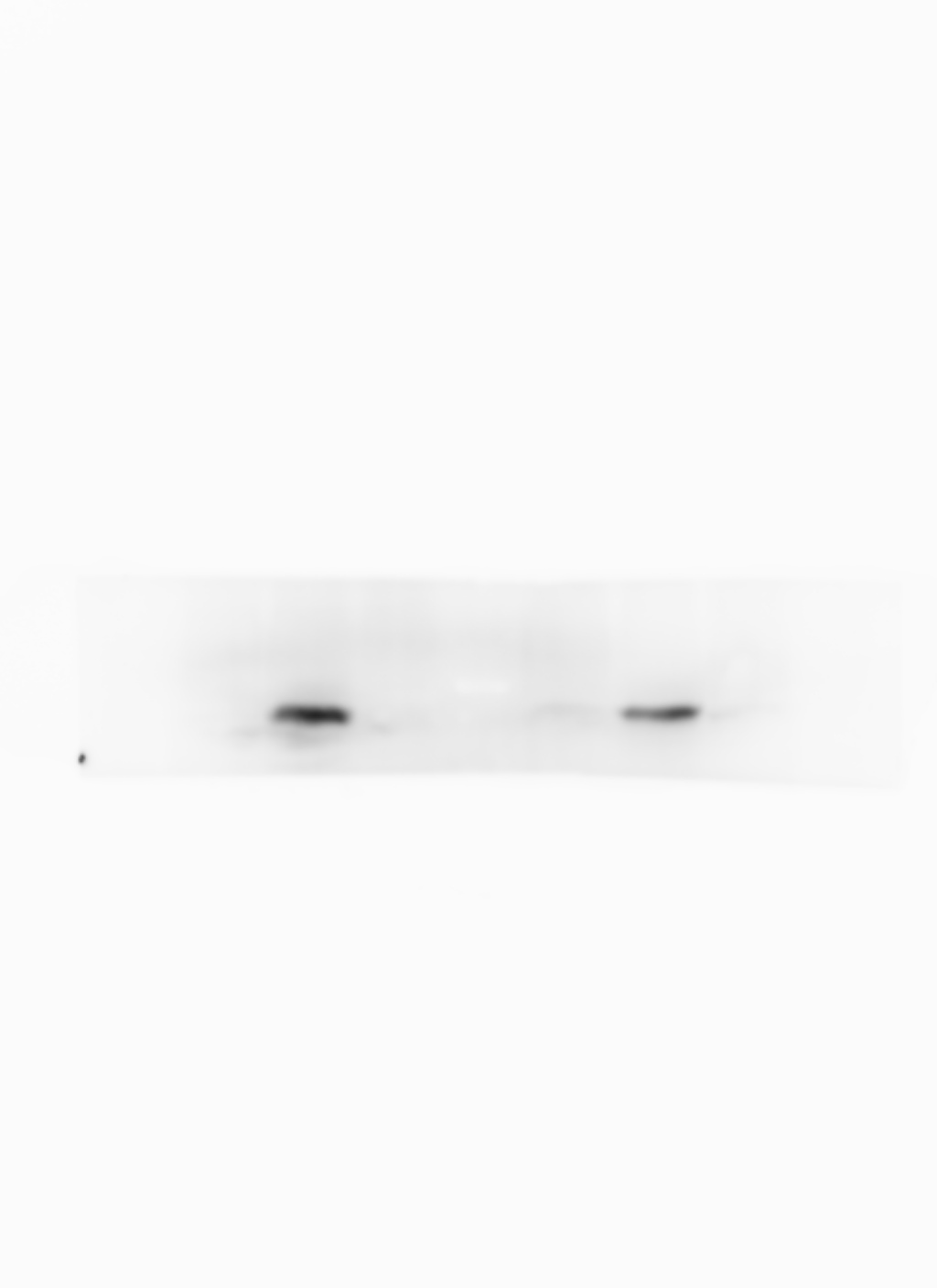

Supplement: Supplementary file 1 [file DataSheet1.zip › animal samples-WB supplementary materials/hippo-p-p65/hippo pp65 2/hippo pp65 .tif]

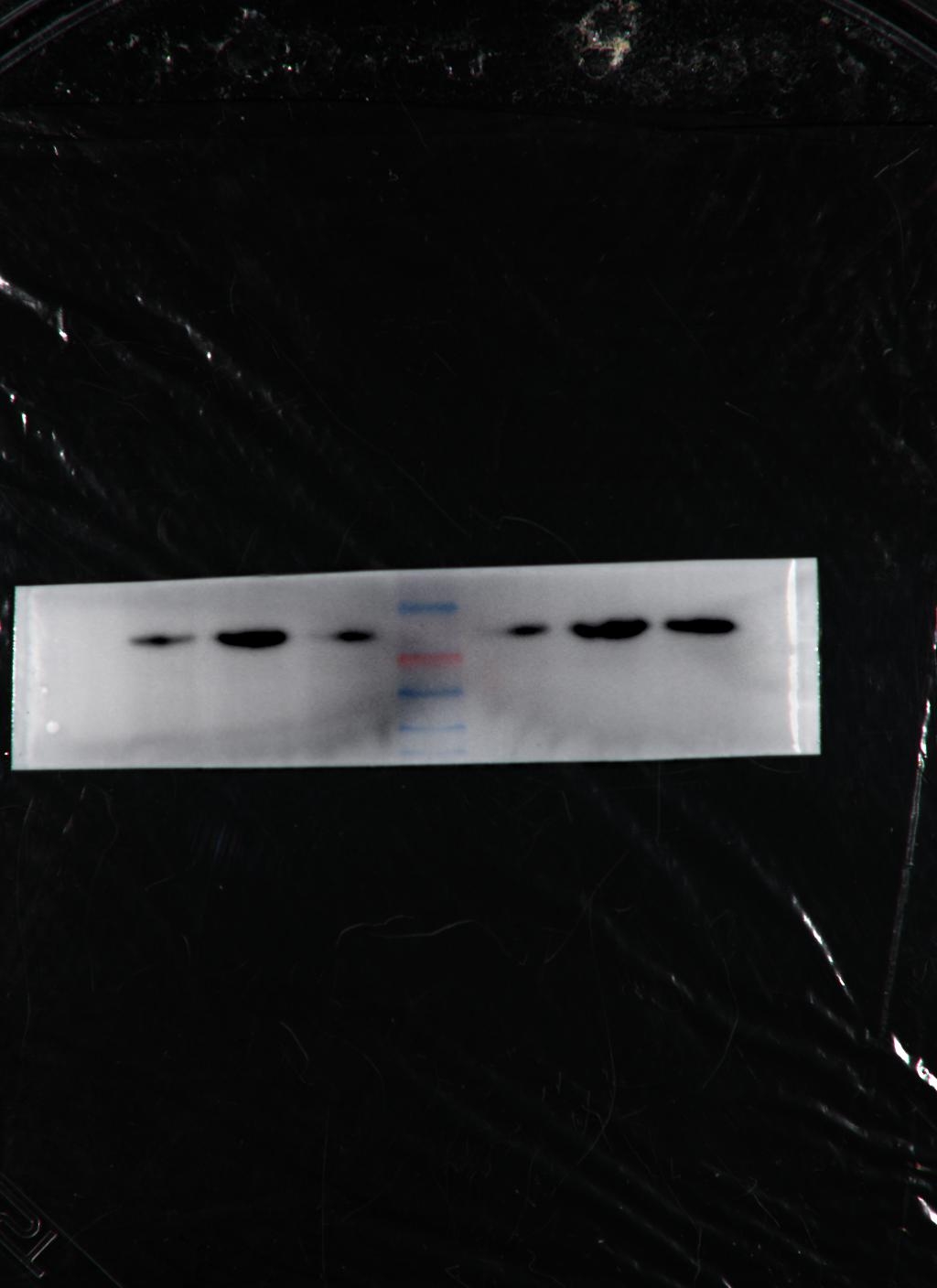

Supplement: Supplementary file 1 [file DataSheet1.zip › animal samples-WB supplementary materials/hippo-p-p65/hippo pp65 3/hippo pp65 +Marker.jpg]

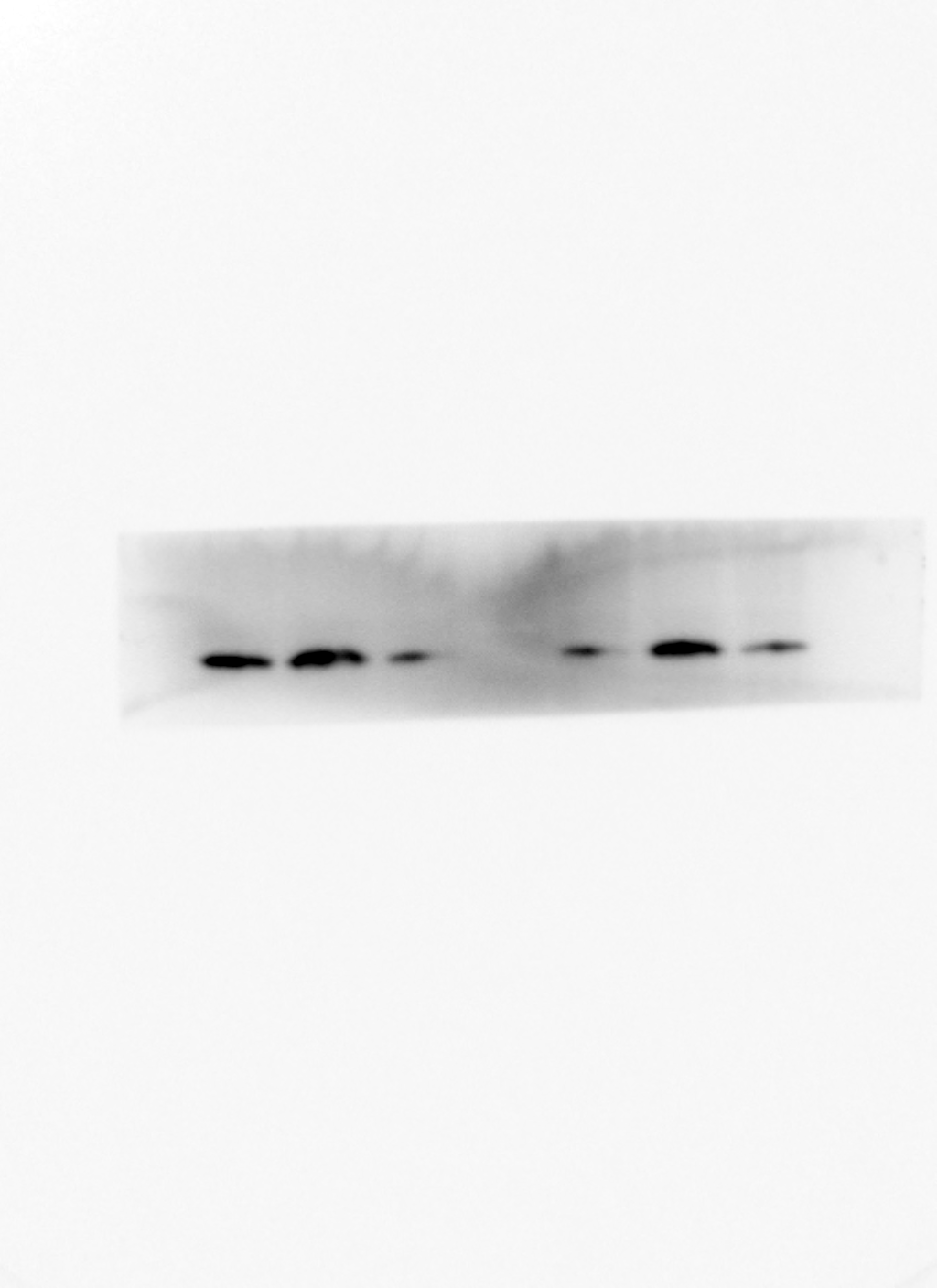

Supplement: Supplementary file 1 [file DataSheet1.zip › animal samples-WB supplementary materials/hippo-p-p65/hippo pp65 3/hippo pp65 .tif]

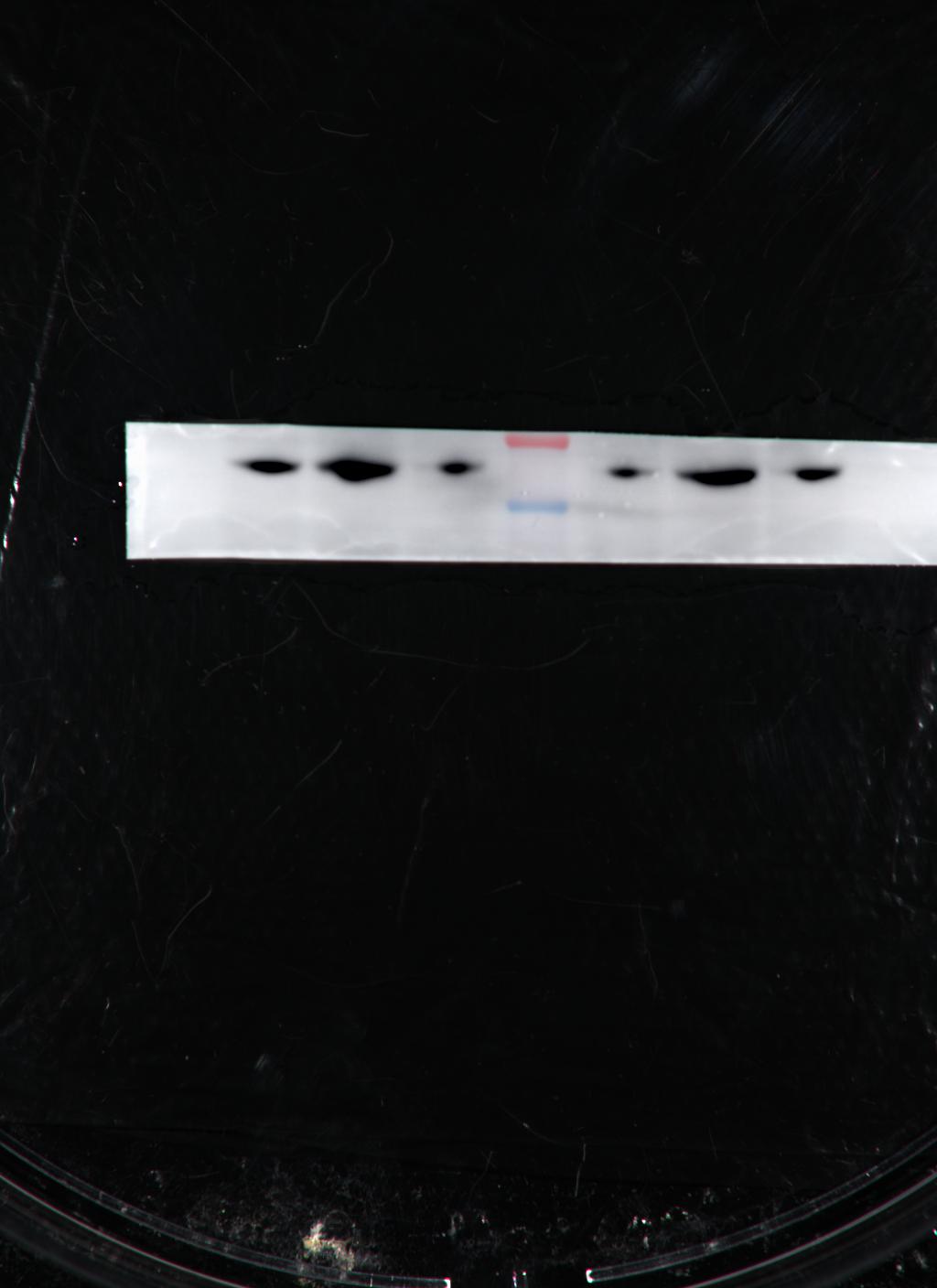

Supplement: Supplementary file 1 [file DataSheet1.zip › animal samples-WB supplementary materials/hippo-p-p65/hippo pp65 4/hippo pp65 +Marker.jpg]

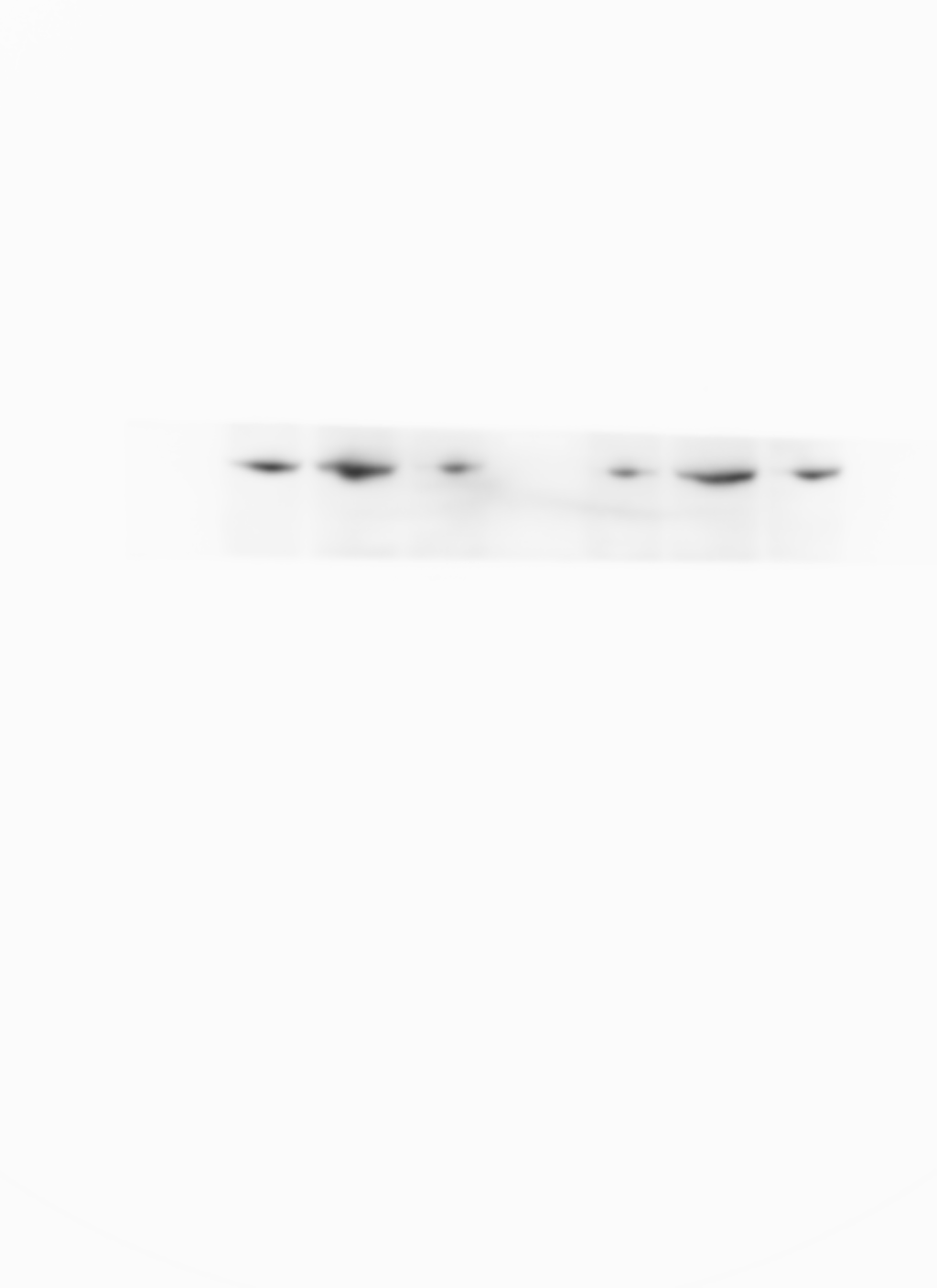

Supplement: Supplementary file 1 [file DataSheet1.zip › animal samples-WB supplementary materials/hippo-p-p65/hippo pp65 4/hippo pp65 .tif]

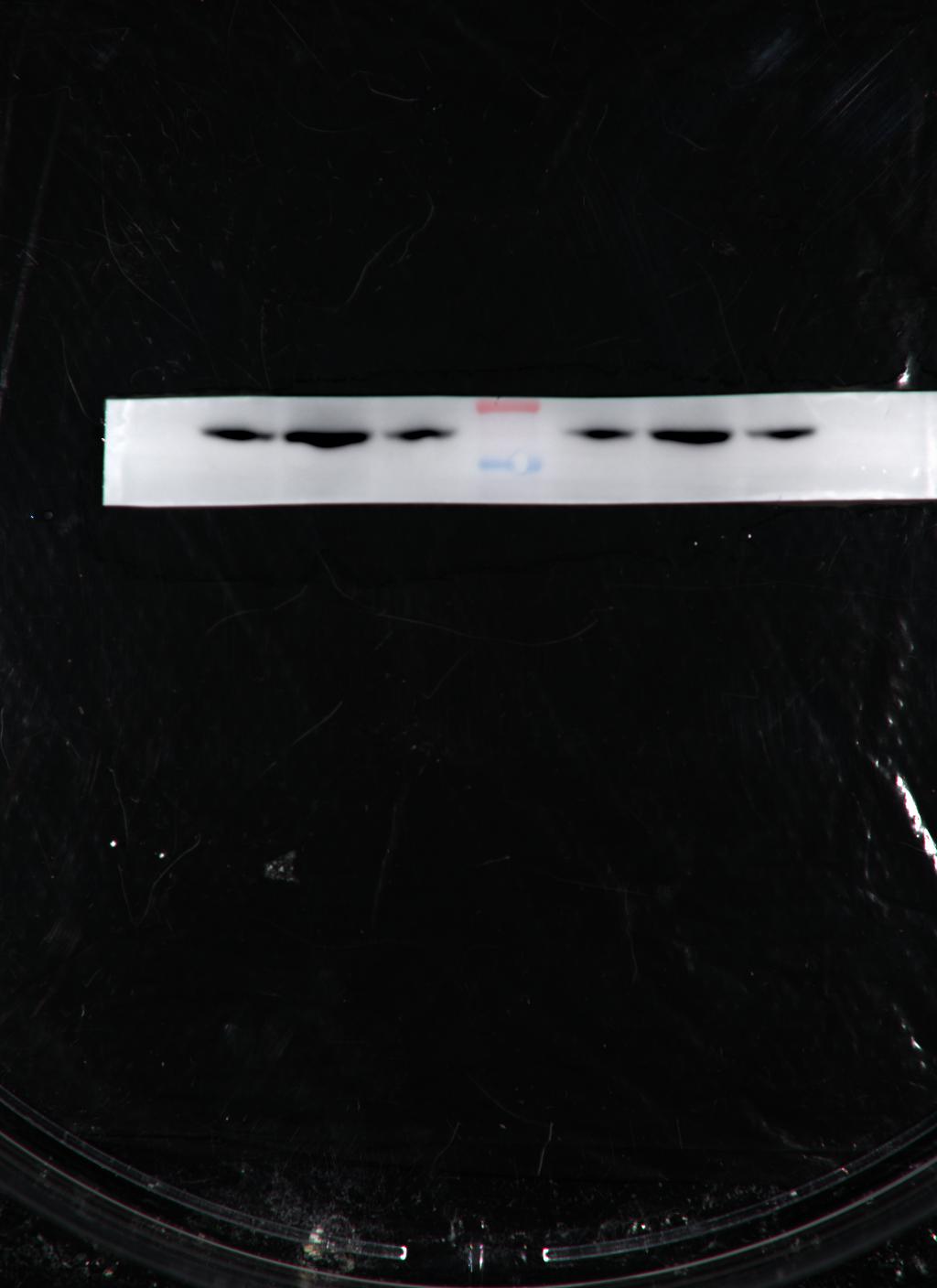

Supplement: Supplementary file 1 [file DataSheet1.zip › animal samples-WB supplementary materials/hippo-p-p65/hippo pp65 5/hippo pp65 +Marker.jpg]

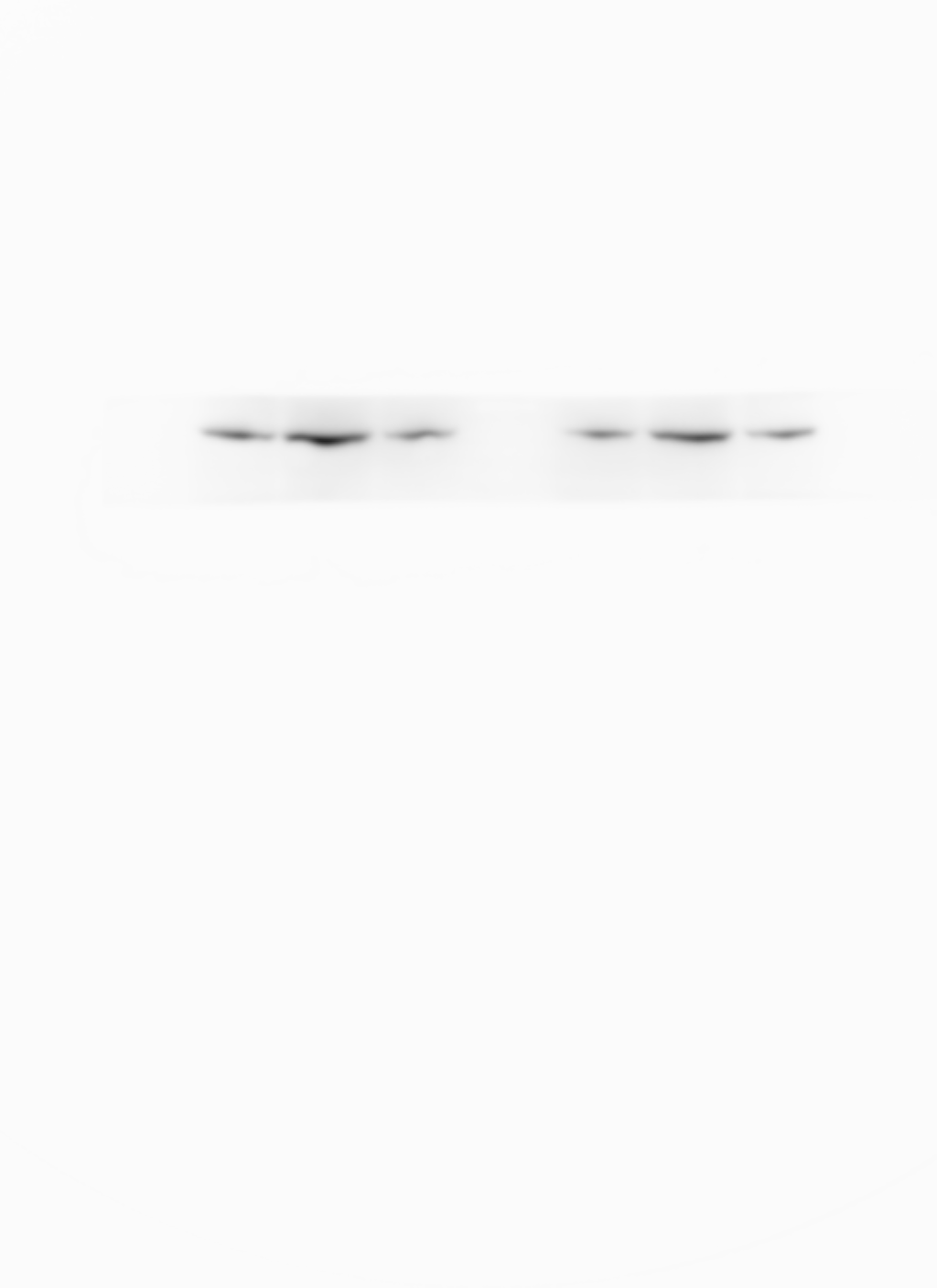

Supplement: Supplementary file 1 [file DataSheet1.zip › animal samples-WB supplementary materials/hippo-p-p65/hippo pp65 5/hippo pp65.tif]

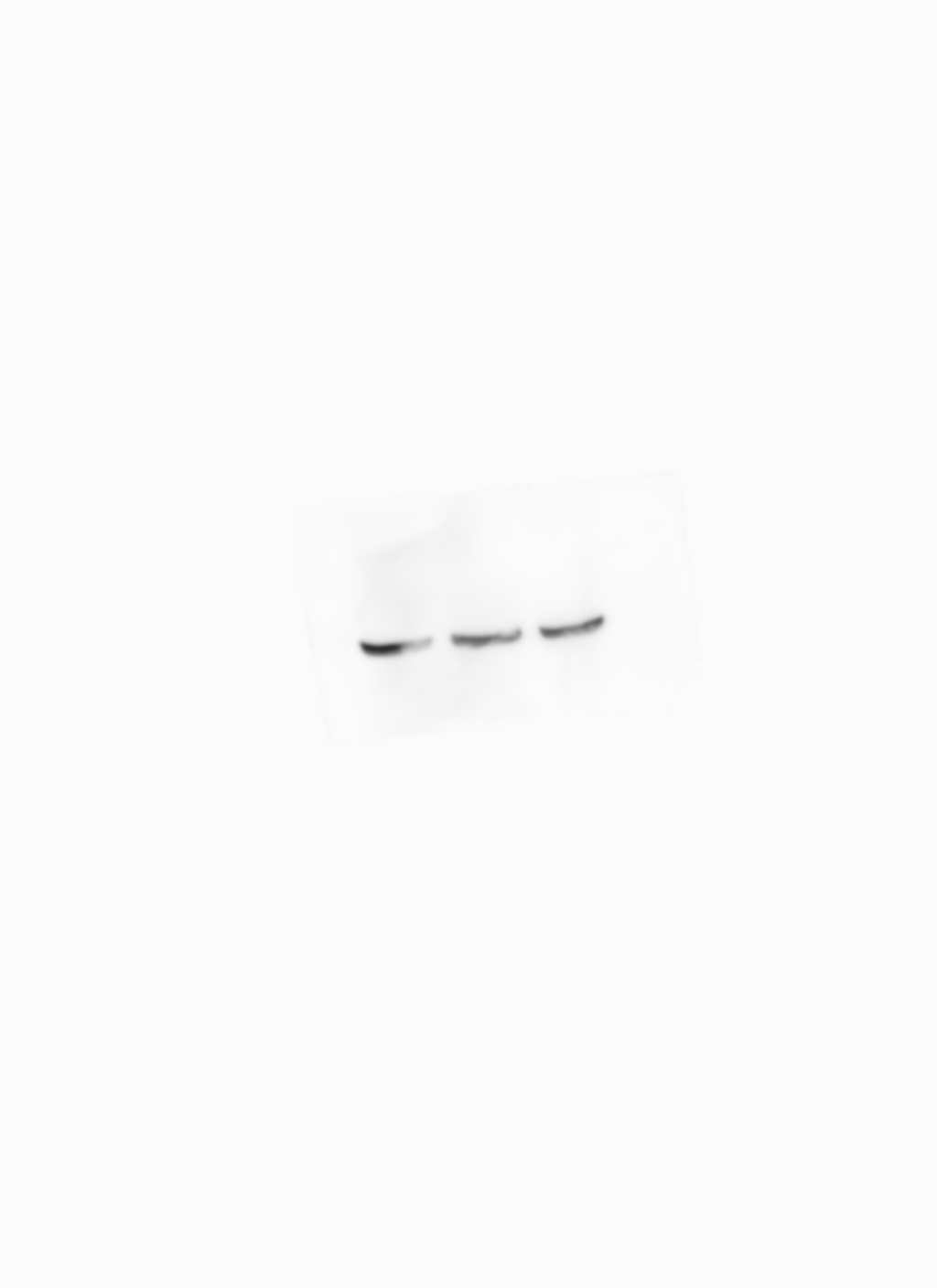

Supplement: Supplementary file 2 [file DataSheet2.zip › cell samples-WB supplementary materials/iNOS/FY b-tubulin 1/FY b-tubulin .tif]

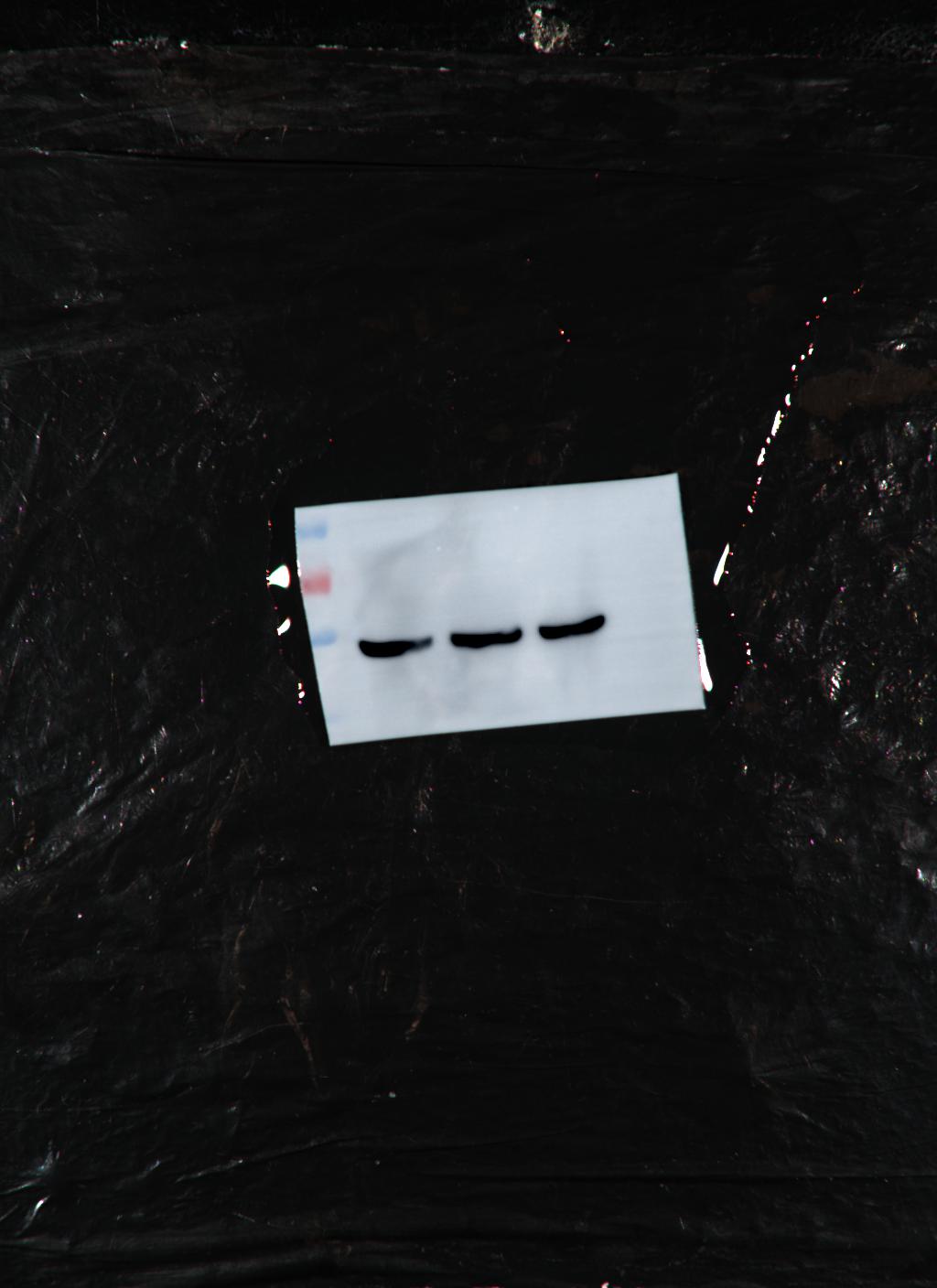

Supplement: Supplementary file 2 [file DataSheet2.zip › cell samples-WB supplementary materials/iNOS/FY b-tubulin 1/FY b-tubulin +Marker.jpg]

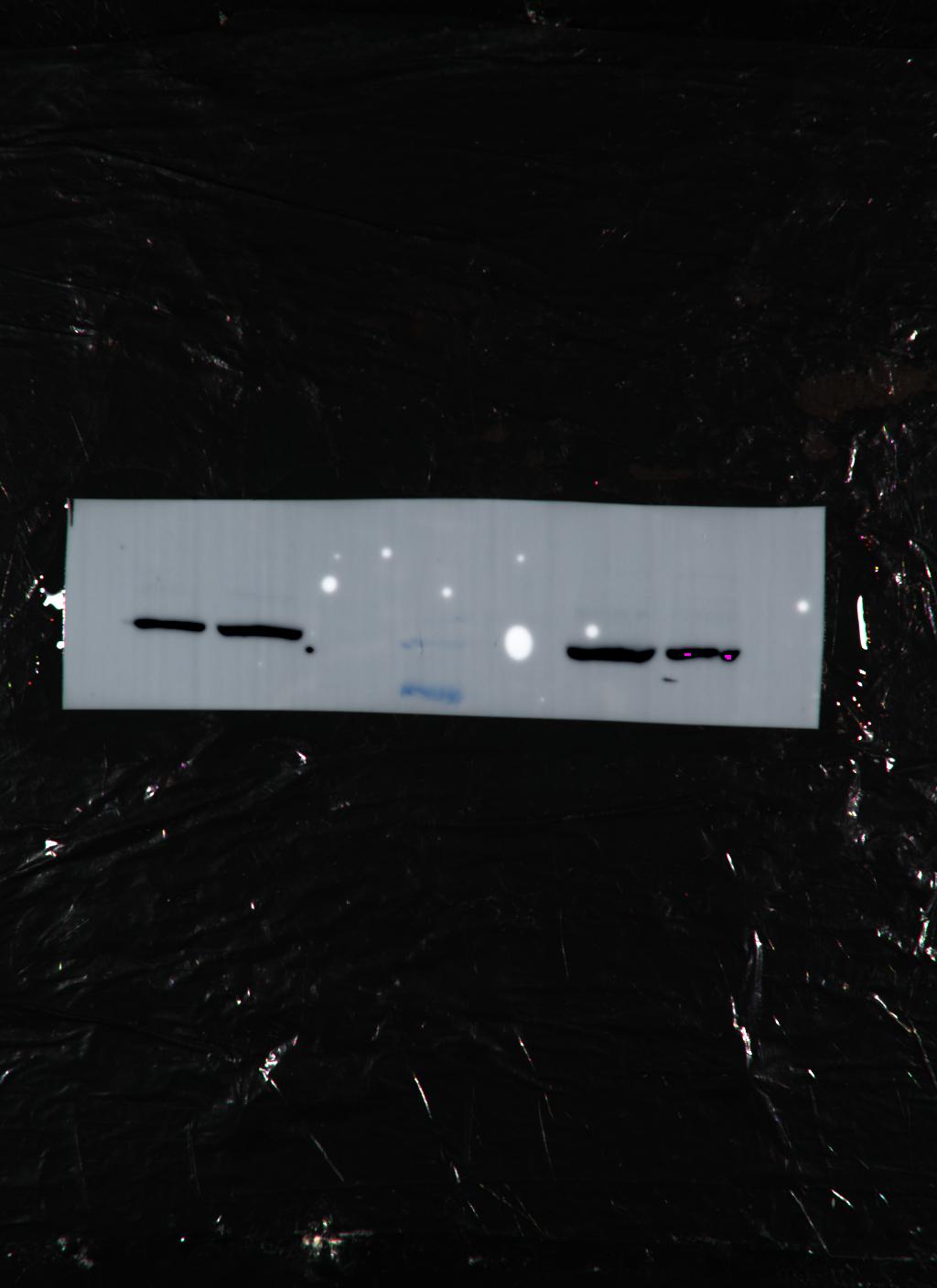

Supplement: Supplementary file 2 [file DataSheet2.zip › cell samples-WB supplementary materials/iNOS/FY inos 1/FY inos +Marker.jpg]

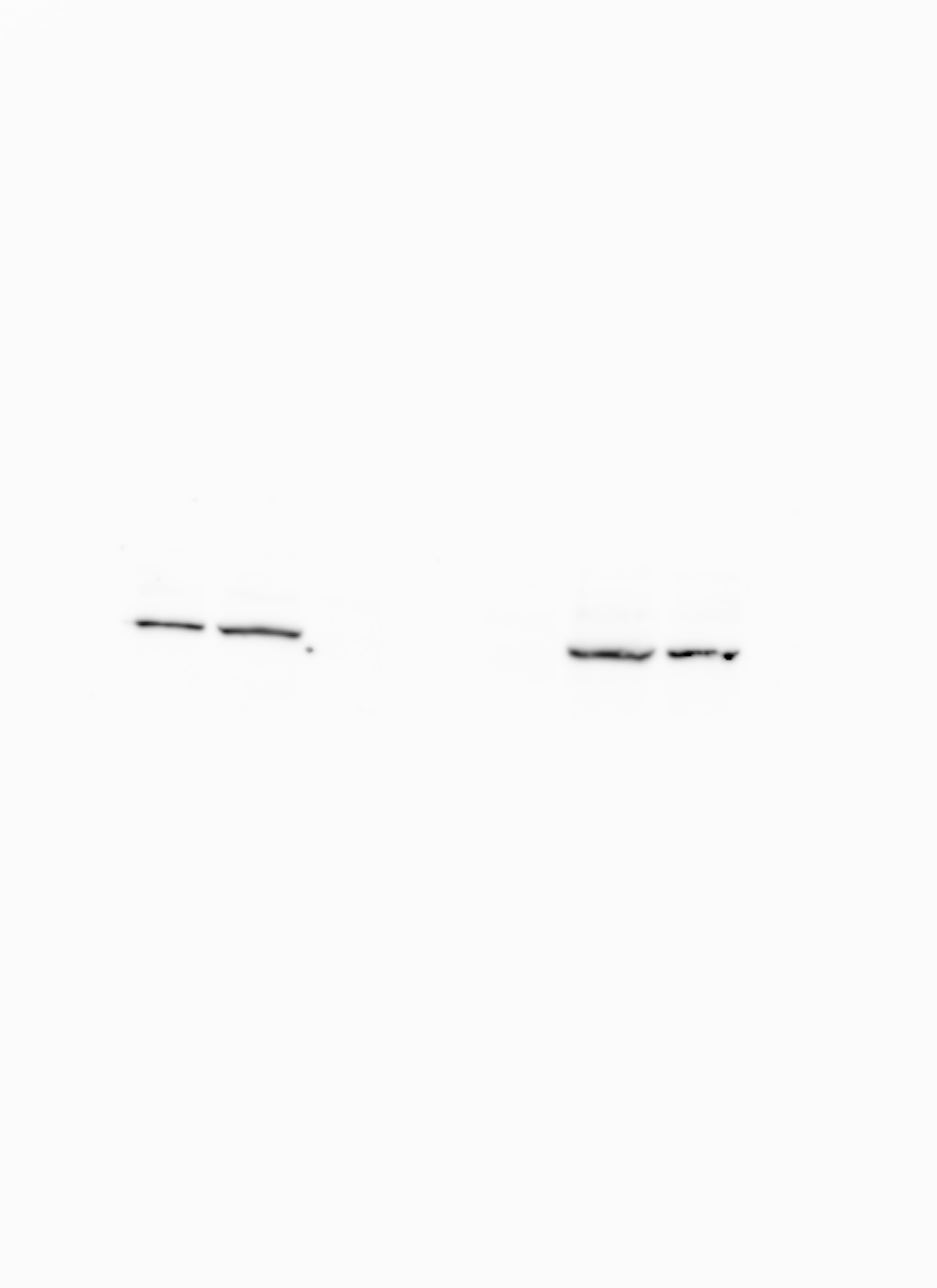

Supplement: Supplementary file 2 [file DataSheet2.zip › cell samples-WB supplementary materials/iNOS/FY inos 1/FY inos .tif]

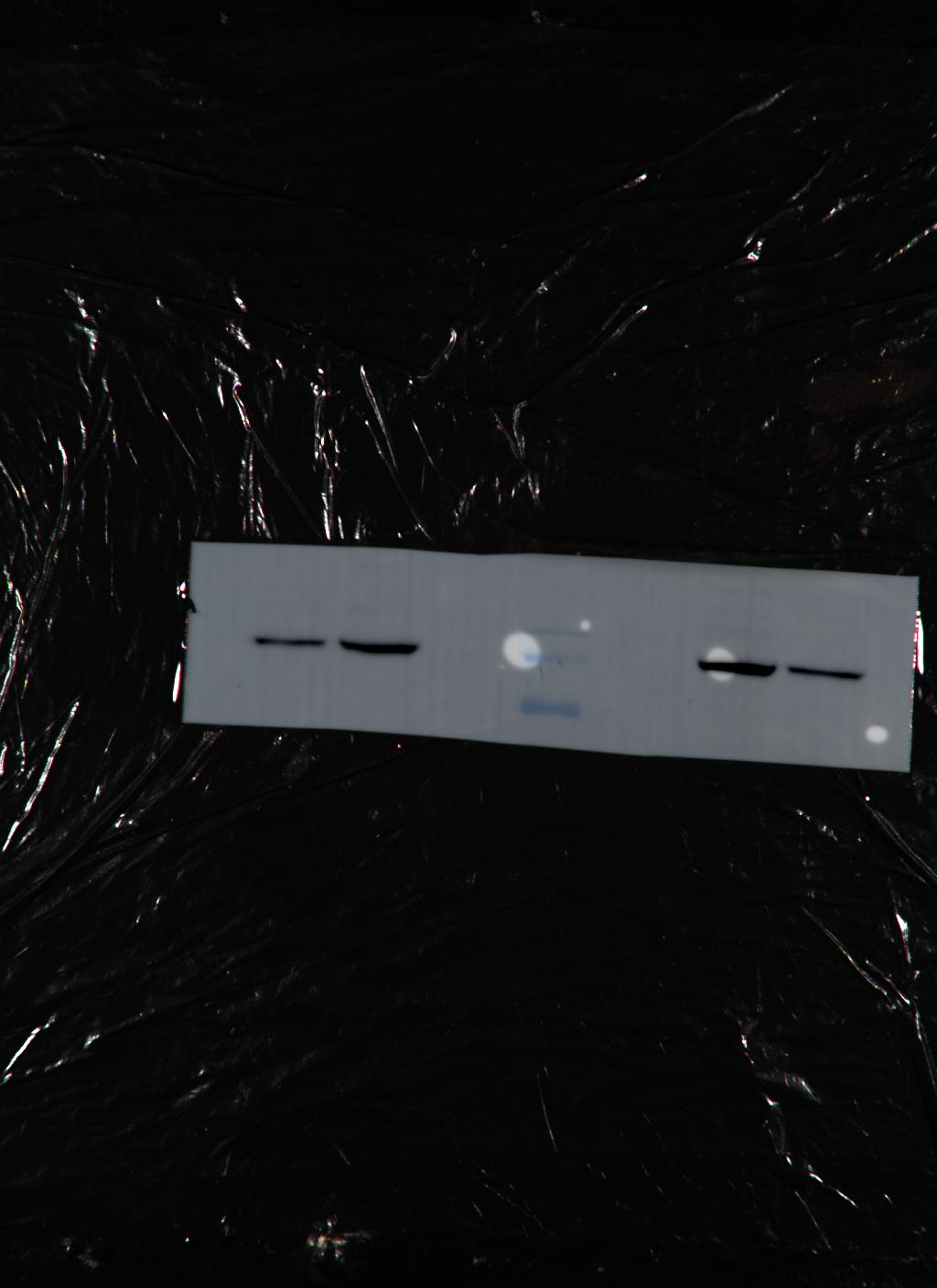

Supplement: Supplementary file 2 [file DataSheet2.zip › cell samples-WB supplementary materials/iNOS/FY inos 2/FY inos +Marker.jpg]

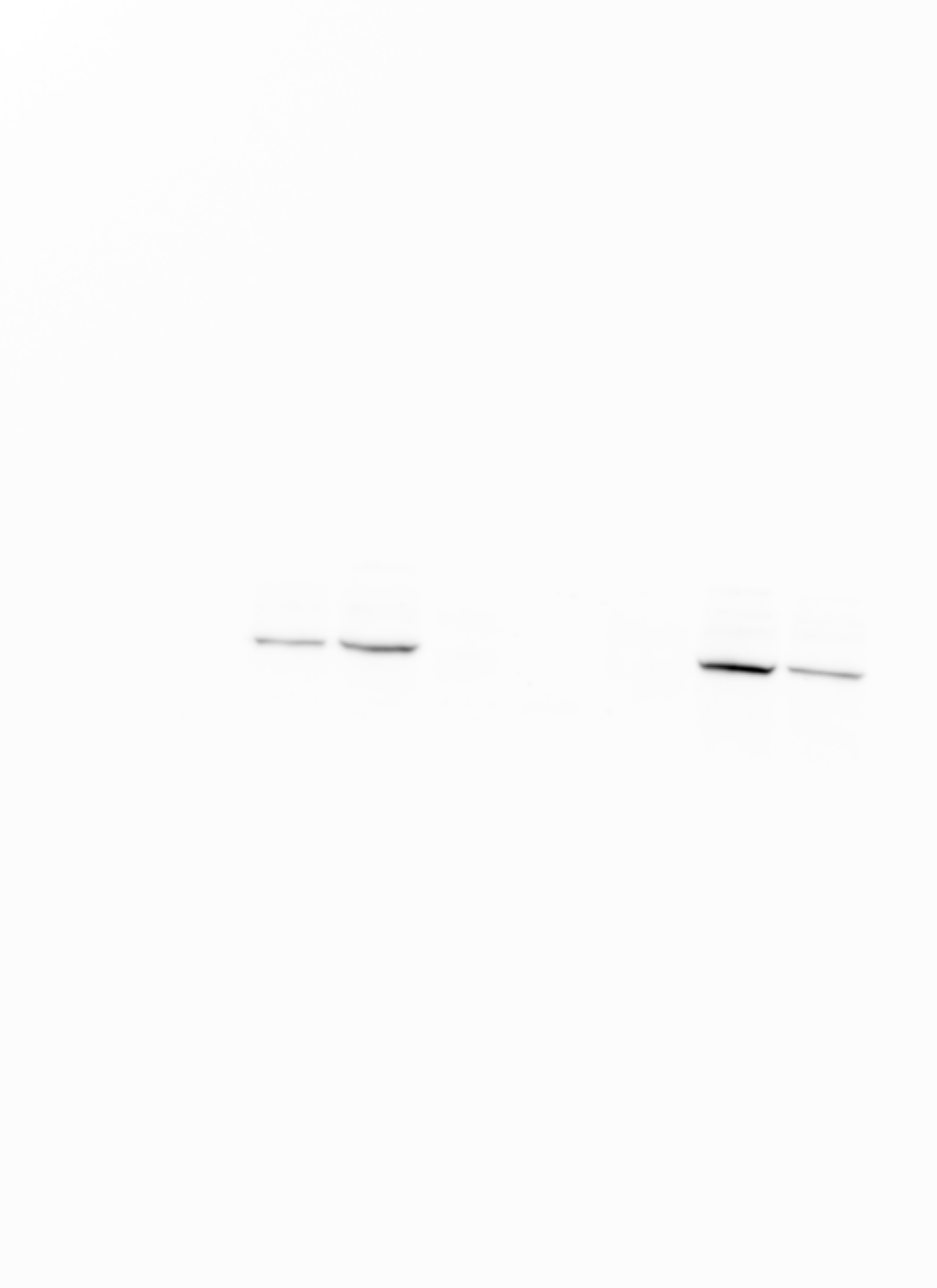

Supplement: Supplementary file 2 [file DataSheet2.zip › cell samples-WB supplementary materials/iNOS/FY inos 2/FY inos .tif]

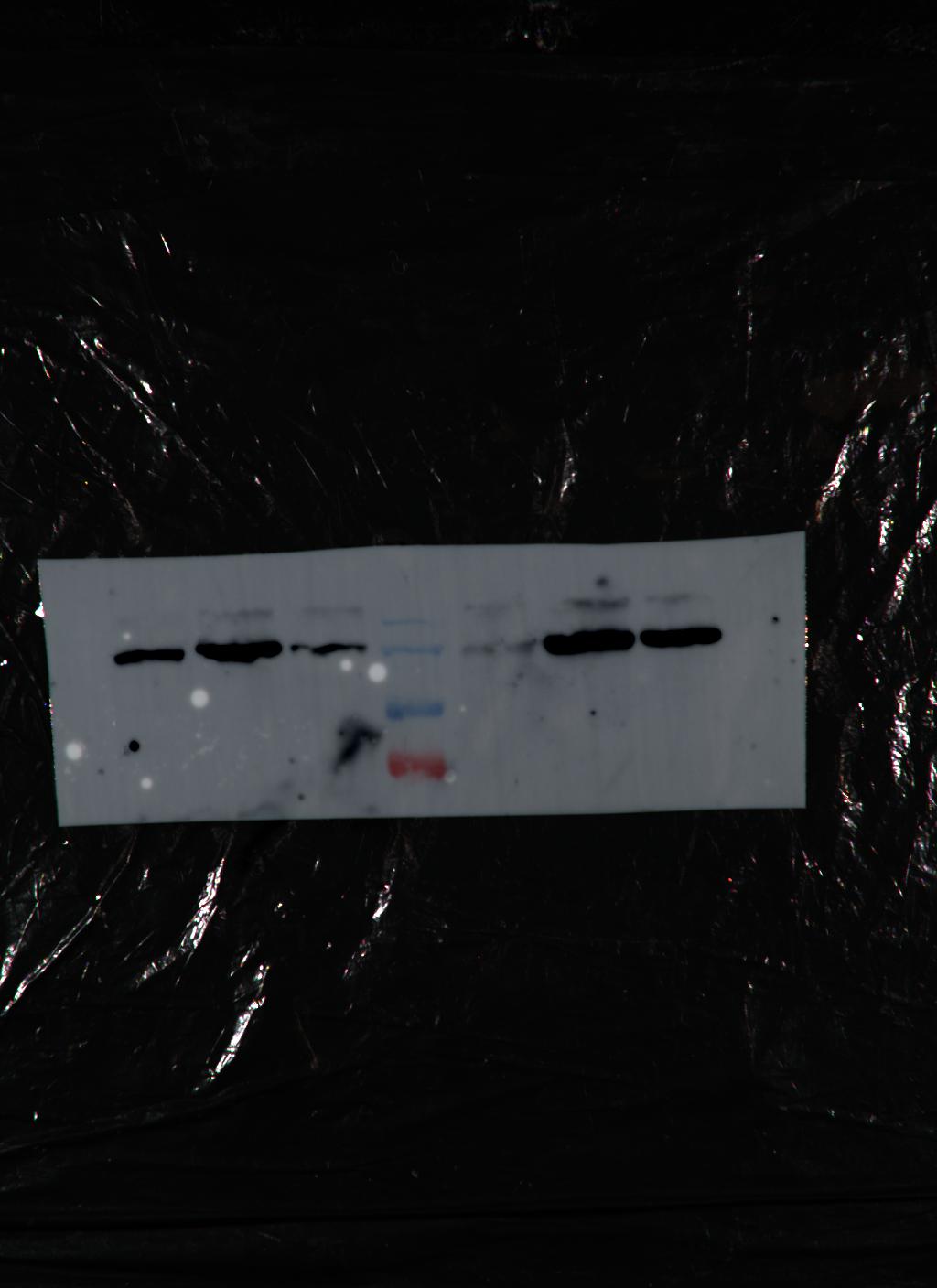

Supplement: Supplementary file 2 [file DataSheet2.zip › cell samples-WB supplementary materials/iNOS/FY iNOS 3/FY iNOS +Marker.jpg]

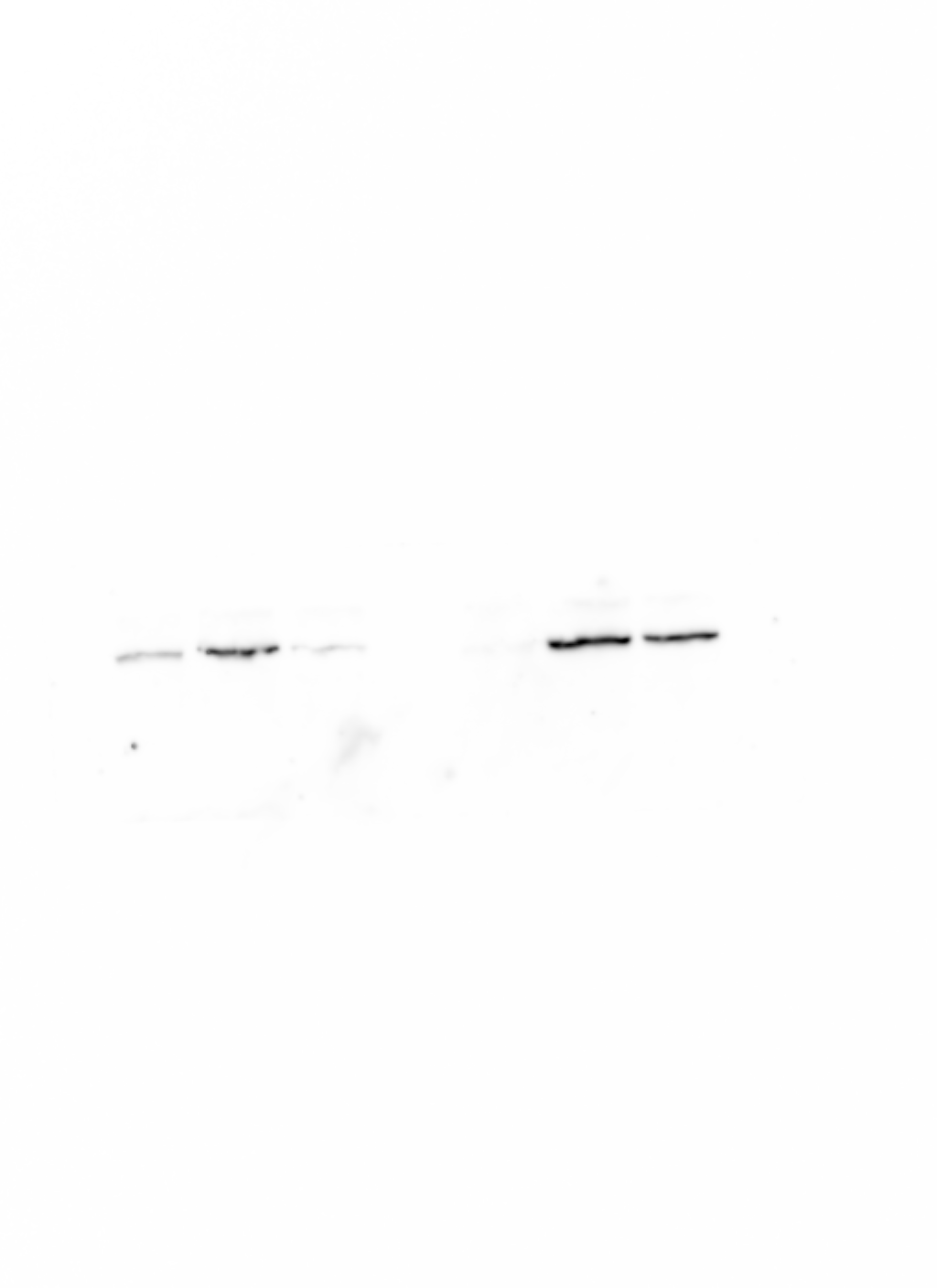

Supplement: Supplementary file 2 [file DataSheet2.zip › cell samples-WB supplementary materials/iNOS/FY iNOS 3/FY iNOS .tif]

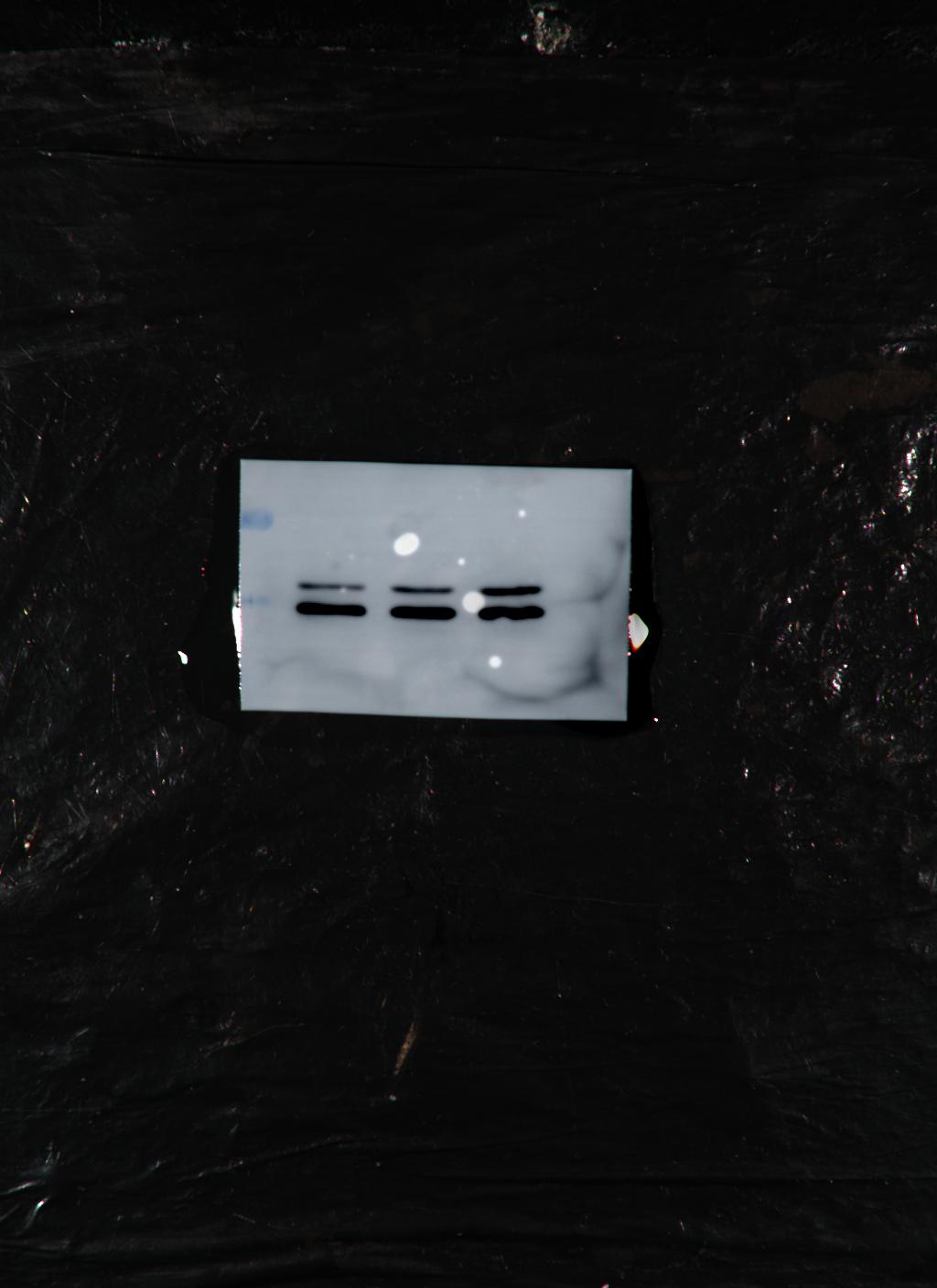

Supplement: Supplementary file 2 [file DataSheet2.zip › cell samples-WB supplementary materials/p-Erk/erk 1/erk +Marker.jpg]

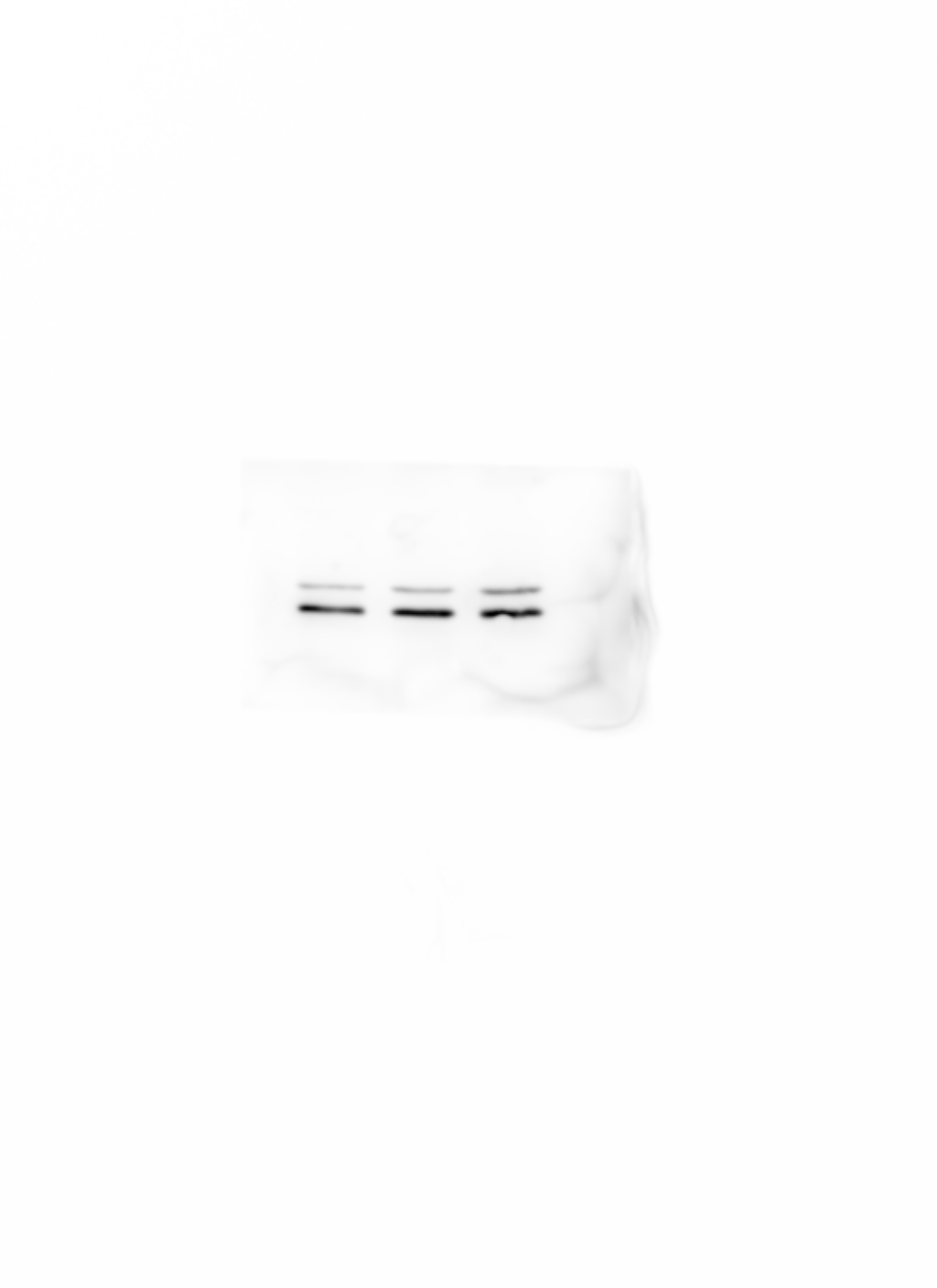

Supplement: Supplementary file 2 [file DataSheet2.zip › cell samples-WB supplementary materials/p-Erk/erk 1/erk .tif]

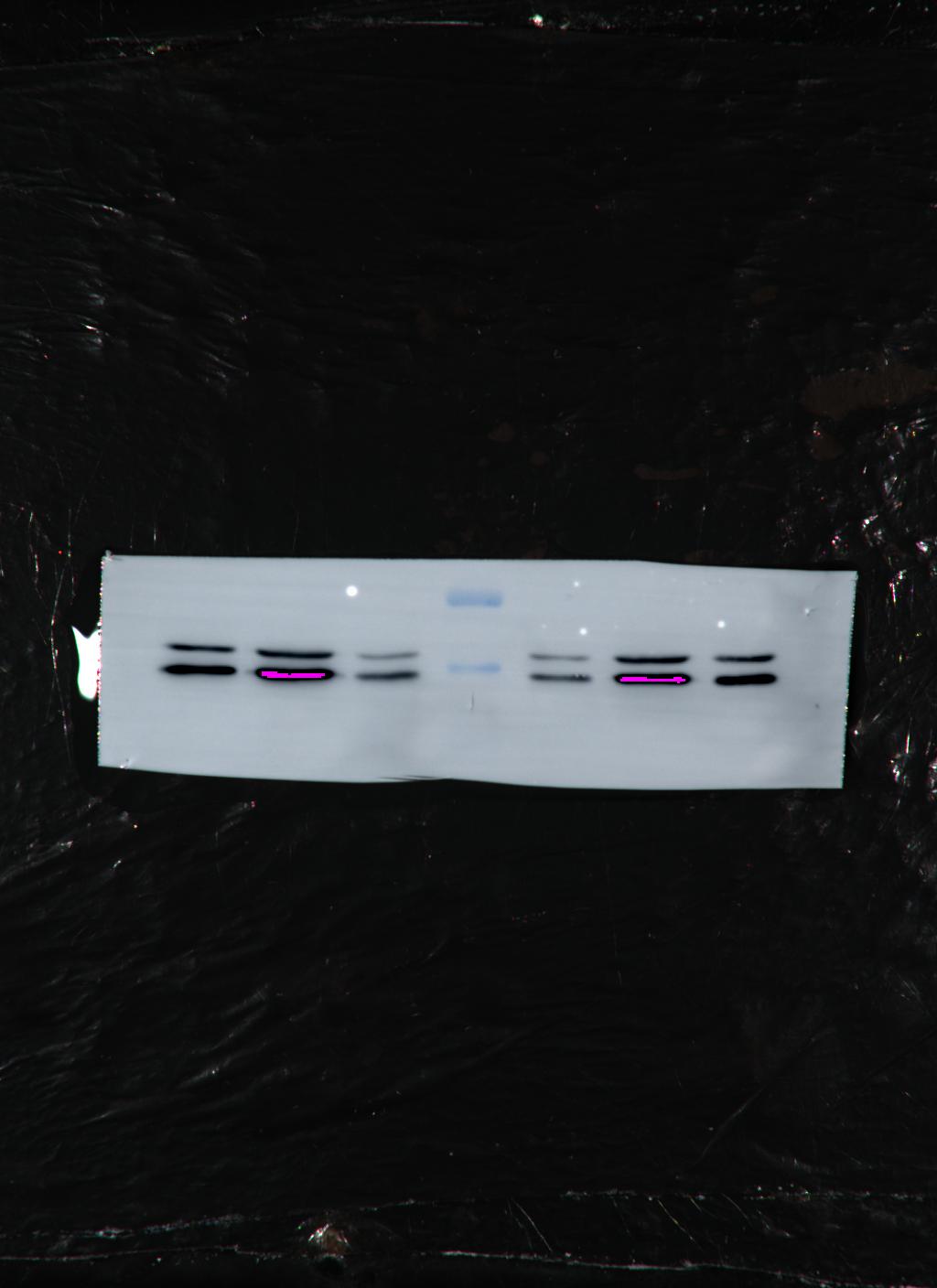

Supplement: Supplementary file 2 [file DataSheet2.zip › cell samples-WB supplementary materials/p-Erk/P-erk 1/P-erk +Marker.jpg]

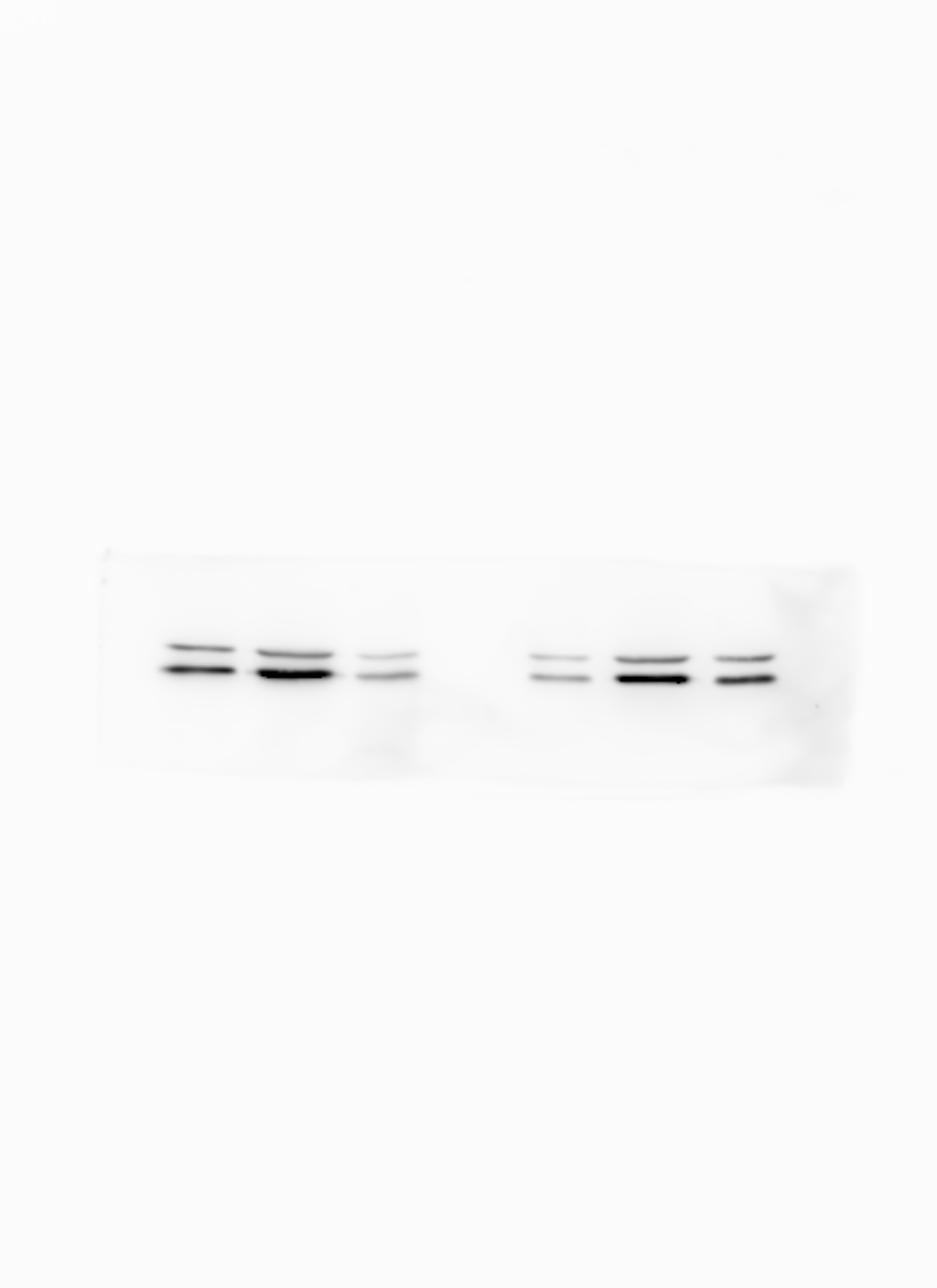

Supplement: Supplementary file 2 [file DataSheet2.zip › cell samples-WB supplementary materials/p-Erk/P-erk 1/P-erk .tif]

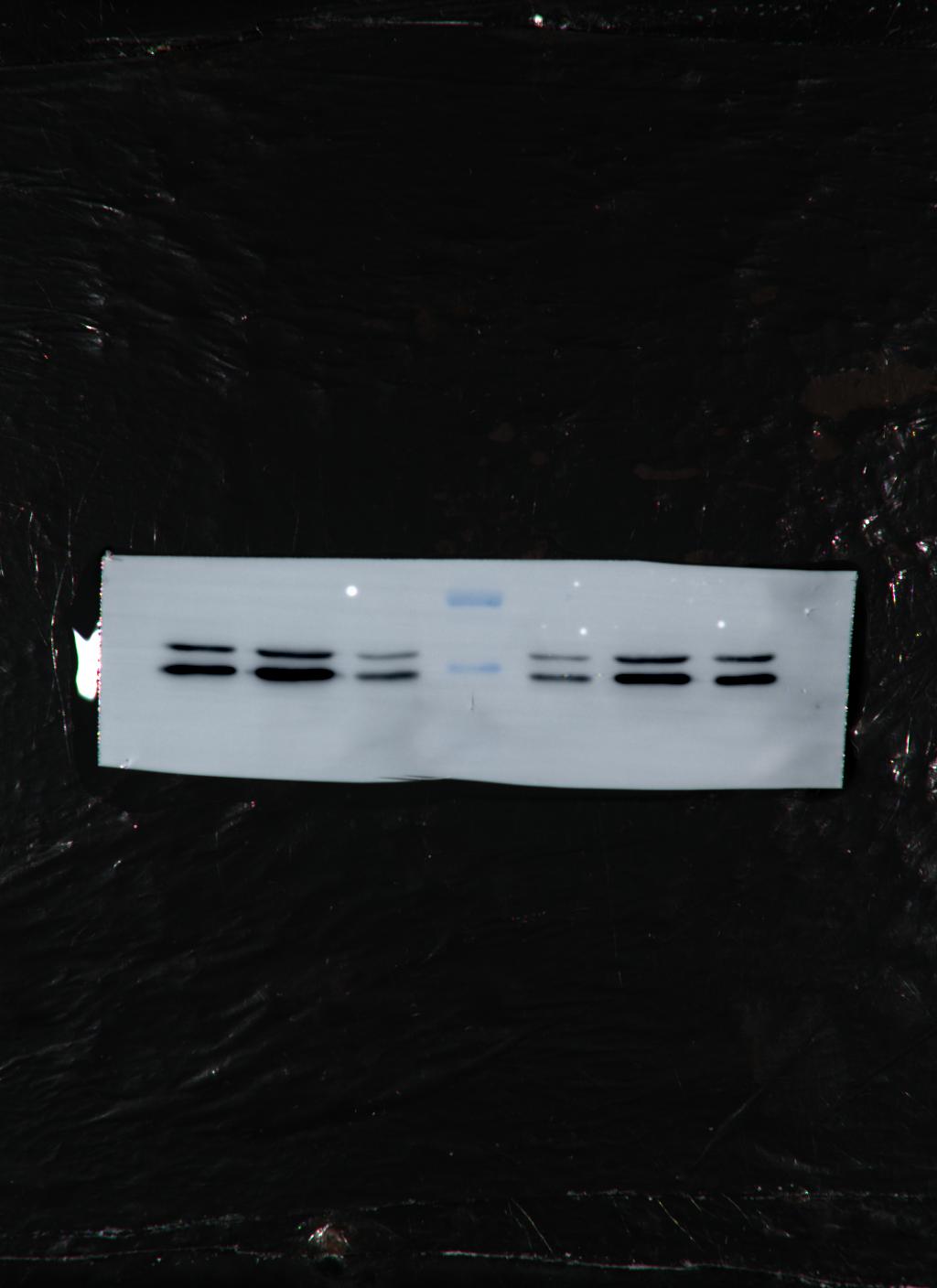

Supplement: Supplementary file 2 [file DataSheet2.zip › cell samples-WB supplementary materials/p-Erk/P-erk 2/P-erk +Marker.jpg]

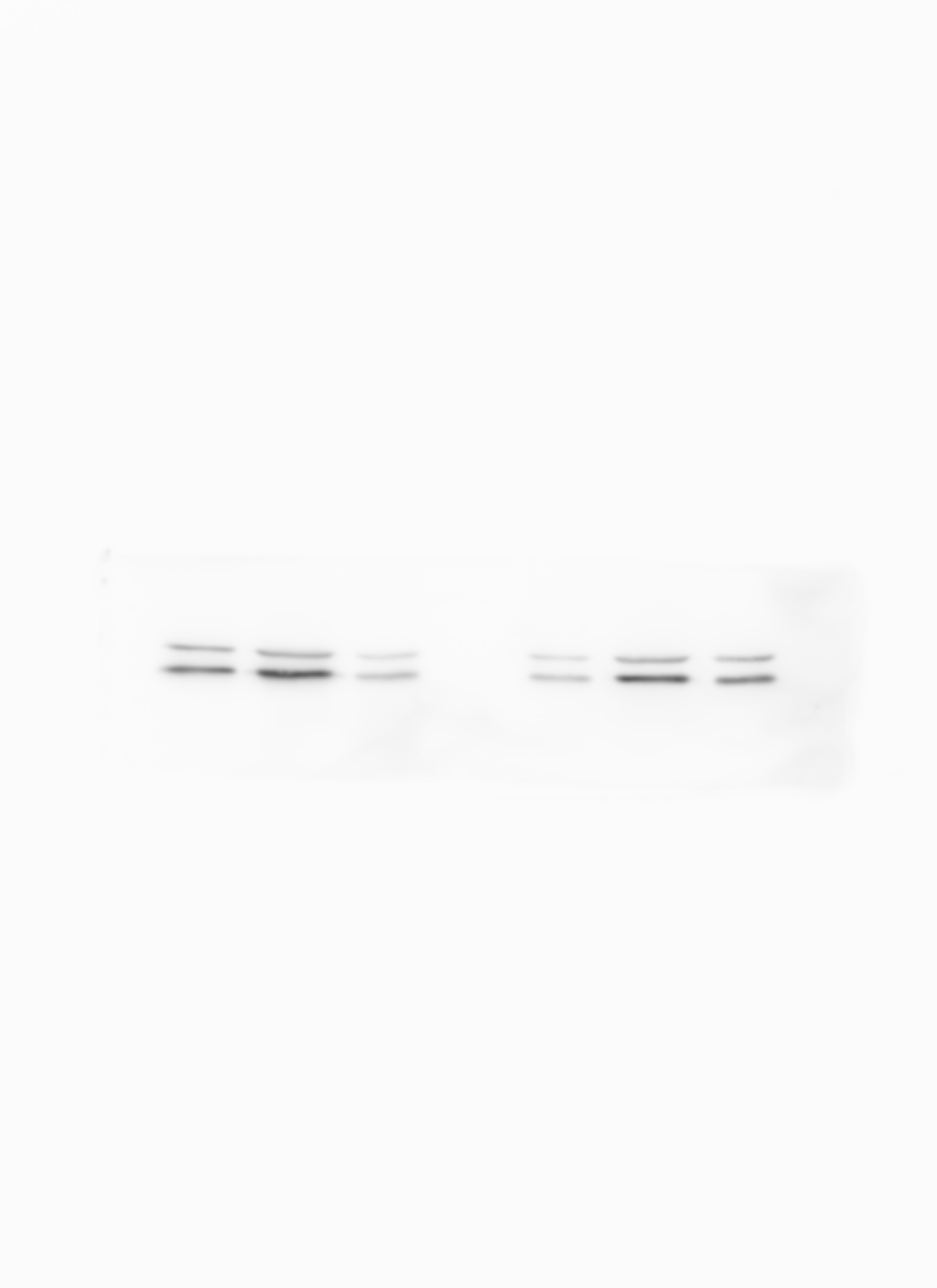

Supplement: Supplementary file 2 [file DataSheet2.zip › cell samples-WB supplementary materials/p-Erk/P-erk 2/P-erk .tif]

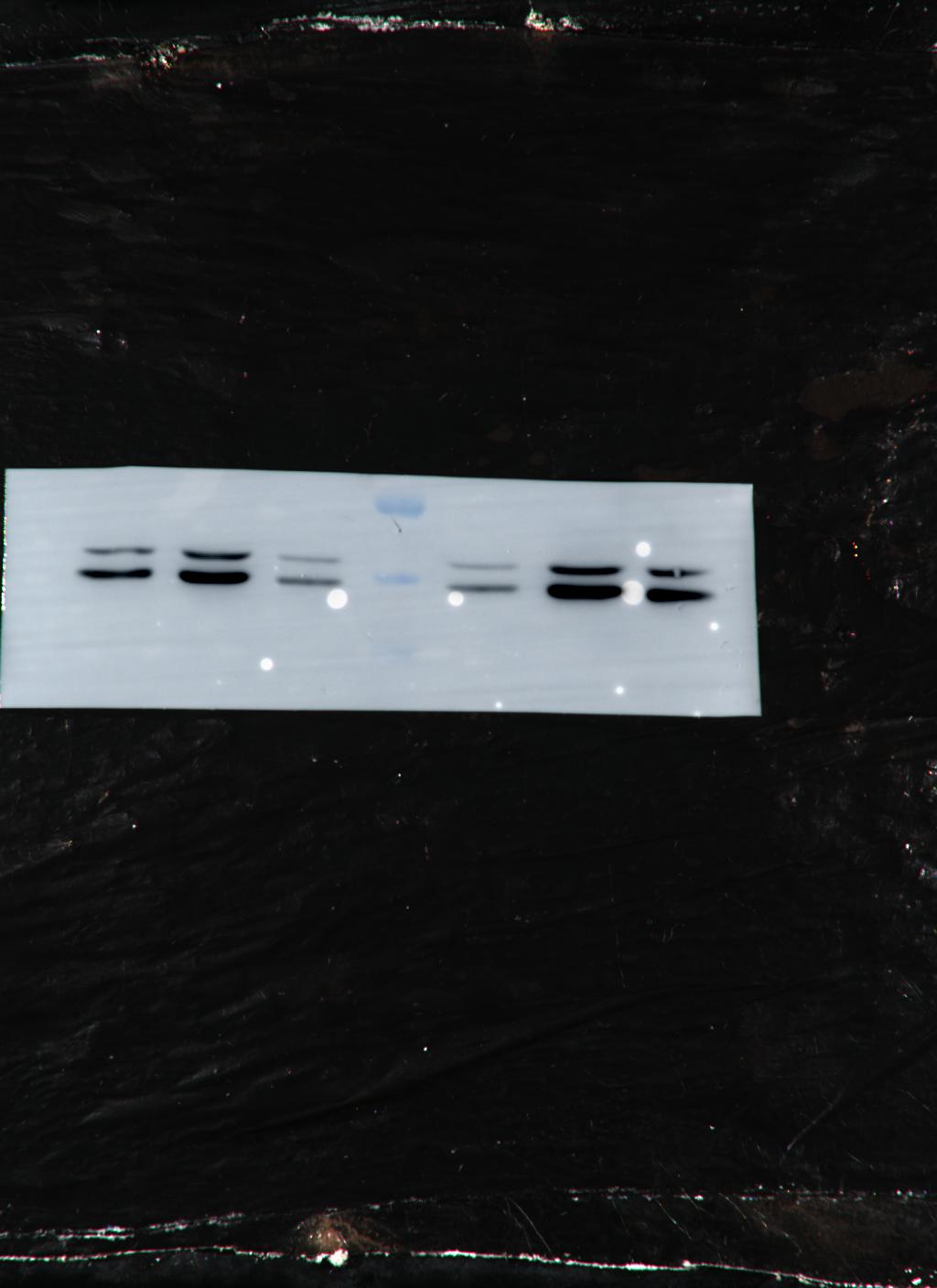

Supplement: Supplementary file 2 [file DataSheet2.zip › cell samples-WB supplementary materials/p-Erk/P-erk 3/P-erk +Marker.jpg]

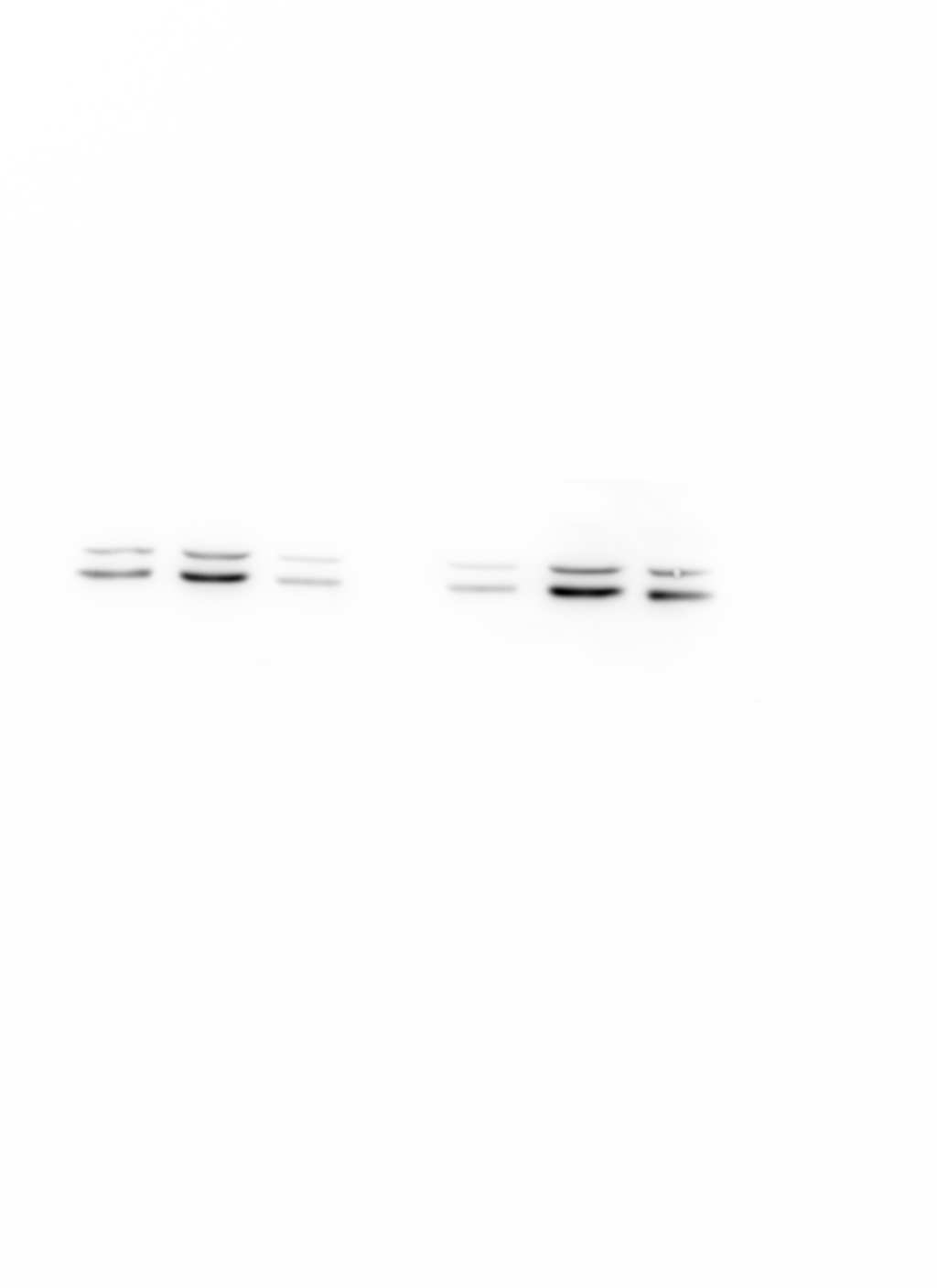

Supplement: Supplementary file 2 [file DataSheet2.zip › cell samples-WB supplementary materials/p-Erk/P-erk 3/P-erk .tif]

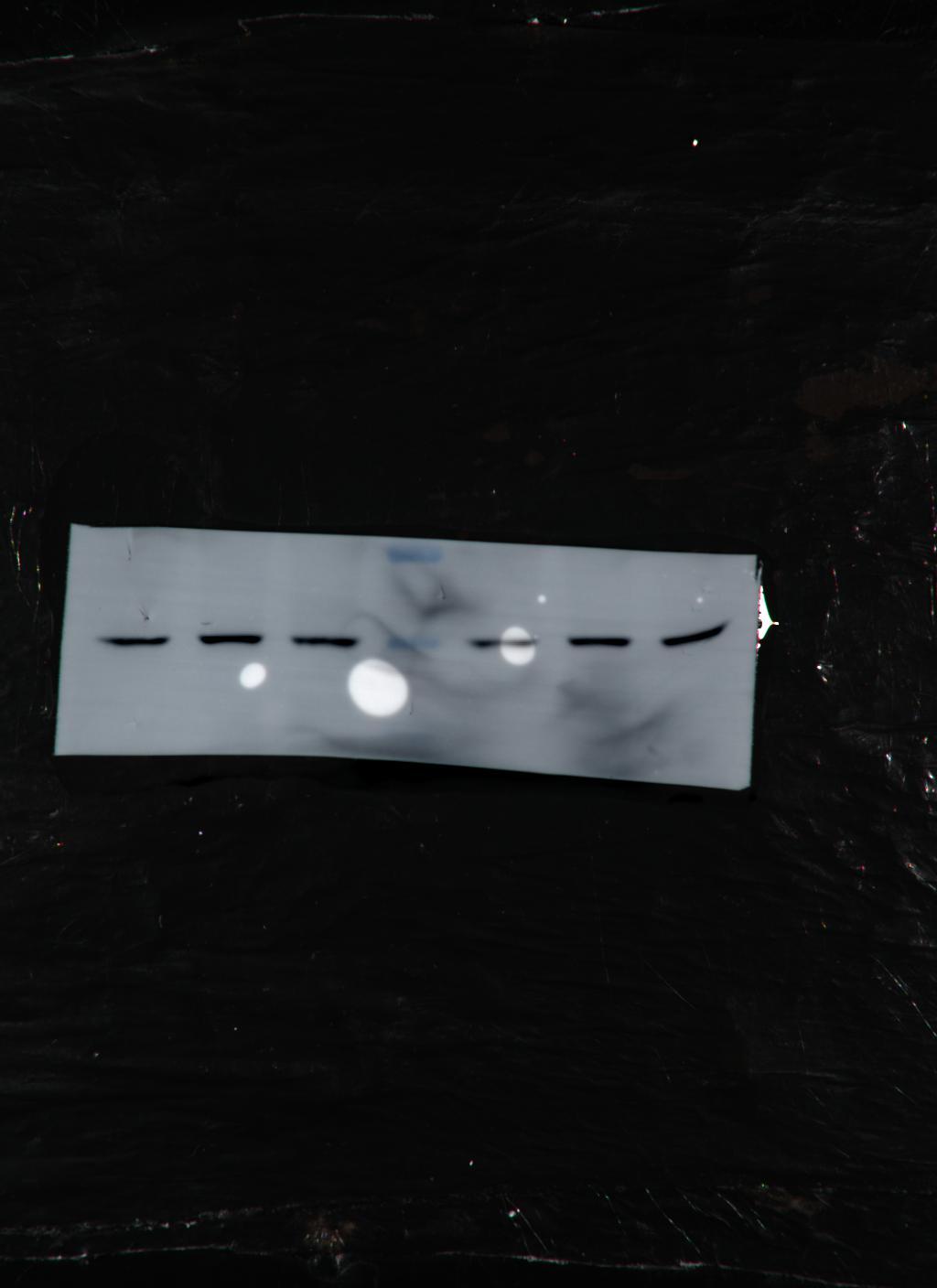

Supplement: Supplementary file 2 [file DataSheet2.zip › cell samples-WB supplementary materials/p-p38/P-P38 1/P-P38 +Marker.jpg]

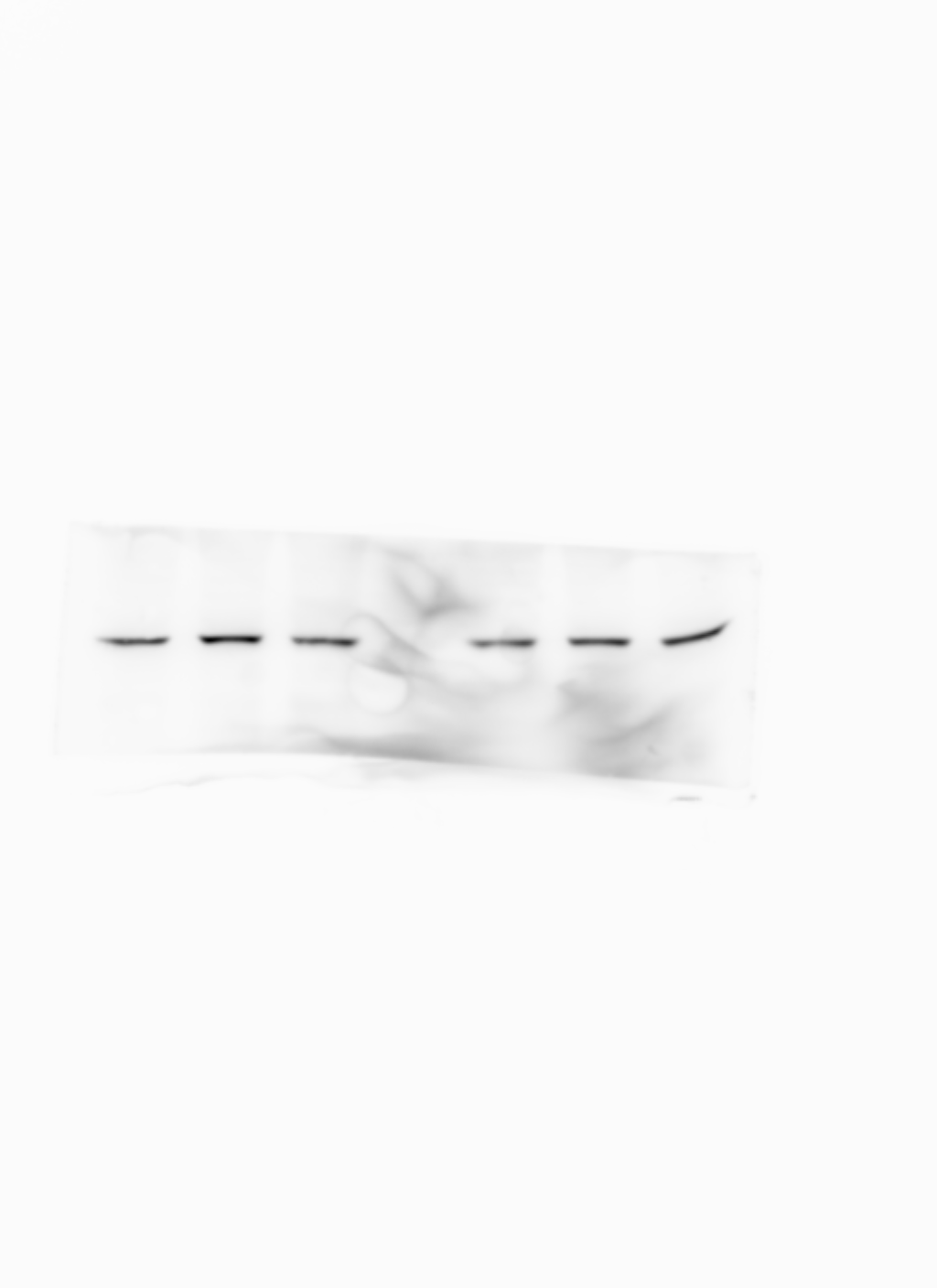

Supplement: Supplementary file 2 [file DataSheet2.zip › cell samples-WB supplementary materials/p-p38/P-P38 1/P-P38 .tif]

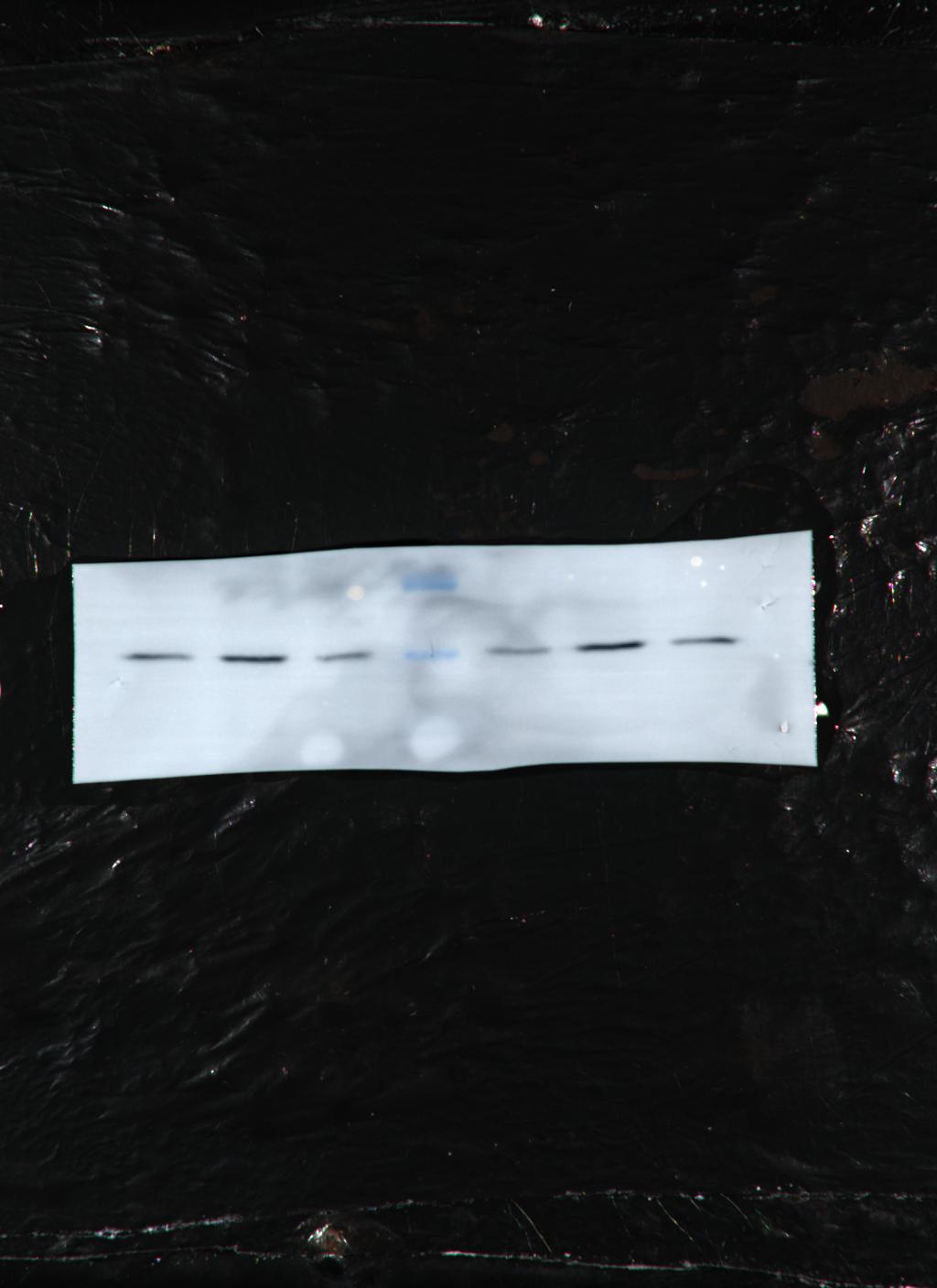

Supplement: Supplementary file 2 [file DataSheet2.zip › cell samples-WB supplementary materials/p-p38/P-p38 2/P-p38 +Marker.jpg]

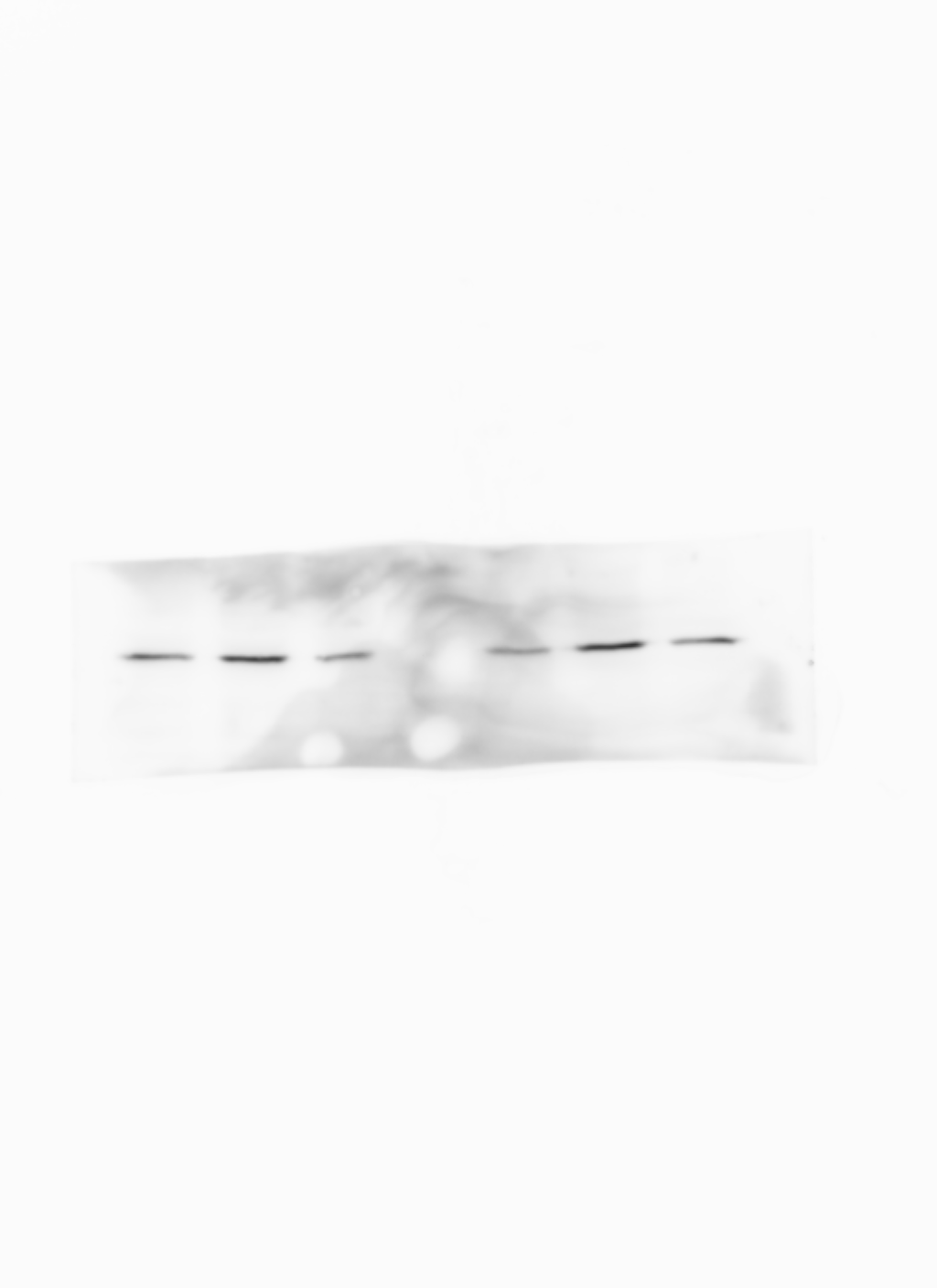

Supplement: Supplementary file 2 [file DataSheet2.zip › cell samples-WB supplementary materials/p-p38/P-p38 2/P-p38 .tif]

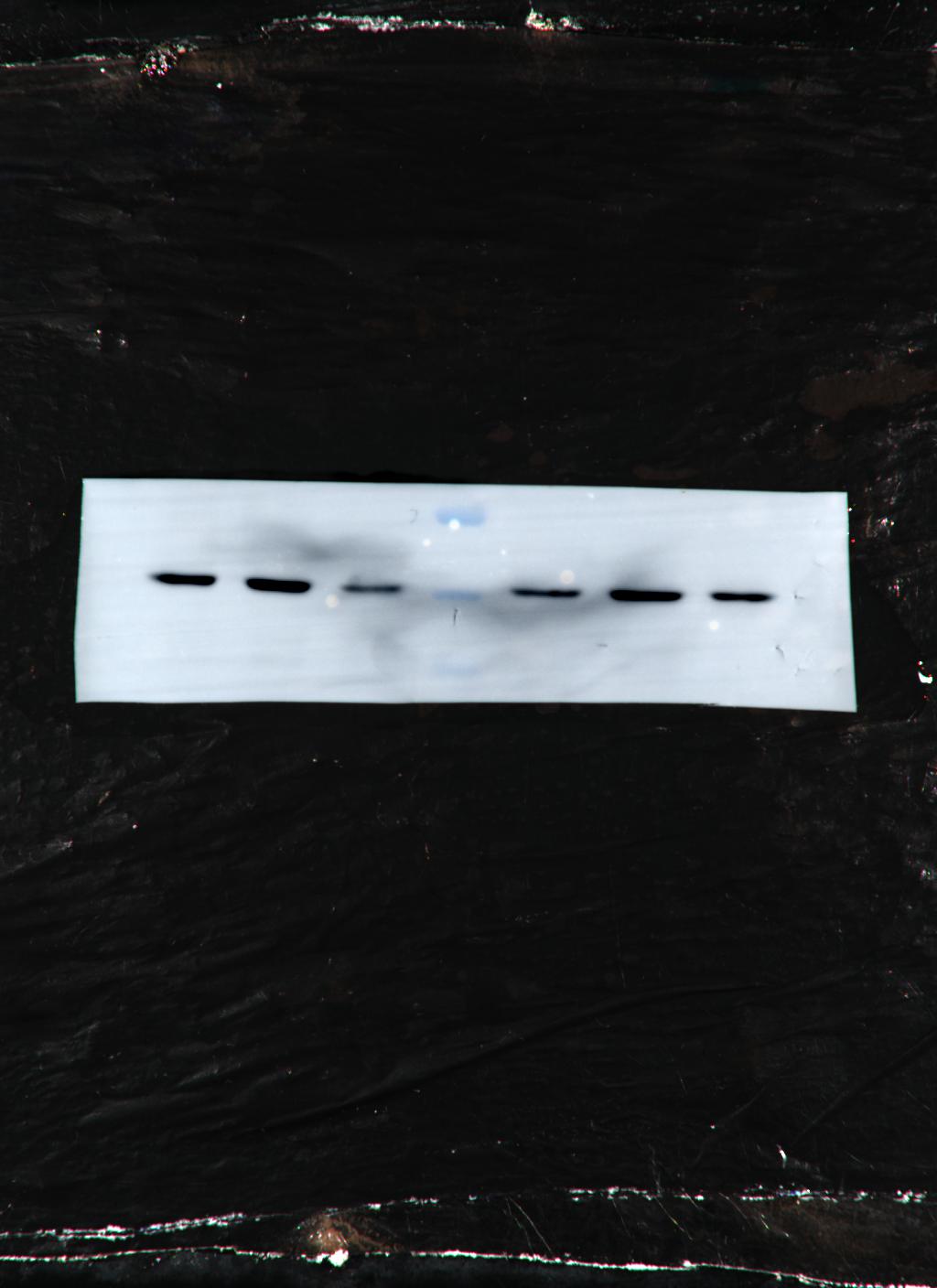

Supplement: Supplementary file 2 [file DataSheet2.zip › cell samples-WB supplementary materials/p-p38/P-p38 3/P-p38 +Marker.jpg]

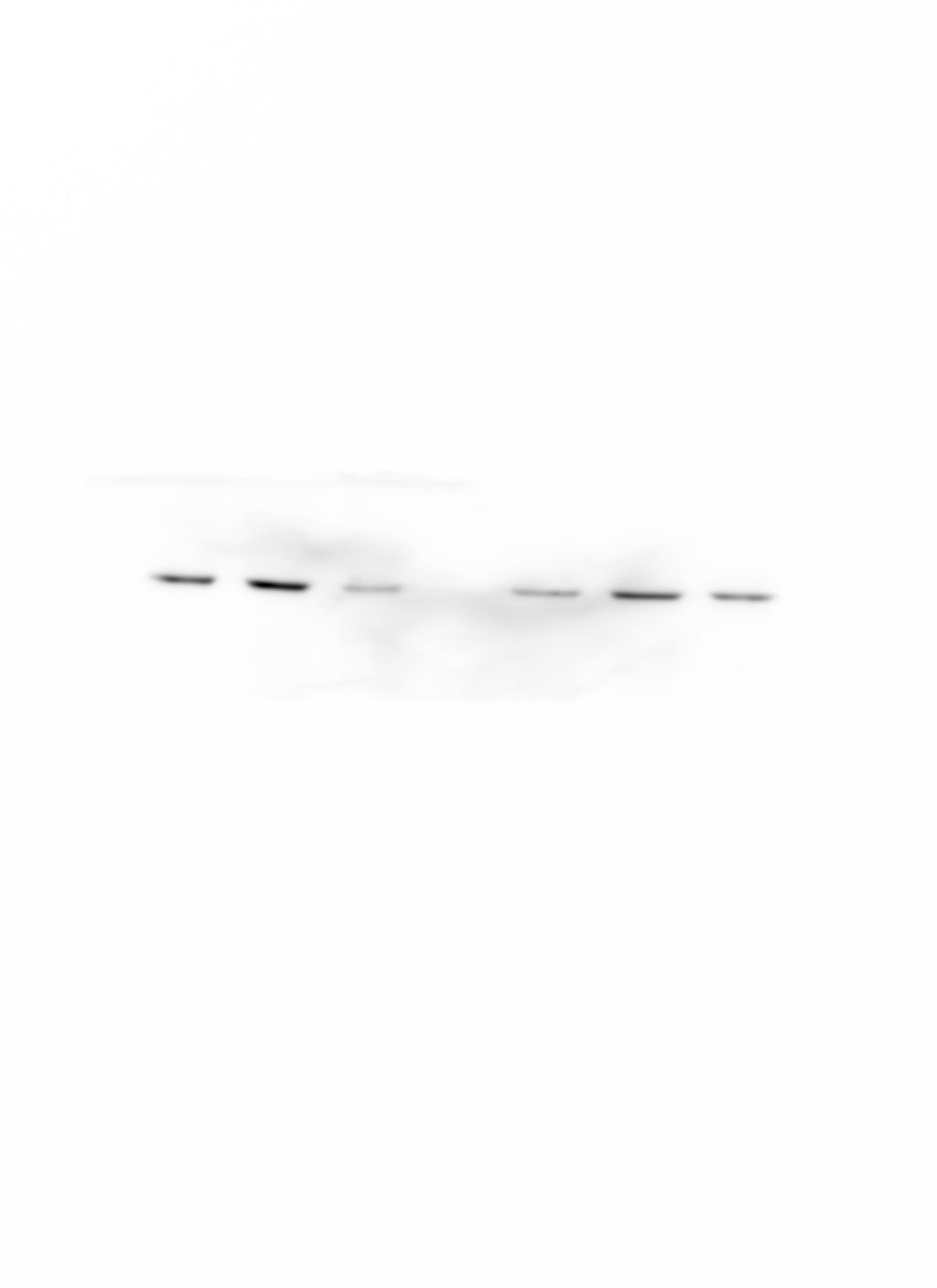

Supplement: Supplementary file 2 [file DataSheet2.zip › cell samples-WB supplementary materials/p-p38/P-p38 3/P-p38 .tif]

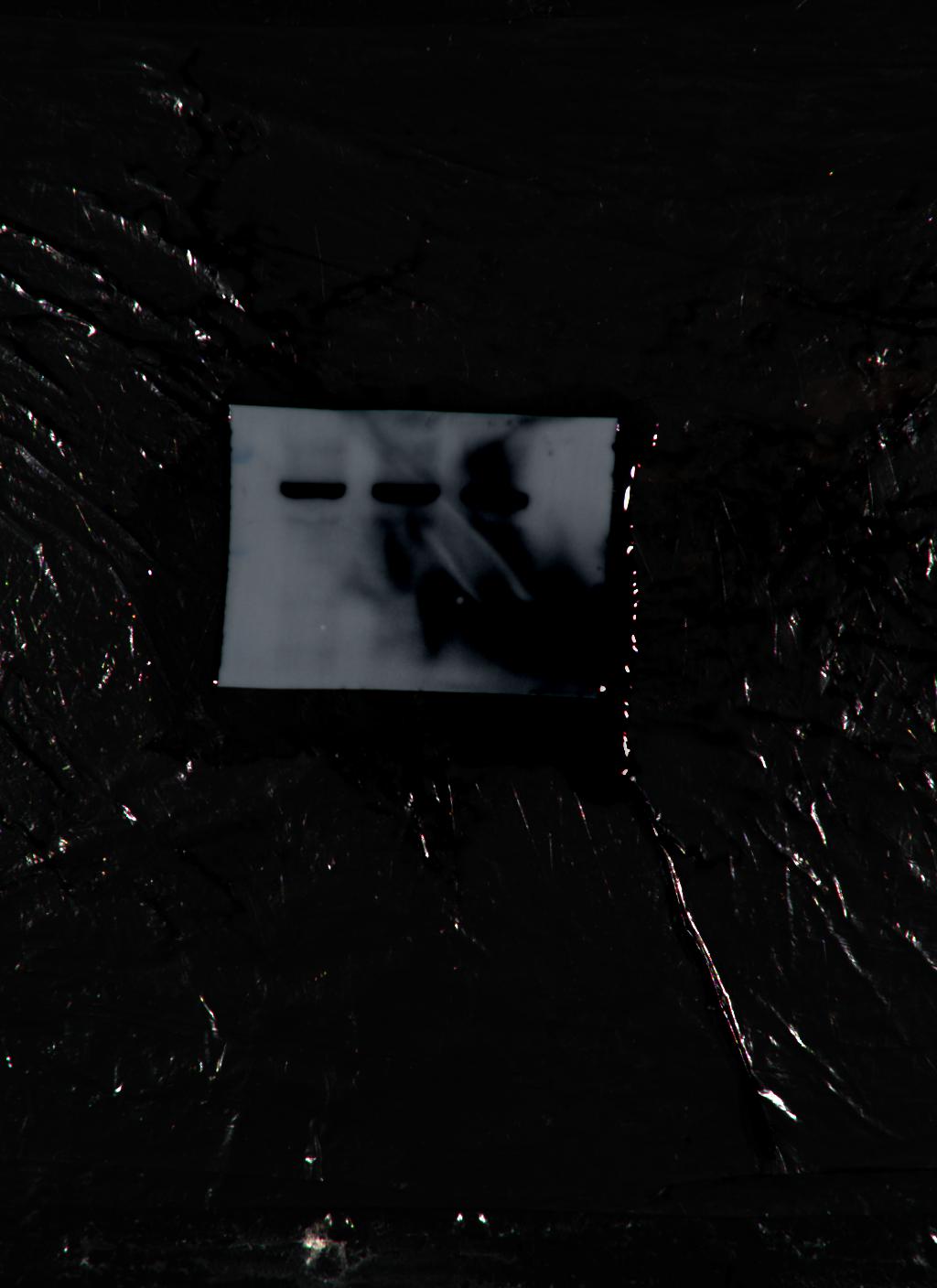

Supplement: Supplementary file 2 [file DataSheet2.zip › cell samples-WB supplementary materials/p-p38/P38 1/P38 +Marker.jpg]

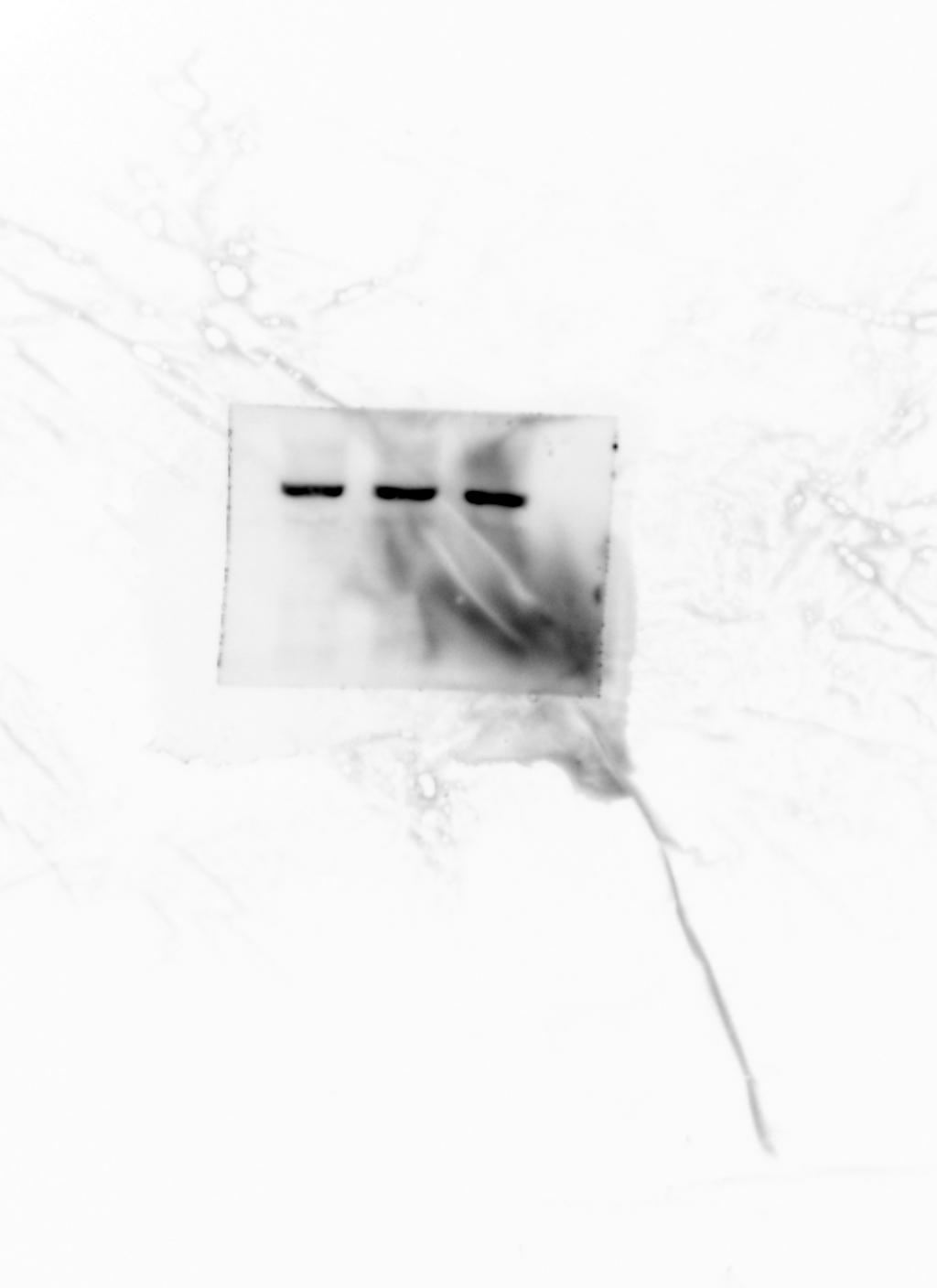

Supplement: Supplementary file 2 [file DataSheet2.zip › cell samples-WB supplementary materials/p-p38/P38 1/P38 .tif]

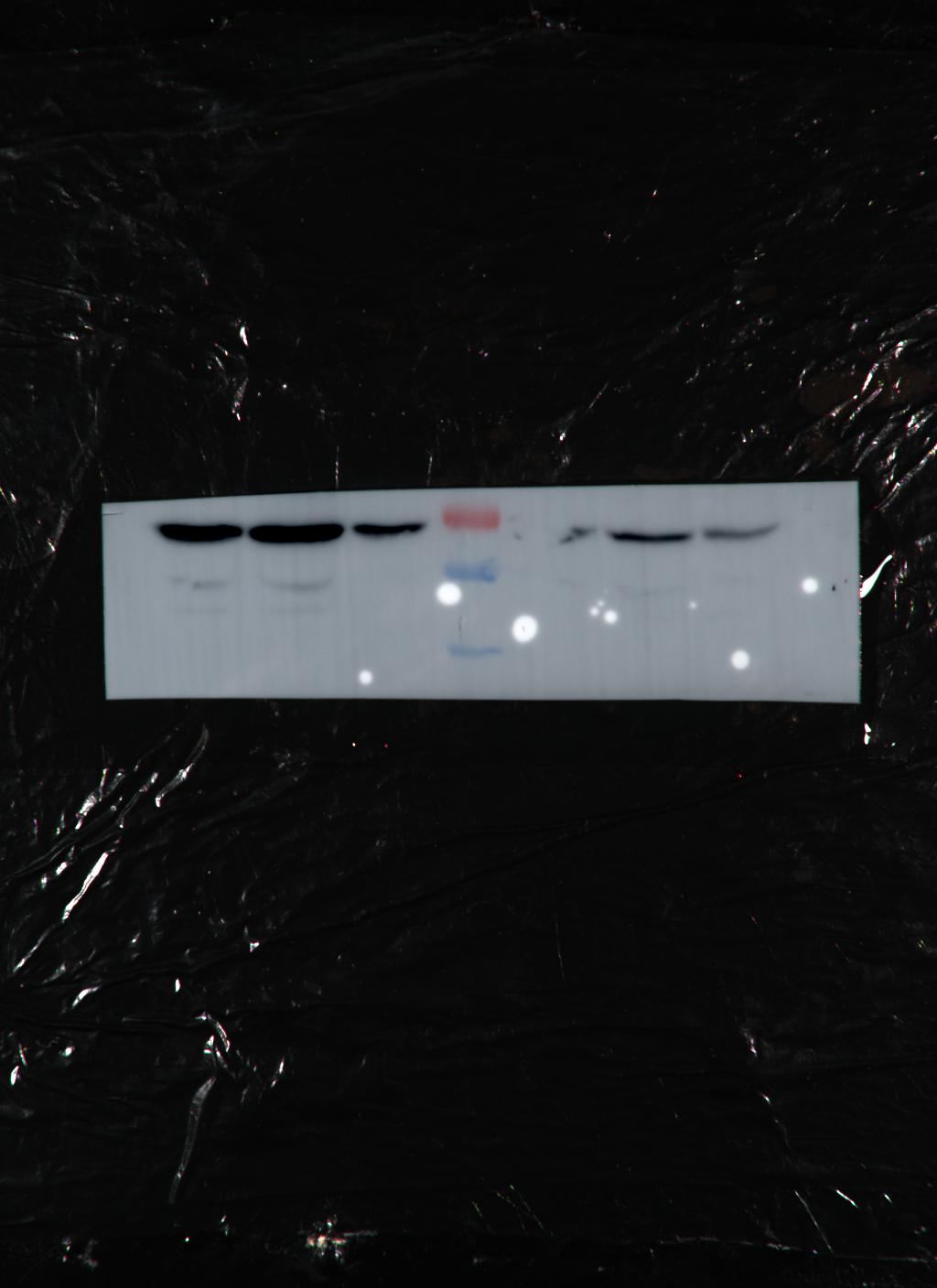

Supplement: Supplementary file 2 [file DataSheet2.zip › cell samples-WB supplementary materials/p-p65/PP65 1/pP65 +Marker.jpg]

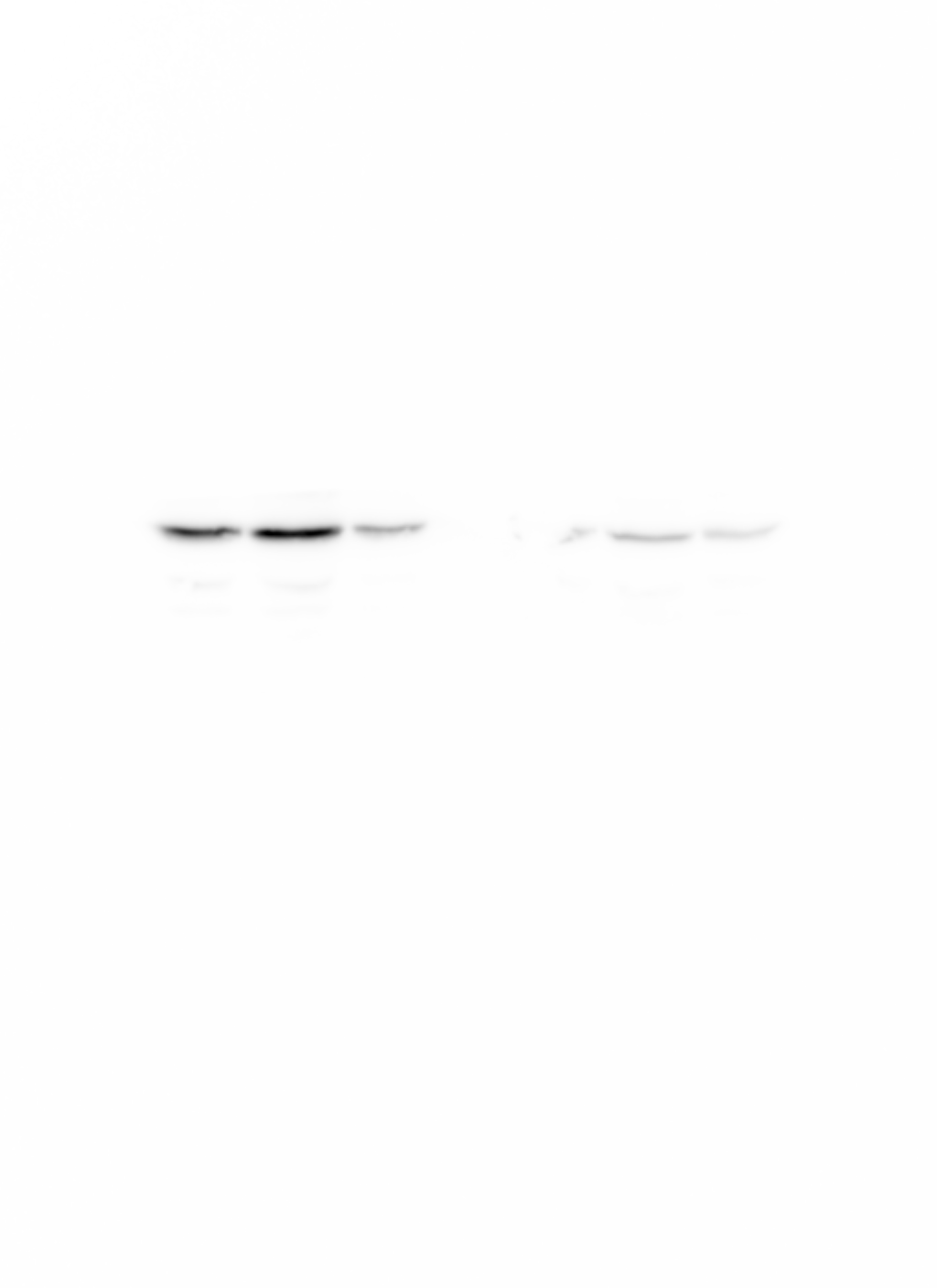

Supplement: Supplementary file 2 [file DataSheet2.zip › cell samples-WB supplementary materials/p-p65/PP65 1/pP65 .tif]

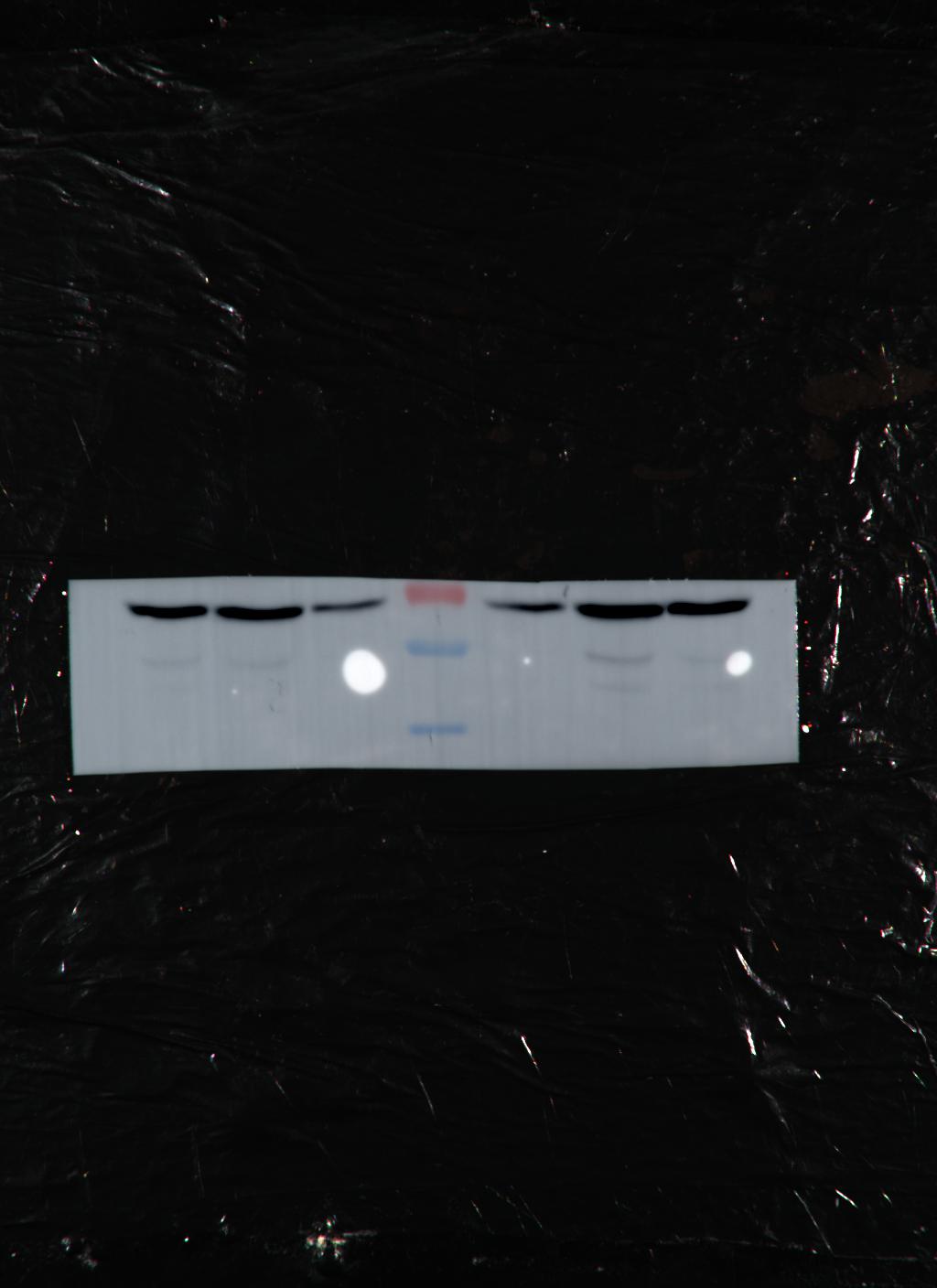

Supplement: Supplementary file 2 [file DataSheet2.zip › cell samples-WB supplementary materials/p-p65/PP65 2/pP65 +Marker.jpg]

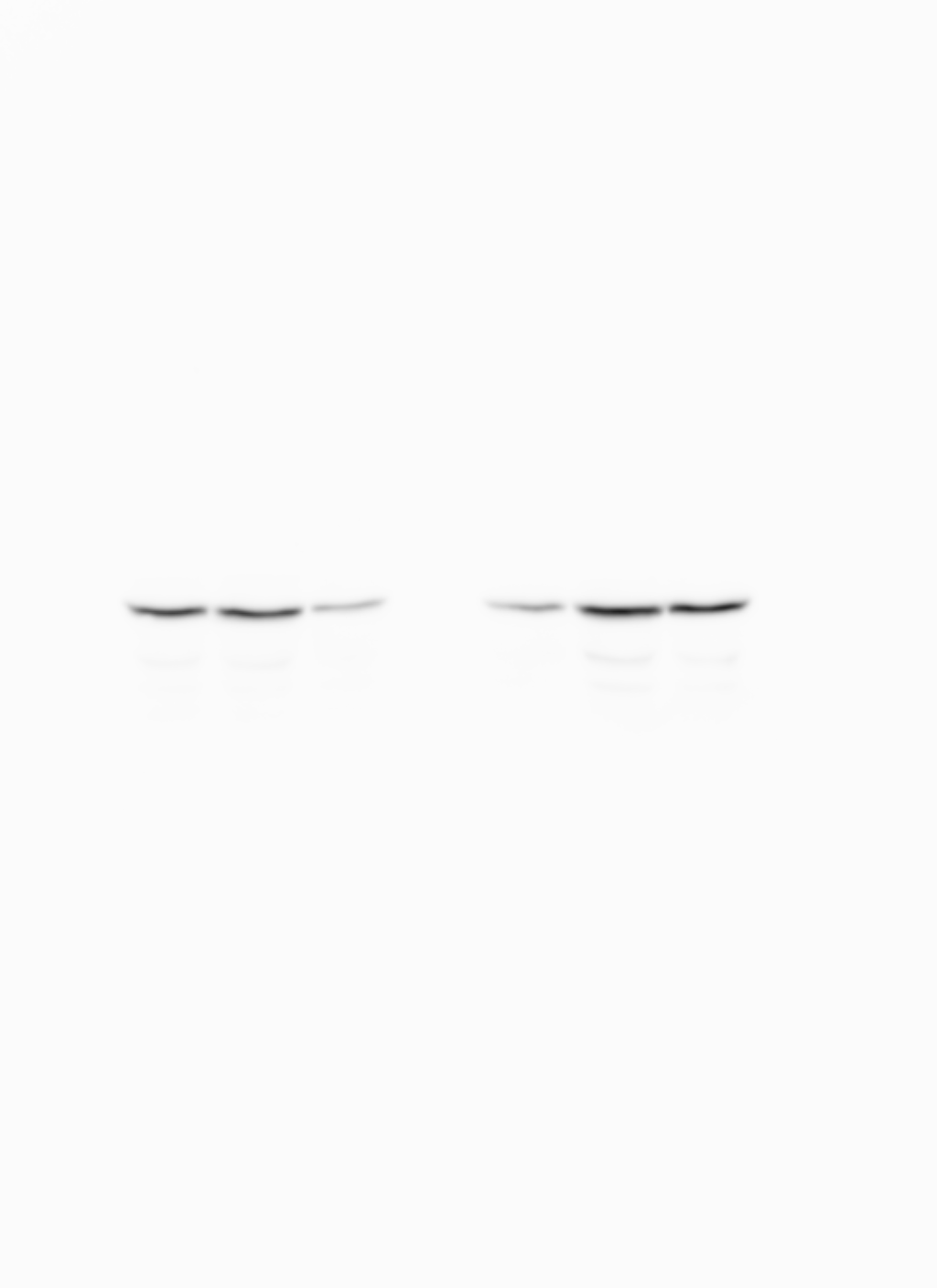

Supplement: Supplementary file 2 [file DataSheet2.zip › cell samples-WB supplementary materials/p-p65/PP65 2/pP65 .tif]

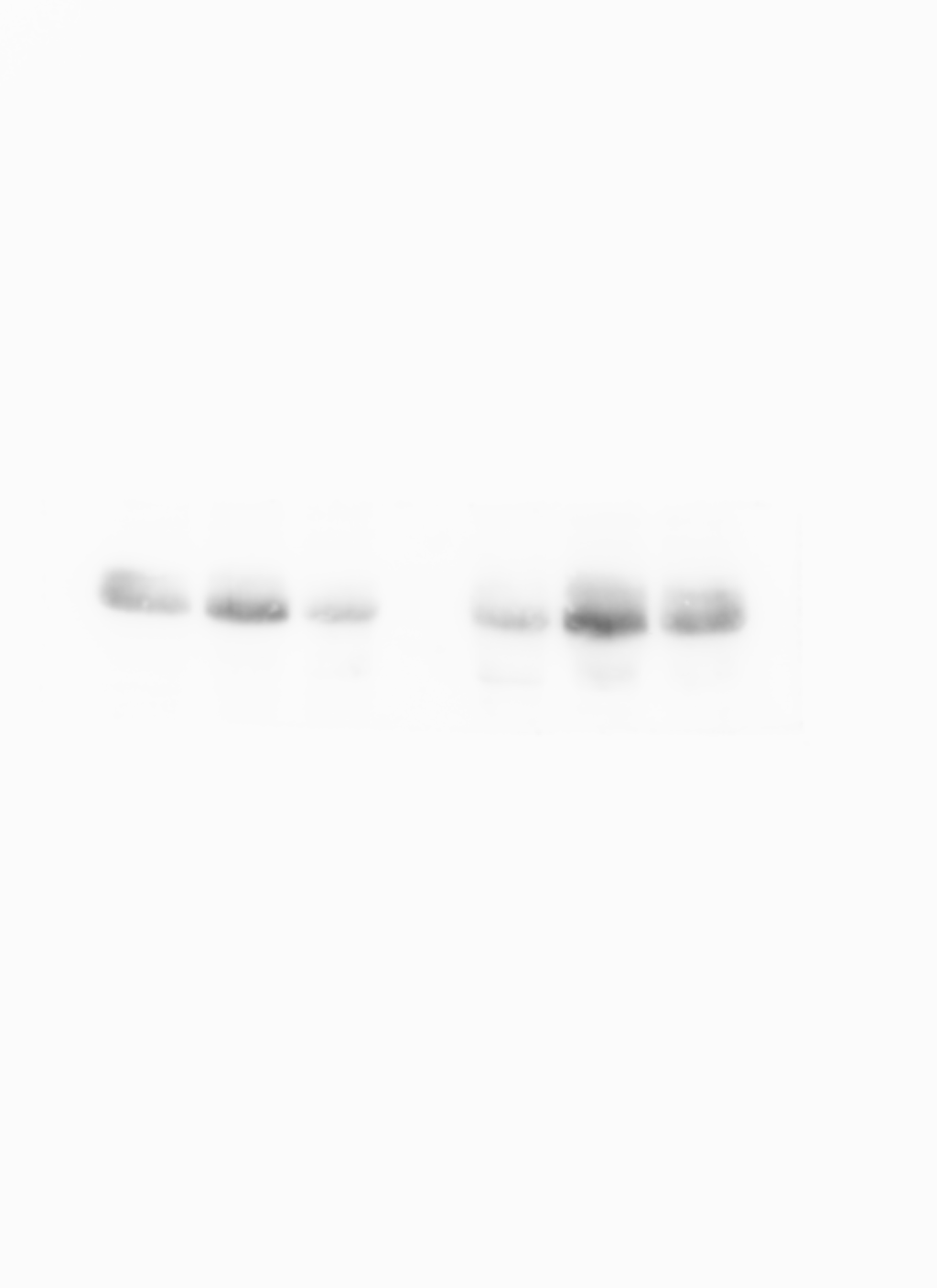

Supplement: Supplementary file 2 [file DataSheet2.zip › cell samples-WB supplementary materials/p-PAX/FY pax-S83 2/pax-S83 .tif]

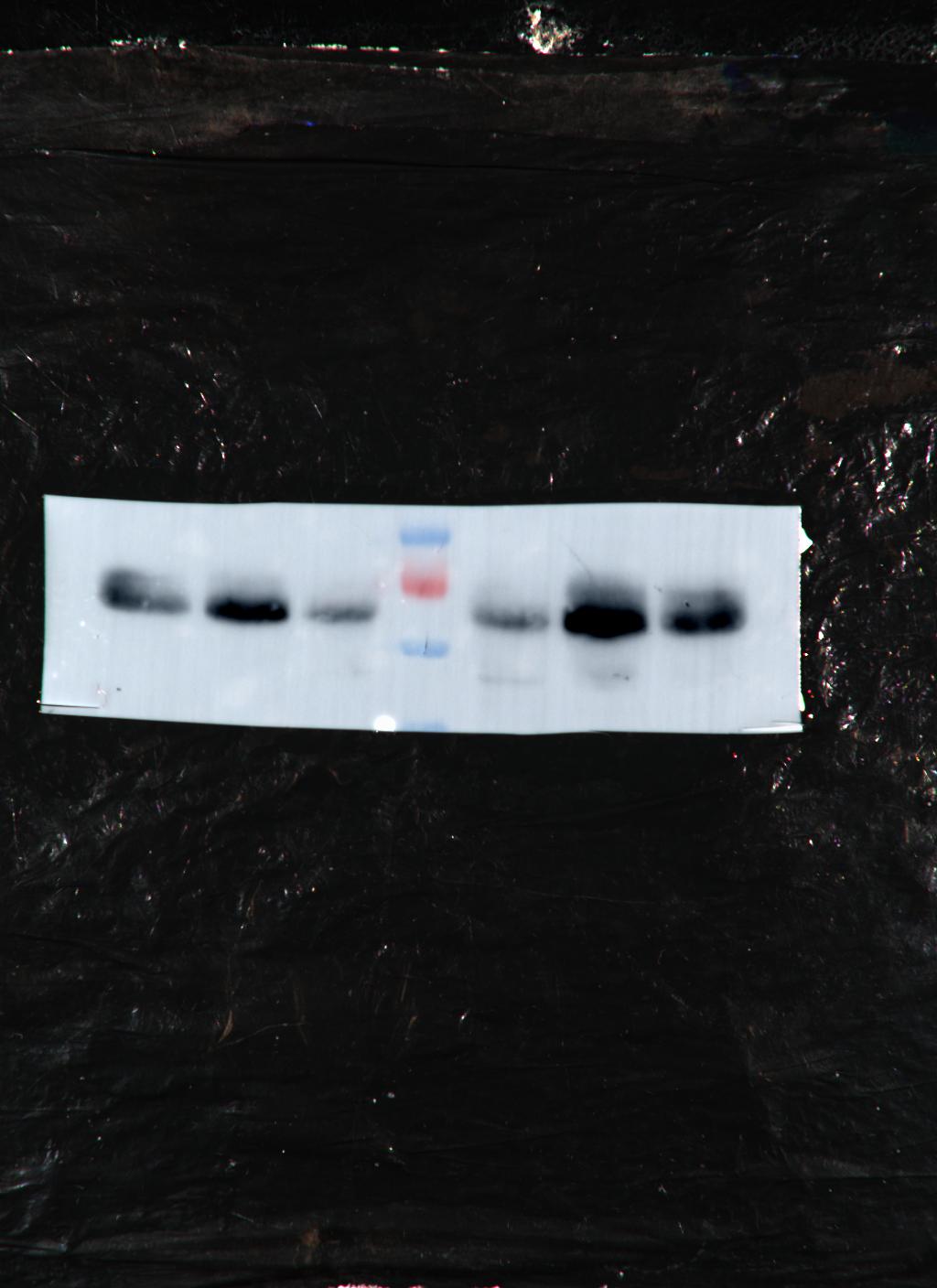

Supplement: Supplementary file 2 [file DataSheet2.zip › cell samples-WB supplementary materials/p-PAX/FY pax-S83 2/pax-S83+Marker.jpg]

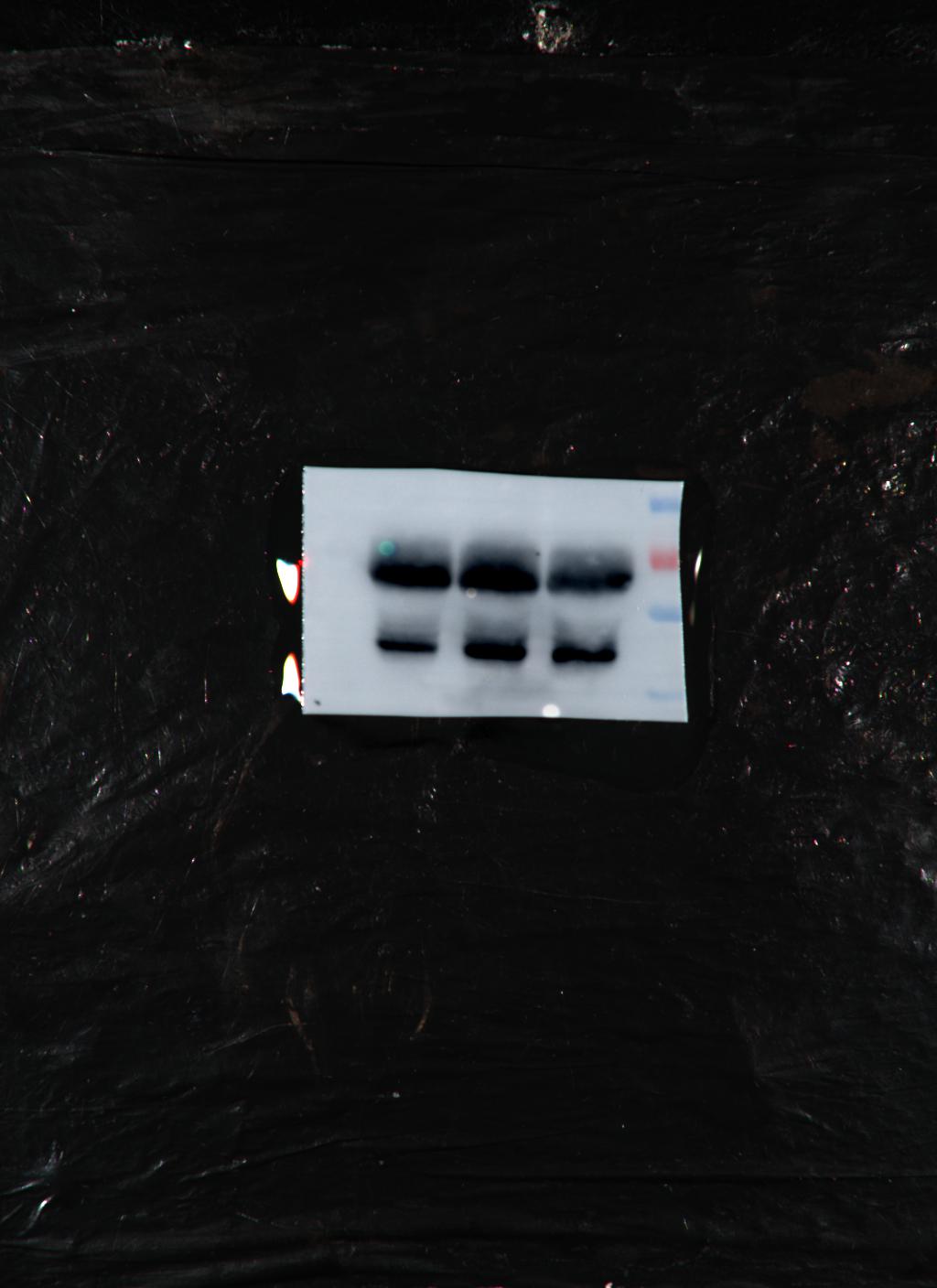

Supplement: Supplementary file 2 [file DataSheet2.zip › cell samples-WB supplementary materials/p-PAX/FY paxillin 1/paxillin +Marker.jpg]

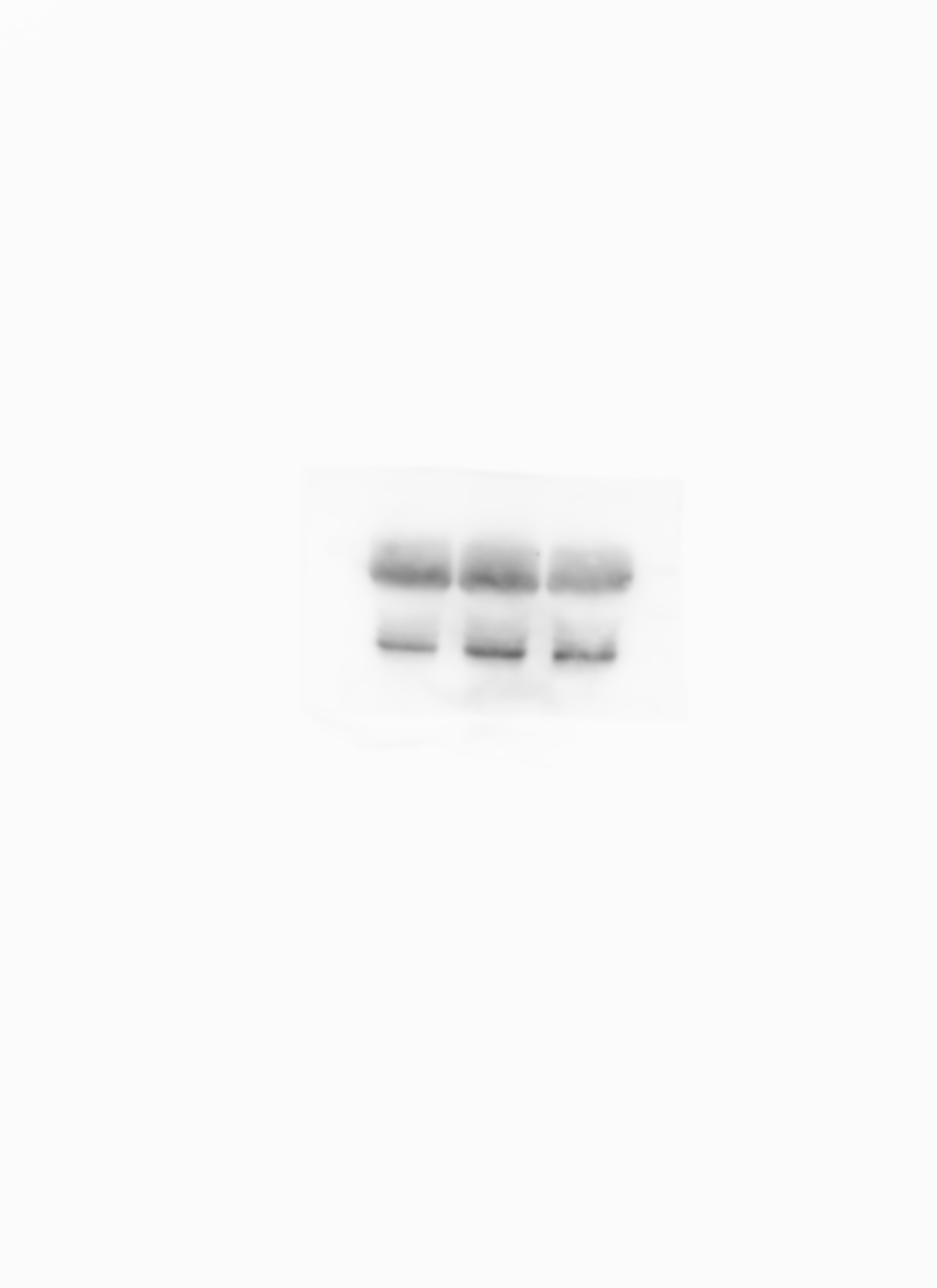

Supplement: Supplementary file 2 [file DataSheet2.zip › cell samples-WB supplementary materials/p-PAX/FY paxillin 1/paxillin .tif]

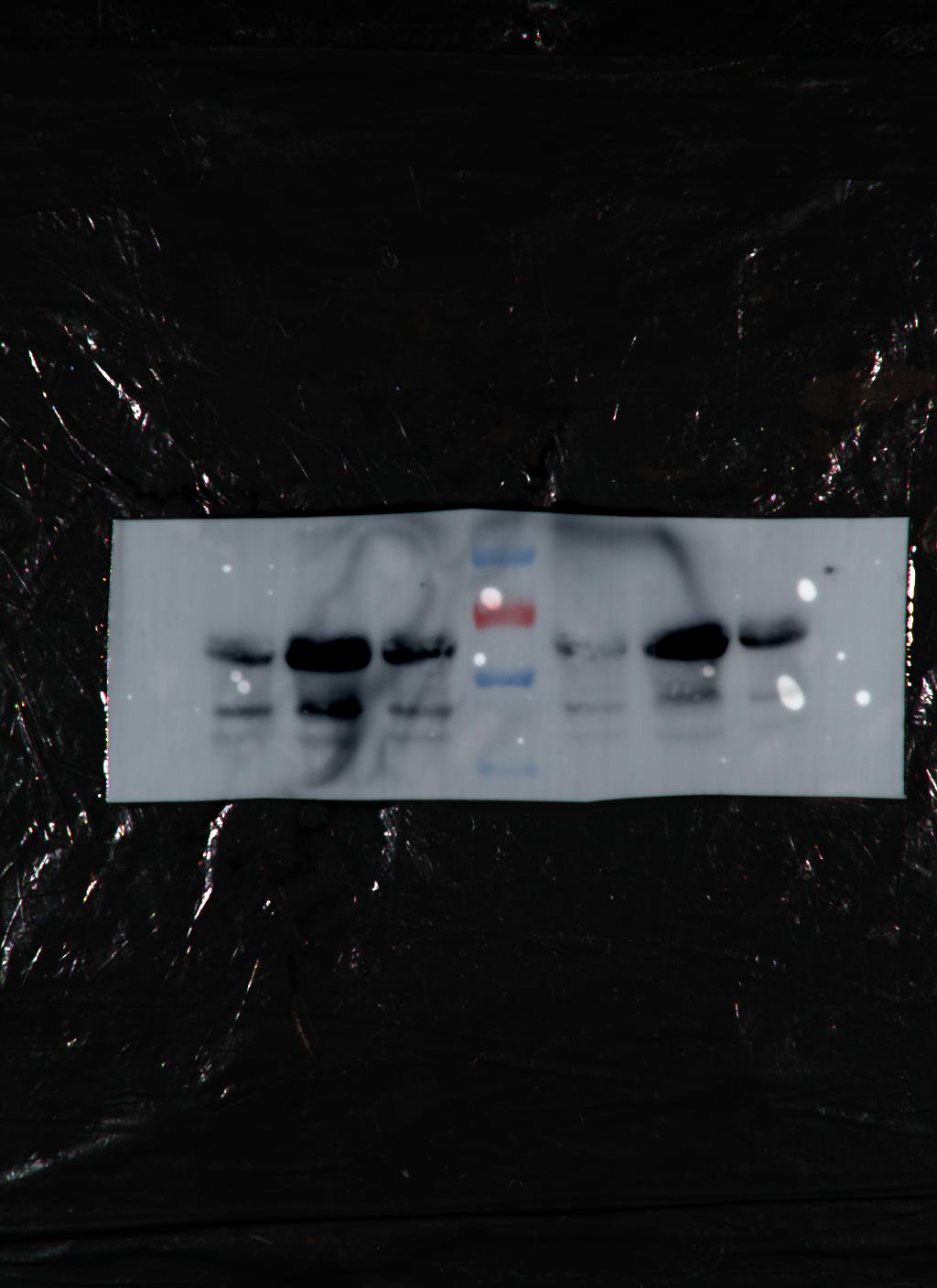

Supplement: Supplementary file 2 [file DataSheet2.zip › cell samples-WB supplementary materials/p-PAX/FY paxillin s83 1/paxillin s83 +Marker.jpg]

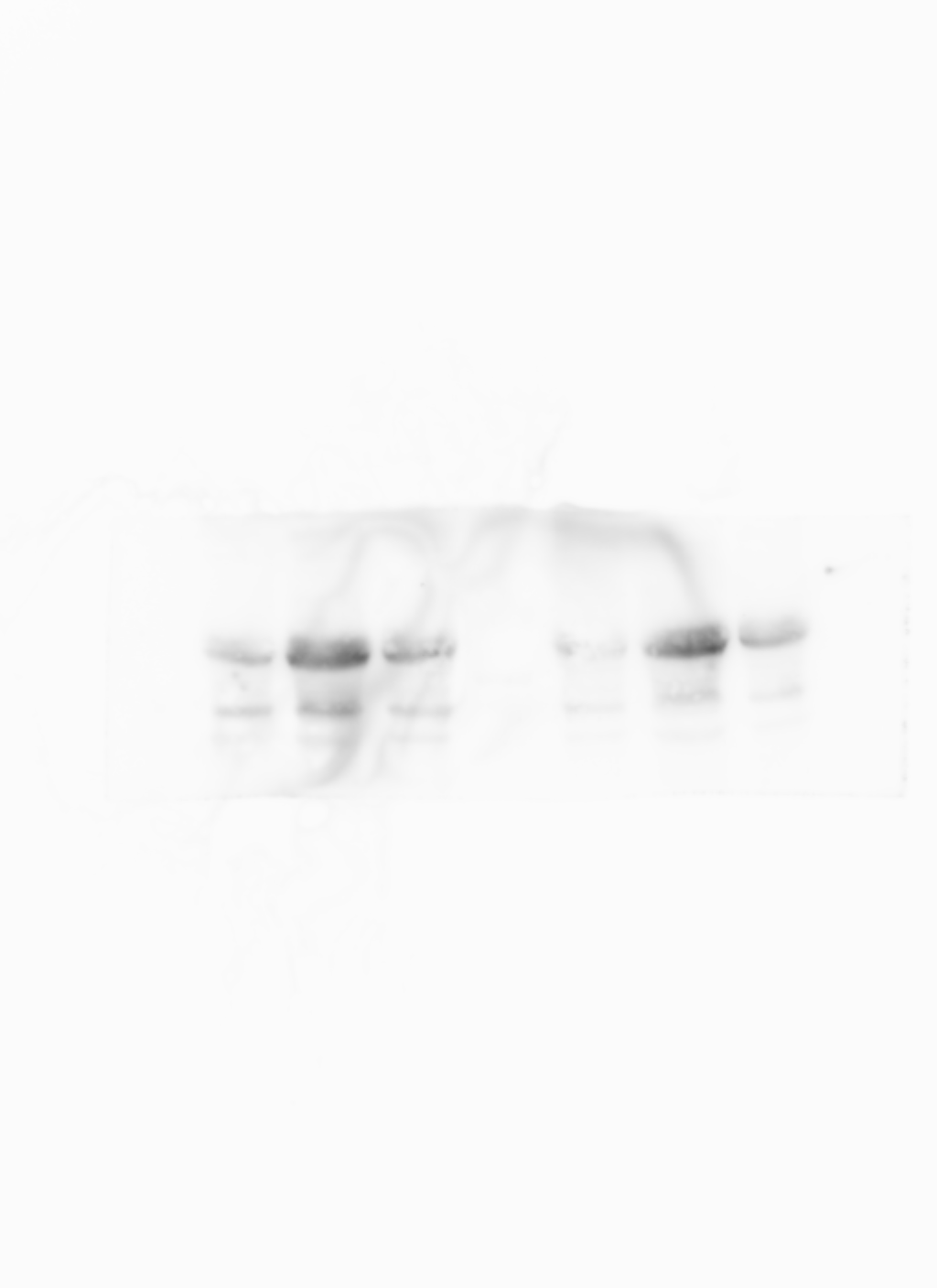

Supplement: Supplementary file 2 [file DataSheet2.zip › cell samples-WB supplementary materials/p-PAX/FY paxillin s83 1/paxillin s83 .tif]
